# Multi-Center Randomized Phase II Study Comparing Cediranib plus Gefitinib with Cediranib plus Placebo in Subjects with Recurrent/Progressive Glioblastoma

**DOI:** 10.1371/journal.pone.0156369

**Published:** 2016-05-27

**Authors:** Nicholas Brown, Catherine McBain, Stephen Nash, Kirsten Hopkins, Paul Sanghera, Frank Saran, Mark Phillips, Fiona Dungey, Laura Clifton-Hadley, Katharina Wanek, Daniel Krell, Sarah Jeffries, Iftekhar Khan, Paul Smith, Paul Mulholland

**Affiliations:** 1 University College London Hospitals, London, United Kingdom; 2 The Christie NHS Foundation Trust, Manchester, United Kingdom; 3 Cancer Research UK and UCL Cancer Trials Centre, London, United Kingdom; 4 Bristol Haematology and Oncology Centre, Bristol, United Kingdom; 5 Hall Edwards Radiotherapy Research Group, University Hospital Birmingham, Birmingham, United Kingdom; 6 Department of Radiotherapy and Paediatric Oncology, Royal Marsden NHS Trust, Sutton, United Kingdom; 7 Department of Academic Oncology, Royal Free Hospital, London, United Kingdom; 8 Cambridge University Hospitals NHS Foundation Trust, Cambridge, United Kingdom; 9 UCL Cancer Institute, University College London, London, United Kingdom; Catalan Institute of Oncology, SPAIN

## Abstract

**Background:**

Cediranib, an oral pan-vascular endothelial growth factor (VEGF) receptor tyrosine kinase inhibitor, failed to show benefit over lomustine in relapsed glioblastoma. One resistance mechanism for cediranib is up-regulation of epidermal growth factor receptor (EGFR). This study aimed to determine if dual therapy with cediranib and the oral EGFR inhibitor gefitinib improved outcome in recurrent glioblastoma.

**Methods and Findings:**

This was a multi-center randomized, two-armed, double-blinded phase II study comparing cediranib plus gefitinib versus cediranib plus placebo in subjects with first relapse/first progression of glioblastoma following surgery and chemoradiotherapy. The primary outcome measure was progression free survival (PFS). Secondary outcome measures included overall survival (OS) and radiologic response rate. Recruitment was terminated early following suspension of the cediranib program. 38 subjects (112 planned) were enrolled with 19 subjects in each treatment arm. Median PFS with cediranib plus gefitinib was 3.6 months compared to 2.8 months for cediranib plus placebo (HR; 0.72, 90% CI; 0.41 to 1.26). Median OS was 7.2 months with cediranib plus gefitinib and 5.5 months with cediranib plus placebo (HR; 0.68, 90% CI; 0.39 to 1.19). Eight subjects (42%) had a partial response in the cediranib plus gefitinib arm versus five patients (26%) in the cediranib plus placebo arm.

**Conclusions:**

Cediranib and gefitinib in combination is tolerated in patients with glioblastoma. Incomplete recruitment led to the study being underpowered. However, a trend towards improved survival and response rates with the addition of gefitinib to cediranib was observed. Further studies of the combination incorporating EGFR and VEGF inhibition are warranted.

**Trial Registration:**

ClinicalTrials.gov NCT01310855

## Introduction

Glioblastoma (GBM) is the most common malignant primary brain tumor, with around 2,200 cases diagnosed each year in England, and over 11,500 cases diagnosed annually in the USA [1, 2]. The standard treatment of surgery followed by radiotherapy with concurrent and adjuvant temozolomide chemotherapy results in median survival of only 14.6 months [3, 4]. No clinical trials have demonstrated an improvement in survival with any second-line therapies. Improved treatments are required both in the first-line setting and at relapse.

The epidermal growth factor receptor (EGFR) (ErbB-1) is a member of a family of 4 structurally related membrane spanning receptor tyrosine kinases including ErbB-2 (HER-2) ErbB-3 and ErbB-4. These receptors are activated with varying efficacy by epidermal growth factor ligands. Receptor-ligand interaction leads to dimer formation and activation, which stimulates the intrinsic intracellular protein tyrosine kinase activity. The downstream targets of EGFR include signaling proteins with important roles in cell lineage determination and cell survival. Mutations leading to the over-expression or amplification of EGFR contribute to oncogenesis by inducing cells to proliferate and to resist apoptosis. A variety of mutations affecting the expression and function of this family of receptors have been demonstrated in a variety of cancers [5]. In 40–50% of cases of glioblastoma, EGFR is over-expressed; co-expression of the constitutively activated mutant variant of EGFR, the epidermal growth factor variant III (EGFRvIII) is observed in nearly half of these cases [6–11]. The epidermal growth factor receptor variant III (EGFRvIII) is the product of a common, tumor-specific mutation of EGFR consisting of an in-frame deletion of exons 2–7 (801bp) from its extracellular ligand-binding region.

Angiogenesis is the process through which new blood vessels are formed. Glioblastomas are profusely vascular tumors featuring prolific levels of angiogenesis and this is in part attributable to their high levels of vascular endothelial growth factor (VEGF) expression [12]. Pathologically, glioblastomas are distinguished from low-grade gliomas by microvascular hyperplasia and focal necrosis. The aberrant vasculature that characterizes high-grade gliomas is induced by a hypoxic microenvironment which in turn drives the expression of hypoxia inducible factor (HIF-1). This leads to the secretion of pro-angiogenesis factors (including VEGF and IL-8). Hypoxia also drives the local expression of tissue factor that initiates pro-thrombotic conditions and these in turn contribute to the anomalous processes of neo-vascularization that define glioblastomas[13, 14]. Disrupting the processes involved in angiogenesis is part of the management of many tumor types including glioblastoma [15–18].

Cediranib is a potent, orally available small molecule inhibitor of the vascular endothelial growth factor receptor (VEGFR) tyrosine kinases [19]. Cediranib is active against each variant of VEGFR with particular potency against VEGFR-2, the main mediator in endothelial cell proliferation, differentiation and vascular permeability. It also has activity against both platelet derived growth factor receptor (PDGFR) and the c-Kit receptor, which are implicated in angiogenesis and cell cycle regulation respectively. However, cediranib failed to improve outcome in recurrent glioblastoma in a randomized phase III trial comparing cediranib versus cediranib and lomustine versus lomustine alone [20].

Gefitinib is an orally administered, small molecule inhibitor of the EGFR tyrosine kinase. It competes with adenosine triphosphate (ATP) for binding sites on the catalytic domain of the receptor, inhibiting auto-phosphorylation and activation. Phase II studies using single agent gefitinib in recurrent glioblastoma have demonstrated only modest activity in genetically unselected populations [21, 22].

In this investigational study we propose the use of a combination of therapies targeting the VEGF (cediranib) and EGFR (gefitinib) pathways in patients with recurrent glioblastoma, a group of patients with a clearly unmet clinical need. The combination of cediranib and gefitinib has been explored in a phase I study in solid tumors which demonstrated that combination treatment was generally well tolerated and showed encouraging antitumor activity. No patients enrolled had glioblastoma. [23].

## Patients and Methods

### Ethics

This study was conducted in accordance with the Declaration of Helsinki, the principles of Good Clinical Practice (GCP) and UK Clinical Trials Regulations. The study protocol was approved by the London—Harrow Research Ethics Committee (10/H0715/77) and the Medicines and Healthcare Regulatory Agency (MHRA). All participating sites had local approval to carry out the trial. All participants provided written informed consent prior to participation in the study. The trial protocol and supporting CONSORT checklist are available as supporting information (S1 CONSORT Checklist and S1 Protocol). The trial was registered with Clinicaltrials.gov (NCT01310855).

### Study design and patients

We conducted a phase II, randomized, parallel, double blind, placebo controlled, multi-center trial in the UK. Patients were recruited from seven hospitals. To be eligible for recruitment, patients were required to have measurable disease on MRI following standard first-line treatment for histologically-confirmed glioblastoma: surgery (unless resection impossible due to anatomical location based on neurosurgeon’s assessment), followed by cranial radiotherapy (completed at least 3 months prior to enrolment) and chemotherapy with concurrent and adjuvant temozolomide (last dose at least 28 days prior to enrolment). Any other prior treatment (with the exception of steroids) made the patient ineligible for this trial.

Other eligibility criteria were age ≥ 18 years; life expectancy ≥ 12 weeks; Karnofsky Performance Score ≥ 70; mini-mental status examinations core ≥ 15; presence of measurable tumor seven days prior to enrolment (contrast enhancing tumor ≥ 10mm by shortest distance on two axial slices); stable dose of steroids (≤ 8mg/day) for at least five days prior to baseline MRI; adequate bone marrow reserve (absolute neutrophil count > 1.5 × 10^9^ /L); serum bilirubin < 1.5 × ULRR; ALT and AST < 5 × ULRR.

Exclusion criteria included the use of enzyme-inducing anti-epileptic drugs within two weeks prior to enrolment, pregnancy or breast-feeding, and infection with HIV or hepatitis B or C.

Randomization was performed in the ratio 1:1 using a minimization algorithm stratified by the dichotomous factors of age (<65 / ≥65), Karnofsky Performance Status (≤80 / >80), and previous resection (yes/no). The randomization system was managed independent of the trial management team. Upon receipt of a registration fax from the recruiting site trial staff would use an online randomization system to produce container numbers for the assigned treatment. The site would then allocate the medication to the patient. The contents of the bottles were concealed from site staff, patients, and trial management.

### Treatment

Patients were randomized to receive cediranib plus gefitinib or cediranib plus placebo. All patients received 30mg cediranib (AZD2171) orally every day along with a daily oral dose of either 500mg gefitinib or a matched placebo. Dose selection was based on reported toxicity and the maximum tolerated dose in the phase 1 study of cediranib in combination with gefitinib [23]. Treatment continued until unacceptable toxicity, patient decision, or progression (although treatment could continue if the investigator was of the opinion that the patient was benefitting). If cediranib or gefitinib was not tolerated it was permitted to reduce the dose by one or more levels: cediranib to 20mg or 15mg, and gefitinib to 250mg.

### Endpoints

The primary endpoint was progression free survival (PFS) which was defined as the time from randomisation to first progression or death from any cause, whichever came first. Response and progression were assessed by clinical assessment and MRI scans every six weeks, using the modified RANO criteria [24]. A secondary analysis was performed on overall survival (OS), PFS at six months, and OS at 12 months.

Quality of Life was assessed using the EORTC QLQ-C30 and the EORTC brain tumor module BN-20. Patients were invited to complete forms at baseline, at six-weekly intervals, and at discontinuation of treatment.

All Adverse Events and Serious Adverse Events were recorded using the CTCAE v4.03.

### Statistical Analysis

Recruitment of 112 patients was planned to achieve 80% power to detect a hazard ratio of 0.67 with a one-sided type I error of 10%. This assumed a median PFS of 4 months in the cediranib plus placebo arm and 6 months in the combination arm with an accrual period of 18 months and a maximum follow-up time of 36 months. The hazard in the cediranib plus placebo group was assumed to be 0.115. Sample size calculations were performed using nQuery version 7.0.

Progression free and overall survival were assessed via Kaplan-Meier plots and Cox proportional hazard models adjusted for the randomization strata of age, previous resection and Karnofsky Performance Status.

All statistical results report 90% confidence intervals for point estimates and one-sided p-values with a threshold of 0.1 used to assess statistical significance. Analyses were conducted on the Intention to Treat (ITT) population defined as all patients randomized to treatment. For toxicity, all SAEs of grade 3 or higher were summarized by treatment group in the Safety population only, defined as all patients who took at least one dose of randomized treatment.

Quality of life was assessed on all EORTC QLQ-C30 and the EORTC BN-20 subscales. Data for the later time points was sparse so the decision was taken to analyze only the change in scores from baseline to week 6. The treatment effect was estimated using a regression model adjusting for baseline score. Results are presented with 99% confidence intervals to account for multiple comparisons.

All statistical analyses were performed using Stata version 12.1.

## Results

### Patients

**Fig 1:**
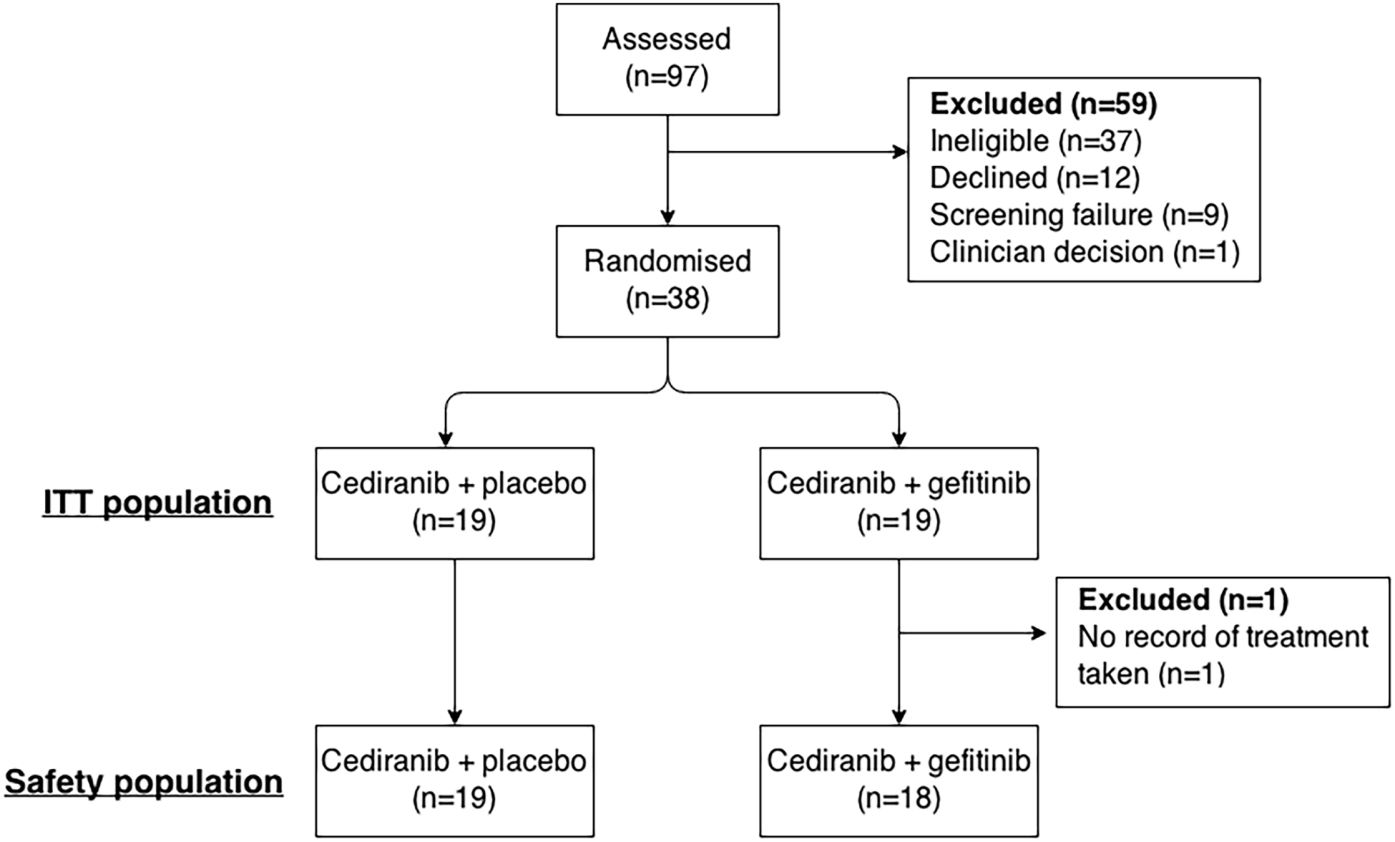
CONSORT diagram.

A total of 97 patients were screened at eight study sites. Of these, 38 patients from seven hospitals were enrolled between May 2011 and August 2012 and randomized to receive cediranib plus gefitinib (n = 19) or cediranib plus placebo (n = 19) (Fig 1). AstraZenica discontinued development of cediranib during recruitment to the trial. Recruitment to the trial was thus terminated prematurely in August 2012. This reduced the effective statistical power to 47% (using the assumptions above) and so the results can be considered as exploratory only. Patients who were enrolled prior to termination of recruitment received treatment as per protocol.

Table 1 shows the baseline characteristics, which were well balanced between the two arms. In the cediranib plus gefitinib arm the median age was younger and a higher proportion of patients had had surgery for recurrent disease. At the time of this analysis, all patients have died, with a maximum follow-up time of 16.7 months. One patient on the cediranib plus gefitinib arm did not complete their medication diary and thus we are not able to confirm that they took any of the randomized medication. This patient was excluded from the Safety population analysis as per the protocol. There was no other missing patient information. EGFR analysis was planned but not performed after premature termination of the trial, as inadequate results were anticipated due to the limited number of patients.

**Table 1:** Patient characteristics at baseline for the ITT population.

	CED only (N = 19)	CED+GEF (N = 19)	All patients (N = 38)
**Sex**			
Male; N (%)	14 (73.7%)	13 (68.4%)	27 (71.1%)
Female; N (%)	5 (26.3%)	6 (31.6%)	11 (28.9%)
**Age at entry**			
Under 65 at entry; N (%)	15 (78.9%)	15 (78.9%)	30 (78.9%)
65 or over at entry; N (%)	4 (21.1%)	4 (21.1%)	8 (21.1%)
**Age at entry; median (range)**	61.0 (40–69)	55.0 (30–71)	57.0 (30–71)
**Resection for the recurrent disease**			
No resection; N (%)	15 (78.9%)	17 (89.5%)	32 (84.2%)
Previous resection; N (%)	4 (21.1%)	2 (10.5%)	6 (15.8%)
**Karnofsky Performance Status**			
Median (range)	90 (70–100)	90 (80–100)	90 (70–100)
KPS < = 80; N (%)	6 (31.6%)	6 (31.6%)	12 (31.6%)
KPS > 80; N (%)	13 (68.4%)	13 (68.4%)	26 (68.4%)
**Time from diagnosis to randomisation** (months); median (range)	10.2 (6.0–27.4)	12.7 (6.7–29.4)	12.1 (6.0–29.4)

### Efficacy

**Fig 2:**
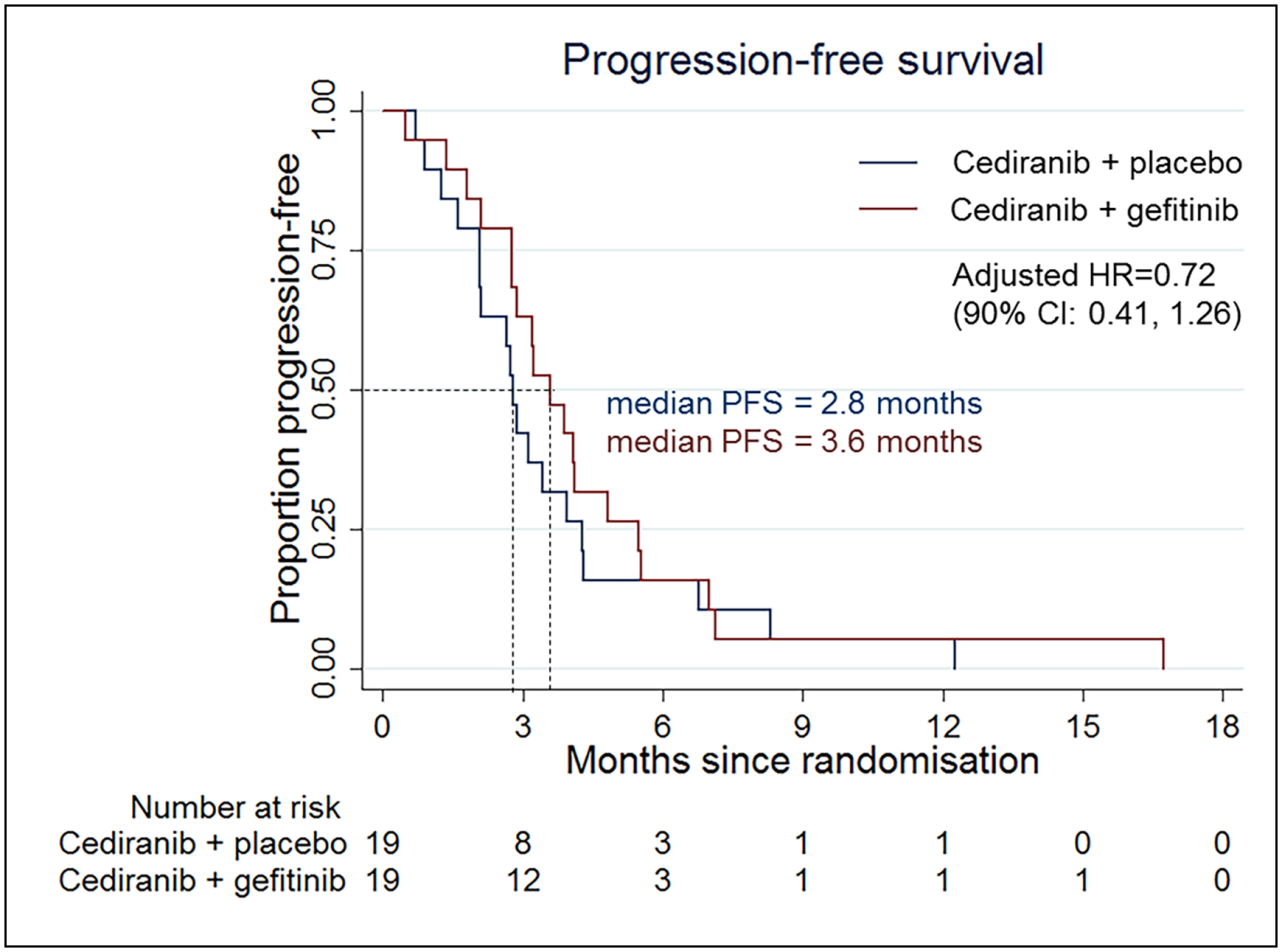
Kaplan-Meier plot for PFS in the ITT population.

All patients had died within 24 months of randomization; all but one with confirmed progression. This patient suffered a fatal pulmonary embolism. The median PFS for patients on the cediranib plus gefitinib arm was 3.6 months compared to 2.8 months for patients in the cediranib plus placebo group (HR; 0.72, 90% CI; 0.41 to 1.26) (Fig 2). Excluding the one patient who did not take the randomized treatment had no material effect on the hazard ratio (0.67, 90% CI 0.38 to 1.18), nor did using age and KPS as continuous measures (0.76, 90% CI 0.43 to 1.34). The progression-free proportions at six months were identical in the two arms (15.8%). Full results from the Cox proportional hazard model are in Table 2.

**Table 2:** Cox regression model for PFS.

	n	Hazard ratio (90% CI)	P-value
**Treatment**			
Cediranib only	19	1 (reference)	0.17
Cediranib + gefitinib	19	0.72 (0.41, 1.26)	
**Age at entry over 65**			
Under 65 at entry	30	1 (reference)	0.02
65 or over at entry	8	2.52 (1.19, 5.33)	
**Resection for the recurrent disease**			
No resection	32	1 (reference)	0.04
Previous resection	6	2.54 (1.08, 5.98)	
**Karnofsky performance status**			
< = 80	12	1 (reference)	0.50
> 80	26	1.00 (0.52, 1.90)	

**Fig 3:**
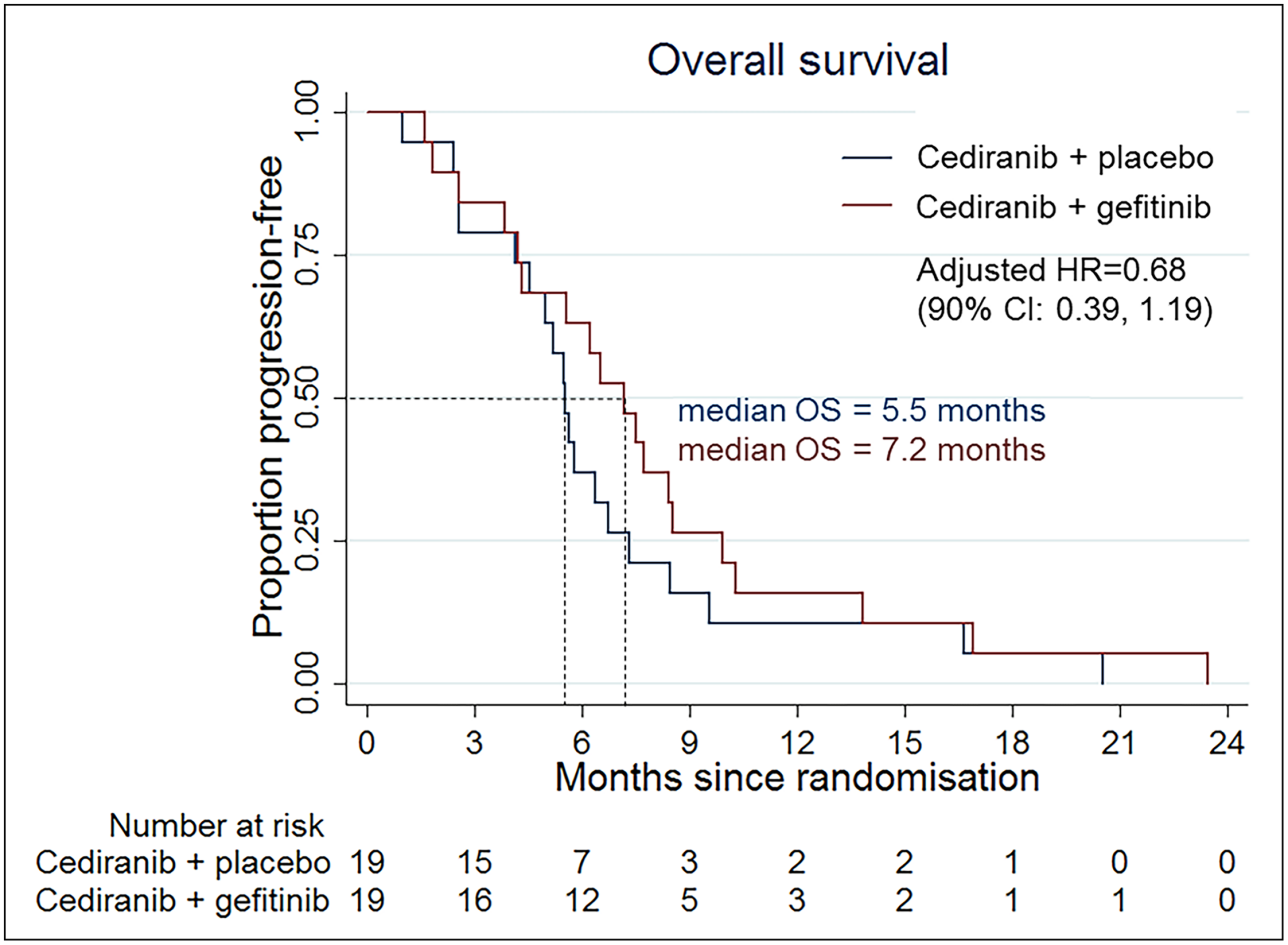
Kaplan-Meier plot for OS in the ITT population

The median survival times were 7.2 months in the cediranib plus gefitinib arm and 5.5 months in the cediranib plus placebo arm. The adjusted hazard ratio for overall survival was 0.68 (90% CI; 0.39 to 1.19) in favor of the cediranib plus gefitinib arm (Fig 3). The 12-month survival proportions were 15.8% in the cediranib plus gefitinib arm versus 10.5% in the control arm. The median PFS and OS of the whole population were 3.1 and 5.8 months. There were no clinical complete responses among the 38 patients. In the cediranib plus gefitinib arm there were eight patients (42%) with a partial response versus five patients (26%) in the cediranib plus placebo arm.

### Quality of life

Quality of life analysis was performed on the 26 patients who completed forms at both baseline and at week 6. In some cases patients did not complete all questions and so could not be included in all subscales. All results are based on data from at least 23 patients and are summarized in Table 3. None of the results provide evidence that the addition of gefitinib resulted in a deterioration of quality of life.

**Table 3:** Comparison of quality of life changes between baseline and week six.

Scale	Mean in control arm at baseline (SD)	Mean in treatment arm at baseline (SD)	Mean in control arm at Week 6 (SD)	Mean in control arm at Week 6 (SD)	Treatment effect (99% CI)^a^
Global health status (N = 26)	66.0 (15.7)	67.3 (16.5)	62.5 (21.5)	61.3 (23.9)	-2.3 (-22.8, 18.2)
**C30 Functional scales**^b^	**(higher score indicates increased functioning)**				
Physical functioning (N = 26)	71.7 (25.1)	83.8 (18.8)	72.8 (19.2)	75.7 (22.3)	-4.0 (-23.7, 15.7)
Role functioning (N = 26)	61.1 (33.6)	78.6 (22.1)	55.6 (27.8)	69.0 (23.4)	8.3 (-20.3, 36.9)
Emotional functioning (N = 26)	72.2 (20.2)	73.8 (20.6)	71.5 (19.3)	78.0 (22.3)	6.1 (-16.9, 29.1)
Cognitive functioning (N = 26)	63.9 (23.4)	69.0 (26.0)	63.9 (27.4)	67.9 (31.0)	-0.4 (-23.4, 22.6)
Social functioning (N = 26)	63.9 (30.8)	72.6 (21.3)	69.4 (23.4)	73.8 (32.5)	-0.6 (-28.7, 27.5)
**C30 Symptom scale**^c^	**(higher score indicates worsening symptoms)**				
Fatigue (N = 25)	38.4 (26.9)	27.8 (11.3)	41.4 (22.8)	39.7 (27.8)	4.0 (-24.2, 32.2)
Nausea and vomiting (N = 26)	1.4 (4.8)	1.2 (4.5)	2.8 (6.5)	6.0 (8.3)	3.2 (-5.2, 11.6)
Pain (N = 26)	18.1 (26.1)	13.1 (16.2)	13.9 (27.4)	17.9 (24.0)	6.7 (-19.1, 32.5)
Dyspnoea (N = 25)	24.2 (26.2)	9.5 (15.6)	15.2 (22.9)	19.0 (25.2)	15.4 (-6.6, 37.4)
Insomnia (N = 26)	36.1 (43.7)	26.2 (29.8)	36.1 (36.1)	21.4 (28.1)	-10.7 (-43.0, 21.6)
Appetite loss (N = 26)	11.1 (21.7)	2.4 (8.9)	22.2 (25.9)	19.0 (25.2)	-5.1 (-34.9, 24.7)
Constipation (N = 25)	12.1 (22.5)	9.5 (15.6)	9.1 (21.6)	14.3 (21.5)	6.0 (-18.2, 30.2)
Diarrhoea (N = 26)	11.1 (21.7)	2.4 (8.9)	44.4 (32.8)	40.5 (26.7)	-6.4 (-40.6, 27.8)
Financial difficulties (N = 26)	22.2 (32.8)	23.8 (30.5)	25.0 (35.2)	11.9 (21.1)	-14.1 (-37.1, 8.9)
**B20 Symptom scales (c)**	**(higher score indicates worsening symptoms)**				
Future uncertainty (N = 25)	39.6 (22.8)	37.2 (20.9)	33.6 (17.5)	35.3 (31.6)	2.8 (-24.8, 30.4)
Visual disorder (N = 25)	19.4 (22.3)	17.1 (21.6)	14.8 (24.3)	14.5 (19.4)	1.7 (-12.7, 16.1)
Motor dysfunction (N = 25)	25.0 (22.3)	26.9 (24.0)	20.4 (17.5)	25.6 (33.1)	3.7 (-18.0, 25.4)
Communication deficit (N = 25)	23.1 (19.2)	25.6 (21.9)	25.0 (19.0)	18.8 (25.8)	-7.8 (-28.9, 13.3)
Headaches (N = 25)	25.0 (25.1)	28.2 (26.7)	8.3 (15.1)	10.3 (21.0)	0.9 (-18.3, 20.1)
Seizures (N = 23)	12.1 (16.8)	5.6 (13.0)	9.1 (21.6)	2.8 (9.6)	-3.6 (-22.7, 15.5)
Drowsiness (N = 25)	30.6 (22.3)	38.5 (18.5)	33.3 (0.0)	41.0 (30.9)	4.4 (-19.8, 28.6)
Itchy skin (N = 23)	3.3 (10.5)	17.9 (22.0)	10.0 (16.1)	25.6 (38.9)	7.7 (-31.9, 47.3)
Hair loss (N = 25)	8.3 (15.1)	17.9 (32.2)	5.6 (13.0)	10.3 (25.0)	3.0 (-20.1, 26.1)
Weakness of legs (N = 24)	21.2 (27.0)	20.5 (29.0)	9.1 (15.6)	28.2 (38.1)	19.5 (-10.2, 49.2)
Bladder control (N = 25)	11.1 (21.7)	0.0 (0.0)	8.3 (15.1)	5.1 (12.5)	1.6 (-13.6, 16.8)

^a.^ Treatment effect is from a regression model of the change at six weeks (compared to baseline), adjusting for baseline score. It represents the difference in increase in the cediranib + gefitinib arm compared to the cediranib + placebo arm.

^b.^ A higher score indicates increased functioning, so a difference in means greater than zero indicates a beneficial effect of gefitinib.

^c.^ A higher score indicates worsening symptoms, so a difference in means of less than zero indicates a beneficial effect of gefitinib.

### Compliance and safety

Treatment duration was greater on the cediranib plus gefitinib arm (median 148 days) compared to the cediranib plus placebo arm (84 days). 10 patients reduced their dose of cediranib, 7 (39%) on the cediranib plus gefitinib arm versus 3 (16%) on the cediranib plus placebo arm. Four patients (22%) on the cediranib plus gefitinib arm reduced their dose of gefitinib. There were no dose reductions of the placebo. Adverse event data for all patients in the safety population is summarized in Table 4. 16 patients (89%) in the cediranib plus gefitinib arm reported a Grade 3 or 4 adverse event, compared to 13 patients (68%) in the cediranib plus placebo arm. All patients reported at least one adverse event of grade 1 or 2. The most frequent side effects were fatigue, hypertension and lymphopenia.

**Table 4:** Grade 3 & 4 toxicities in the safety population (CTCAE version 4.03).

Adverse event	CED-only (N = 19) *No*. *(%)*	CED+GEF (N = 18) *No*. *(%)*
Fatigue	4 (21)	6 (33)
Hypertension	1 (5)	5 (28)
Lymphopenia	2 (11)	3 (17)
Anorexia	1 (5)	3 (17)
Ataxia	-	4 (22)
Dysphasia	2 (11)	1 (6)
Headache	1 (5)	2 (11)
Rash Pustular	1 (5)	2 (11)
Alanine aminotransferase increased	2 (11)	-
Pain	1 (5)	1 (6)
Fall	2 (11)	-
Thromboembolic event	1 (5)	1 (6)
Eye disorder	-	2 (11)
Hyperglycemia	1 (5)	1 (6)
Cognitive Disturbance	-	2 (11)
Muscle weakness right-sided	1 (5)	1 (6)
Sepsis	-	2 (11)
Aspartate aminotransferase increased	1 (5)	-
Blurred vision	-	1 (6)
Diarrhoea	-	1 (6)
Generalised muscle weakness	-	1 (6)
Haemorrhage	1 (5)	-
Infection	-	1 (6)
Mucositis Oral	-	1 (6)
Seizure	-	1 (6)
Stomatitis	1 (5)	-
Confusion	-	1 (6)
Skin ulceration	-	1 (6)
Musculoskeletal and connective tissue disorder	-	1 (6)
Cholesterol High	1 (5)	-
Nervous system disorders	-	1 (6)
Hypertrigylceridomia	1 (5)	-
Movements involuntary	-	1 (6)
Paresthesia	-	1 (6)
GGT increased	1 (5)	-
Cushingoid	1 (5)	-

## Discussion

In this randomized phase II trial we aimed to evaluate the rational combination of dual inhibition of VEGFR and EGFR in patients with recurrent glioblastoma. While our trial was ongoing, clinical development of cediranib was discontinued when the results of a phase III trial failed to demonstrate a survival benefit of cediranib over lomustine in recurrent glioblastoma. Recruitment thus prematurely stopped after only 38 patients were randomized (instead of the planned 112). The statistical plan has thus been adapted and we report the results in a descriptive, non-comparative manner. Nevertheless, our results consistently demonstrate that PFS, OS and response rates favor the combination of cediranib and gefitinib, without statistical significance. Increasing age and resection for recurrent disease were associated with significantly lower PFS. Advanced age is well recognised as a negative prognostic marker [25–27]. Surgical resection for recurrent disease has not been evaluated in a prospective clinical trial, and is considered standard practice only in patients with mass effect, thus patients requiring surgery may represent a patient group with more aggressive tumours [28]. However, patient numbers are small and the median progression-free and overall survival of the whole population were in the lower range of what has been reported in other negative clinical trials [20]. The trial establishes that the combination of drugs is tolerated in patients with glioblastoma. Whilst 89% of patients receiving cediranib plus gefitinib experienced serious toxicities (Grade 3 or 4) compared to 68% of those receiving cediranib plus placebo, no significant differences were observed in quality of life analyses.

The concept of utilizing rationally designed small molecule inhibitor targeted therapies in glioblastoma has largely not been successful and this raises key questions as to why this might be the case [29]. Are the drugs getting to the target? There is evidence that gefitinib crosses the blood brain barrier in brain metastases but there is also the question of drug concentration in the tumor [30]. Is the target present throughout the tumor [31]? The genetic heterogeneity observed in glioblastomas renders their growth less reliant on a solitary oncogene and hence there is an increasing acceptance of the need to appropriately combine targeted therapies [32]. Further to this, within an individual cancer cell, does resistance develop? A network of interconnected signal transduction pathways is responsible for the development and maintenance of many solid tumors. Indeed, parallel and reciprocal pathways exist between the VEGFR and EGFR signaling cascades, clearly linking these pathways within tumors [33]. Consequently, blockade of a single pathway may be ineffective in the long term because activation of other pathways can serve as escape mechanisms for the tumor [34]. Therefore, dual inhibition of VEGFR and EGFR signaling cascades are important for optimal suppression of tumor growth, as supported in this study, and have been shown to have synergistic effects in preclinical models [35].

EGFR, as well as being involved in controlling cell proliferation and apoptosis, has also been shown to play a role in tumor angiogenesis [36]. Activation of the EGFR pathway by either EGF or TGF increases the production of angiogenic factors (VEGF and VEGFR) in a variety of tumor cells including gastric, bladder, and pancreatic cell lines [37–39]. Preclinical studies using high grade glioma cell lines have similarly demonstrated that EGFR expression up-regulates the production of VEGF through mechanisms distinct from those under hypoxic regulation [40–42]. Conversely, inhibition of the EGFR pathway has been shown to reduce the production of angiogenic molecules in gastric, colonic, pancreatic and breast cancer cell lines [35]. Of particular relevance to this study, gefitinib treatment has been shown to cause a dose- and time-dependent decrease in VEGF production in in-vitro studies of various cancer cell lines [43].

Additional evidence that the EGFR and VEGFR pathways are linked has come from a study demonstrating that inhibition of the downstream EGFR-mediated effector, mammalian target of rapamycin (mTOR), reduces VEGF expression and capillary tube formation by endothelial cells [44]. Further support justifying the strategy of dual inhibition came from the discovery that combined inhibition of multiple targets has the potential to overcome resistance to monotherapies [45]. Van Cruijsen et al. demonstrated that resistance to anti-EGFR therapy could be overcome by adding antiangiogenic therapy to an anti-EGFR regimen [35]. Although some patients initially respond to EGFR TKIs, nearly all eventually acquire resistance to therapy following multiple or prolonged treatment [46]. Resistance can be acquired via several mechanisms. Mutation or over-activity of EGFR-independent signaling pathways, such as the acquisition of K-ras mutation, or loss of PTEN expression are often responsible. Conformational changes in the TKI binding domain, and ligand-independent activation may also occur. The induction of higher angiogenic potential via up-regulation of VEGF and other pro-angiogenic molecules by tumor cells has also been shown to be play a role [20, 47–49]. If this is the case, then dual inhibition of both pathways may act to prevent resistance to EGFR inhibition through VEGFR.

One resistance mechanism for cediranib is through up-regulation of epidermal growth factor receptor (EGFR). It is plausible that dual inhibition of VEGFR2 and EGFR, with cediranib and gefitinib respectively, may overcome this resistance and improve outcome [33, 50]. This study supports this approach, suggesting a trend towards an improved outcome with the combination of cediranib and gefitinib, and warrants further studies of dual EGFR and VEGF inhibition.

## Conclusion

Cediranib and gefitinib in combination is tolerated in patients with glioblastoma. Incomplete recruitment led to the study being underpowered. However, a trend towards improved survival and response rates with the addition of gefitinib to cediranib was observed. Further studies of the combination incorporating EGFR and VEGF inhibition are warranted.

## Supporting Information

S1 CONSORT Checklist CONSORT checklist. (DOC)

S1 Protocol Protocol. (PDF)

## References

[1] Brodbelt A, Greenberg D, Winters T, Williams M, Vernon S, Collins VP, et al Glioblastoma in England: 2007–2011. Eur J Cancer. 2015;51(4):533–42. 10.1016/j.ejca.2014.12.014 . 25661102

[2] Ostrom QT, Gittleman H, Fulop J, Liu M, Blanda R, Kromer C, et al CBTRUS Statistical Report: Primary Brain and Central Nervous System Tumors Diagnosed in the United States in 2008–2012. Neuro-oncology. 2015;17 Suppl 4:iv1–iv62. 10.1093/neuonc/nov189 . 26511214 PMC4623240

[3] Stupp R, Mason WP, van den Bent MJ, Weller M, Fisher B, Taphoorn MJ, et al Radiotherapy plus concomitant and adjuvant temozolomide for glioblastoma. The New England journal of medicine. 2005;352(10):987–96. 10.1056/NEJMoa043330 . 15758009

[4] Stupp R, Hegi ME, Mason WP, van den Bent MJ, Taphoorn MJ, Janzer RC, et al Effects of radiotherapy with concomitant and adjuvant temozolomide versus radiotherapy alone on survival in glioblastoma in a randomised phase III study: 5-year analysis of the EORTC-NCIC trial. The Lancet Oncology. 2009;10(5):459–66. 10.1016/S1470-2045(09)70025-7 . 19269895

[5] Nicholson RI, Gee JM, Harper ME. EGFR and cancer prognosis. Eur J Cancer. 2001;37 Suppl 4:S9–15. . 11597399 10.1016/s0959-8049(01)00231-3

[6] Libermann TA, Nusbaum HR, Razon N, Kris R, Lax I, Soreq H, et al Amplification, enhanced expression and possible rearrangement of EGF receptor gene in primary human brain tumours of glial origin. Nature. 1985;313(5998):144–7. . 2981413 10.1038/313144a0

[7] Wong AJ, Bigner SH, Bigner DD, Kinzler KW, Hamilton SR, Vogelstein B. Increased expression of the epidermal growth factor receptor gene in malignant gliomas is invariably associated with gene amplification. Proceedings of the National Academy of Sciences of the United States of America. 1987;84(19):6899–903. 3477813 10.1073/pnas.84.19.6899 PMC299192

[8] Ekstrand AJ, James CD, Cavenee WK, Seliger B, Pettersson RF, Collins VP. Genes for epidermal growth factor receptor, transforming growth factor alpha, and epidermal growth factor and their expression in human gliomas in vivo. Cancer research. 1991;51(8):2164–72. . 2009534

[9] Frederick L, Wang XY, Eley G, James CD. Diversity and frequency of epidermal growth factor receptor mutations in human glioblastomas. Cancer research. 2000;60(5):1383–7. . 10728703

[10] Pelloski CE, Ballman KV, Furth AF, Zhang L, Lin E, Sulman EP, et al Epidermal growth factor receptor variant III status defines clinically distinct subtypes of glioblastoma. Journal of clinical oncology: official journal of the American Society of Clinical Oncology. 2007;25(16):2288–94. 10.1200/JCO.2006.08.0705 . 17538175

[11] Wong AJ, Ruppert JM, Bigner SH, Grzeschik CH, Humphrey PA, Bigner DS, et al Structural alterations of the epidermal growth factor receptor gene in human gliomas. Proceedings of the National Academy of Sciences of the United States of America. 1992;89(7):2965–9. 1557402 10.1073/pnas.89.7.2965 PMC48784

[12] Salmaggi A, Eoli M, Frigerio S, Silvani A, Gelati M, Corsini E, et al Intracavitary VEGF, bFGF, IL-8, IL-12 levels in primary and recurrent malignant glioma. Journal of neuro-oncology. 2003;62(3):297–303. . 12777082 10.1023/a:1023367223575

[13] Rong Y, Durden DL, Van Meir EG, Brat DJ. 'Pseudopalisading' necrosis in glioblastoma: a familiar morphologic feature that links vascular pathology, hypoxia, and angiogenesis. Journal of neuropathology and experimental neurology. 2006;65(6):529–39. . 16783163 10.1097/00005072-200606000-00001

[14] Rong Y, Hu F, Huang R, Mackman N, Horowitz JM, Jensen RL, et al Early growth response gene-1 regulates hypoxia-induced expression of tissue factor in glioblastoma multiforme through hypoxia-inducible factor-1-independent mechanisms. Cancer research. 2006;66(14):7067–74. 10.1158/0008-5472.CAN-06-0346 16849552 PMC2610484

[15] Jain RK, di Tomaso E, Duda DG, Loeffler JS, Sorensen AG, Batchelor TT. Angiogenesis in brain tumours. Nature reviews Neuroscience. 2007;8(8):610–22. 10.1038/nrn2175 . 17643088

[16] Vredenburgh JJ, Desjardins A, Herndon JE 2nd, Marcello J, Reardon DA, Quinn JA, et al Bevacizumab plus irinotecan in recurrent glioblastoma multiforme. Journal of clinical oncology: official journal of the American Society of Clinical Oncology. 2007;25(30):4722–9. 10.1200/JCO.2007.12.2440 . 17947719

[17] Friedman HS, Prados MD, Wen PY, Mikkelsen T, Schiff D, Abrey LE, et al Bevacizumab alone and in combination with irinotecan in recurrent glioblastoma. Journal of clinical oncology: official journal of the American Society of Clinical Oncology. 2009;27(28):4733–40. 10.1200/JCO.2008.19.8721 . 19720927

[18] Batchelor TT, Reardon DA, de Groot JF, Wick W, Weller M. Antiangiogenic therapy for glioblastoma: current status and future prospects. Clinical cancer research: an official journal of the American Association for Cancer Research. 2014;20(22):5612–9. 10.1158/1078-0432.CCR-14-0834 25398844 PMC4234180

[19] Wedge SR, Kendrew J, Hennequin LF, Valentine PJ, Barry ST, Brave SR, et al AZD2171: a highly potent, orally bioavailable, vascular endothelial growth factor receptor-2 tyrosine kinase inhibitor for the treatment of cancer. Cancer research. 2005;65(10):4389–400. 10.1158/0008-5472.CAN-04-4409 . 15899831

[20] Batchelor TT, Mulholland P, Neyns B, Nabors LB, Campone M, Wick A, et al Phase III randomized trial comparing the efficacy of cediranib as monotherapy, and in combination with lomustine, versus lomustine alone in patients with recurrent glioblastoma. Journal of clinical oncology: official journal of the American Society of Clinical Oncology. 2013;31(26):3212–8. 10.1200/JCO.2012.47.2464 23940216 PMC4021043

[21] Rich JN, Reardon DA, Peery T, Dowell JM, Quinn JA, Penne KL, et al Phase II trial of gefitinib in recurrent glioblastoma. Journal of clinical oncology: official journal of the American Society of Clinical Oncology. 2004;22(1):133–42. 10.1200/JCO.2004.08.110 . 14638850

[22] Franceschi E, Cavallo G, Lonardi S, Magrini E, Tosoni A, Grosso D, et al Gefitinib in patients with progressive high-grade gliomas: a multicentre phase II study by Gruppo Italiano Cooperativo di Neuro-Oncologia (GICNO). British journal of cancer. 2007;96(7):1047–51. 10.1038/sj.bjc.6603669 17353924 PMC2360116

[23] van Cruijsen H, Voest EE, Punt CJ, Hoekman K, Witteveen PO, Meijerink MR, et al Phase I evaluation of cediranib, a selective VEGFR signalling inhibitor, in combination with gefitinib in patients with advanced tumours. Eur J Cancer. 2010;46(5):901–11. 10.1016/j.ejca.2009.12.023 . 20061136

[24] Wen PY, Macdonald DR, Reardon DA, Cloughesy TF, Sorensen AG, Galanis E, et al Updated response assessment criteria for high-grade gliomas: response assessment in neuro-oncology working group. Journal of clinical oncology: official journal of the American Society of Clinical Oncology. 2010;28(11):1963–72. 10.1200/JCO.2009.26.3541 . 20231676

[25] Lamborn KR, Chang SM, Prados MD. Prognostic factors for survival of patients with glioblastoma: recursive partitioning analysis. Neuro-oncology. 2004;6(3):227–35. Epub 07/29. 10.1215/s1152851703000620 . 15279715 PMC1871999

[26] Carson KA, Grossman SA, Fisher JD, Shaw EG. Prognostic factors for survival in adult patients with recurrent glioma enrolled onto the new approaches to brain tumor therapy CNS consortium phase I and II clinical trials. Journal of clinical oncology: official journal of the American Society of Clinical Oncology. 2007;25(18):2601–6. Epub 06/20. 10.1200/jco.2006.08.1661 . 17577040 PMC4118746

[27] Gorlia T, van den Bent MJ, Hegi ME, Mirimanoff RO, Weller M, Cairncross JG, et al Nomograms for predicting survival of patients with newly diagnosed glioblastoma: prognostic factor analysis of EORTC and NCIC trial 26981-22981/CE.3. The Lancet Oncology. 2007;9(1):29–38. Epub 12/18. 10.1016/s1470-2045(07)70384-4 . 18082451

[28] Brandes AA, Bartolotti M, Franceschi E. Second surgery for recurrent glioblastoma: advantages and pitfalls. Expert review of anticancer therapy. 2013;13(5):583–7. Epub 04/27. 23617349 10.1586/era.13.32

[29] Mulholland PJ, Thirlwell C, Brock CS, Newlands ES. Emerging targeted treatments for malignant glioma. Expert opinion on emerging drugs. 2005;10(4):845–54. 10.1517/14728214.10.4.845 . 16262566

[30] Cappuzzo F, Ardizzoni A, Soto-Parra H, Gridelli C, Maione P, Tiseo M, et al Epidermal growth factor receptor targeted therapy by ZD 1839 (Iressa) in patients with brain metastases from non-small cell lung cancer (NSCLC). Lung cancer. 2003;41(2):227–31. . 12871787 10.1016/s0169-5002(03)00189-2

[31] Forshew T, Lewis P, Waldman A, Peterson D, Glaser M, Brock C, et al Three different brain tumours evolving from a common origin. Oncogenesis. 2013;2:e41 10.1038/oncsis.2013.1 23545860 PMC3641358

[32] Pillay V, Allaf L, Wilding AL, Donoghue JF, Court NW, Greenall SA, et al The plasticity of oncogene addiction: implications for targeted therapies directed to receptor tyrosine kinases. Neoplasia. 2009;11(5):448–58, 2 p following 58. 19412429 10.1593/neo.09230 PMC2671857

[33] Tabernero J. The role of VEGF and EGFR inhibition: implications for combining anti-VEGF and anti-EGFR agents. Molecular cancer research: MCR. 2007;5(3):203–20. 10.1158/1541-7786.MCR-06-0404 . 17374728

[34] Scagliotti GV. Potential role of multi-targeted tyrosine kinase inhibitors in non-small-cell lung cancer. Annals of oncology: official journal of the European Society for Medical Oncology / ESMO. 2007;18 Suppl 10:x32–41. 10.1093/annonc/mdm412 . 17761722

[35] van Cruijsen H, Giaccone G, Hoekman K. Epidermal growth factor receptor and angiogenesis: Opportunities for combined anticancer strategies. International journal of cancer Journal international du cancer. 2005;117(6):883–8. 10.1002/ijc.21479 . 16152621

[36] Ellis LM. Epidermal growth factor receptor in tumor angiogenesis. Hematology/oncology clinics of North America. 2004;18(5):1007–21, viii 10.1016/j.hoc.2004.06.002 . 15474332

[37] Akagi M, Kawaguchi M, Liu W, McCarty MF, Takeda A, Fan F, et al Induction of neuropilin-1 and vascular endothelial growth factor by epidermal growth factor in human gastric cancer cells. British journal of cancer. 2003;88(5):796–802. 10.1038/sj.bjc.6600811 12618892 PMC2376351

[38] Perrotte P, Matsumoto T, Inoue K, Kuniyasu H, Eve BY, Hicklin DJ, et al Anti-epidermal growth factor receptor antibody C225 inhibits angiogenesis in human transitional cell carcinoma growing orthotopically in nude mice. Clinical cancer research: an official journal of the American Association for Cancer Research. 1999;5(2):257–65. . 10037173

[39] Parikh AA, Liu WB, Fan F, Stoeltzing O, Reinmuth N, Bruns CJ, et al Expression and regulation of the novel vascular endothelial growth factor receptor neuropilin-1 by epidermal growth factor in human pancreatic carcinoma. Cancer. 2003;98(4):720–9. 10.1002/cncr.11560 . 12910515

[40] Maity A, Pore N, Lee J, Solomon D, O'Rourke DM. Epidermal growth factor receptor transcriptionally up-regulates vascular endothelial growth factor expression in human glioblastoma cells via a pathway involving phosphatidylinositol 3'-kinase and distinct from that induced by hypoxia. Cancer research. 2000;60(20):5879–86. . 11059786

[41] Clarke K, Smith K, Gullick WJ, Harris AL. Mutant epidermal growth factor receptor enhances induction of vascular endothelial growth factor by hypoxia and insulin-like growth factor-1 via a PI3 kinase dependent pathway. British journal of cancer. 2001;84(10):1322–9. 10.1054/bjoc.2001.1805 11355942 PMC2363647

[42] Pore N, Liu S, Haas-Kogan DA, O'Rourke DM, Maity A. PTEN mutation and epidermal growth factor receptor activation regulate vascular endothelial growth factor (VEGF) mRNA expression in human glioblastoma cells by transactivating the proximal VEGF promoter. Cancer research. 2003;63(1):236–41. . 12517803

[43] Ciardiello F, Caputo R, Bianco R, Damiano V, Fontanini G, Cuccato S, et al Inhibition of growth factor production and angiogenesis in human cancer cells by ZD1839 (Iressa), a selective epidermal growth factor receptor tyrosine kinase inhibitor. Clinical cancer research: an official journal of the American Association for Cancer Research. 2001;7(5):1459–65. . 11350918

[44] Bianco R, Garofalo S, Rosa R, Damiano V, Gelardi T, Daniele G, et al Inhibition of mTOR pathway by everolimus cooperates with EGFR inhibitors in human tumours sensitive and resistant to anti-EGFR drugs. British journal of cancer. 2008;98(5):923–30. 10.1038/sj.bjc.6604269 18319715 PMC2266842

[45] Rubin BP, Duensing A. Mechanisms of resistance to small molecule kinase inhibition in the treatment of solid tumors. Laboratory investigation; a journal of technical methods and pathology. 2006;86(10):981–6. 10.1038/labinvest.3700466 . 16924245

[46] Camp ER, Summy J, Bauer TW, Liu W, Gallick GE, Ellis LM. Molecular mechanisms of resistance to therapies targeting the epidermal growth factor receptor. Clinical cancer research: an official journal of the American Association for Cancer Research. 2005;11(1):397–405. . 15671571

[47] Viloria-Petit A, Crombet T, Jothy S, Hicklin D, Bohlen P, Schlaeppi JM, et al Acquired resistance to the antitumor effect of epidermal growth factor receptor-blocking antibodies in vivo: a role for altered tumor angiogenesis. Cancer research. 2001;61(13):5090–101. . 11431346

[48] Ciardiello F, Bianco R, Caputo R, Caputo R, Damiano V, Troiani T, et al Antitumor activity of ZD6474, a vascular endothelial growth factor receptor tyrosine kinase inhibitor, in human cancer cells with acquired resistance to antiepidermal growth factor receptor therapy. Clinical cancer research: an official journal of the American Association for Cancer Research. 2004;10(2):784–93. . 14760102 10.1158/1078-0432.ccr-1100-03

[49] Naumov GN, Nilsson MB, Cascone T, Briggs A, Straume O, Akslen LA, et al Combined vascular endothelial growth factor receptor and epidermal growth factor receptor (EGFR) blockade inhibits tumor growth in xenograft models of EGFR inhibitor resistance. Clinical cancer research: an official journal of the American Association for Cancer Research. 2009;15(10):3484–94. 10.1158/1078-0432.CCR-08-2904 19447865 PMC2893040

[50] Ciardiello F, Troiani T, Bianco R, Orditura M, Morgillo F, Martinelli E, et al Interaction between the epidermal growth factor receptor (EGFR) and the vascular endothelial growth factor (VEGF) pathways: a rational approach for multi-target anticancer therapy. Annals of oncology: official journal of the European Society for Medical Oncology / ESMO. 2006;17 Suppl 7:vii109–14. 10.1093/annonc/mdl962 . 16760272

