# Supplementary material for: Multi-Center Randomized Phase II Study Comparing Cediranib plus Gefitinib with Cediranib plus Placebo in Subjects with Recurrent/Progressive Glioblastoma
Source: PLoS One. 2016 May 27;11(5):e0156369. doi: 10.1371/journal.pone.0156369 (PMC4883746; doi:10.1371/journal.pone.0156369)
Supplement: S1 Protocol — (PDF) [file pone.0156369.s002.pdf]

DORIC

Multi-centre, randomised, double-blind phase II study comparing cediranib (AZD2171) plus gefitinib (Iressa, ZD1839) with cediranib plus placebo in subjects with recurrent/progressive glioblastoma

**Trial Sponsor:**	University College London
**Trial Sponsor reference:**	UCL/10/0035
**Trial funder:**	AstraZeneca, endorsed by CR UK
**Funder reference:**	ISSRECE00002<br>CRUKE/10/044
**ISRCTN/Clinicaltrials.gov no:**	[pending]
**EUDRACT no:**	2010-021531-13
**CTA no:**	29780/0003/001
**Protocol version no:**	**1.0**
**Protocol version date:**	**30.11.2010**

Authorisation signatures

**Name & Role:**	**Signature:**	**Date authorised:**
Chief Investigator:<br>Dr Paul Mulholland<br>Consultant in medical oncology	Paul Mulholland	30th Nov 2010
For the Sponsor:<br>Professor Jonathan Ledermann<br>Director, UCL CTC	J. A. Ledermann	1st December 2010

Coordinating Centre:

For general queries, supply of trial documentation and central data management please contact:

DORIC Trial Coordinator
Cancer Research UK & UCL Cancer Trials Centre
90 Tottenham Court Road
London
W1T 4TJ

Tel: +44 (0) 20 7679 9860
Fax: +44 (0) 20 7679 9861
09:00 to 17:00 Monday to Friday (UK time)
Email: doric@ctc.ucl.ac.uk

Other trial contacts:

Chief Investigator: Dr Paul Mulholland PhD, MRCP
Address: Consultant in medical oncology
University College London Hospitals
Department of Oncology
1st Floor Central
250 Euston Road
London, UK
NW1 2PG
Phone +44 207 3809358
Fax +44 207 3809055

Trial Management Group (TMG):

Ifty Khan	Statistician	CR UK & UCL Cancer Trials Centre
Ian Tomlinson	Co-investigator	University of Oxford
Roger Stupp	Co-investigator	University of Lausanne, Switzerland
Catherine Mcbain	Co-investigator	Christie Hospital, Manchester
Cathryn Brock	Co-investigator	Charing Cross Hospital, London
Sarah Jefferies	Co-investigator	Addenbrookes Hospital, Cambridge
Paul Smith	Tumour Group Lead	CR UK & UCL Cancer Trials Centre
Chaya Patel	Research nurse	University College London Hospital
Nada Mansour	Pharmacist	University College London Hospital
Hans Rolf Jäger	Consultant Neuroradiologist	University College London Hospital
Harpreet Hyare	Consultant Neuroradiologist	University College London Hospital

The trial funder is supporting central coordination through the Cancer Research UK & UCL Cancer Trials Centre (UCL CTC). Problems relating to this trial should be referred, in the first instance, to UCL CTC.

This trial will adhere to the principles of ICH Harmonised Tripartite Guideline for Good Clinical Practice (CPMP/ICH/135/95) as set out in Schedule 1 of the Medicines for Human Use (Clinical Trials) Regulations 2004 and the GCP Directive 2005/28/EC, and any amendments thereto. It will be conducted in compliance with the protocol, the Data Protection Act 1998 (Z6364106/2008/8/14), the Medicines for Human Use (clinical trials) regulations 2004, as amended from time to time, and other regulatory requirements as appropriate.

Table of Contents

1.0 Protocol Summary 6
1.1 Summary of Trial Design 6
1.2 Trial Schema 7

2.0 Introduction 8
2.1 Background 8
2.1.1 Glioblastoma 8
2.1.2 Glioblastoma genetics 9
2.2 The rationale for therapeutic targeting 9
2.2.1 Rationale for targeting VEGF pathway 9
2.2.2 Rationale for targeting EGFR 10
2.2.3 Rationale for targeting both the VEGF and EGFR pathways 11
2.3 Agents in the trial 12
2.3.1 Cediranib 12
2.3.2 Gefitinib (Iressa, ZD1839) 13
2.4 Clinical trial experience 14
2.4.1 Cediranib 14
2.4.1.1 Phase III study of cediranib versus lomustine versus cediranib plus lomustine in glioblastoma 14
2.4.1.2 Trials using cediranib in other cancers 15
2.4.2 Gefitinib 16
2.4.3 Trials targeting EGFR and VEGFR 17
2.4.3.1 Phase I study of cediranib and gefitinib 19
2.4.3.2 Trials of VEGFR and EGFR combination therapy in glioblastoma 20
2.5 Proposed Trial 21
2.5.1 Primary Objective 22
2.5.2 Secondary Objectives 22
2.6 Outcome variables 22
2.6.1 Efficacy 22
2.6.1.1 Primary outcome variable 22
2.6.1.2 Secondary outcome variables 24
2.6.2 Patient reported outcomes (PROs) 25
2.6.3 Safety 25
2.6.4 Pharmacodynamic and Pharmacogenetic 25
2.7 Trial activation 26

3.0 Selection of Sites/Site Investigators 27
3.1 Site selection 27
3.1.1 Selection of Principal Investigator and other investigators at sites 27
3.2 Site initiation and activation 27
3.2.1 Site initiation 27
3.2.2 Site activation 28

4.0 Informed consent 29

5.0 Selection of Patients 30
5.1 Patient Eligibility 30
5.1.1 Inclusion Criteria 30
5.1.2 Exclusion Criteria 31
5.1.3 Pregnancy and Birth Control 34
5.2 Pre-randomisation evaluation 35
5.3 Screening Log 36

6.0 Randomisation procedures 37
6.1 Randomisation 37
6.2 Trial drug supply 38
6.2.1 Initial trial drug supply 38
6.2.2 Resupply of cediranib & gefitinib/placebo 39

7.0 Trial Treatment 40
7.1 Treatment summary 40
7.2 Summary Treatment Schedule 40
7.3 Trial Treatment Details 41
7.3.1 Treatment compliance 43
7.3.1.1 Difficulties swallowing tablets 43
7.3.2 Pharmacy responsibilities: 44
7.3.3 Drug accountability 46
7.3.4 Dose modifications 46
7.4 Management of general toxicity 47
7.4.1 Management of toxicity attributable to cediranib 47
7.4.1.1 Management of proteinuria 49
7.4.1.2 Management of abnormal thyroid function tests 49
7.4.1.3 Management of haematological toxicity 50
7.4.1.4 Hypertension management 50
7.4.1.5 Dermatological toxicity 53
7.4.1.6 Oral toxicity 54
7.4.1.7 Reversible posterior leukoencephalopathy syndrome 54
7.4.1.8 Fatigue 55
7.4.2 Management of toxicity attributable to gefitinib 56
7.4.2.1 Skin toxicity 57
7.4.2.2 Interstitial Lung Disease 58
7.4.3 Diarrhoea 59
7.4.3.1 Initial management of diarrhoea 59
7.4.3.2 Management of persistent (>24h) or uncontrolled severe diarrhoea despite loperamide 60
7.4.3.3 Management of persistent (>48h) diarrhoea despite loperamide 60
7.4.3.4 Dose modifications for cediranib and gefitinib for diarrhoea 60
7.4.4 Renal Impairment 62
7.5 Concomitant medication 62
7.6 Unblinding 63
7.6.1 Methods for ensuring blinding 63
7.6.2 Methods for unblinding 64
7.7 Other trial treatments/interventions: Imaging 66

8.0 Assessments 69
8.1 Schedule of Assessments 69
8.2 Schedule of Assessments – further details 71
8.3 Patient diaries 73
8.4 Blood pressure monitors 74

9.0 Data Management Guidelines 75
9.1 Completing Case Report Forms 75
9.2 Corrections to CRFs 75
9.3 Missing Data 75
9.4 Data Queries 75
9.5 Timelines for data return 76

10.0 Pharmacovigilance 77
10.1 Definitions of Adverse Events 77
10.2 Reporting Procedures 78
10.2.1 All Adverse Events (AEs) 78
10.2.1.1 Overdoses 78
10.2.1.2 Adverse Event Term 78
10.2.1.3 Severity 78
10.2.1.4 Causality 79
10.2.2 Serious Adverse Events (SAEs) 80
10.2.2.1 Events which do not require immediate reporting on an SAE Report 80
10.2.2.3 Adverse Event Reporting Flowchart 81
10.2.2.4 SAE Follow-Up Reports 82
10.2.2.5 SAE Processing at UCL CTC 82
10.2.2.6 Emergency unblinding of the treatment allocation 82
10.3 SUSARs 82
10.3.1 Informing Sites of SUSARs 83
10.4 Clinical Review 83
10.5 Additional Safety Monitoring at UCL CTC 83
10.6 Pregnancy 83
10.6.1 Pregnancy Follow-Up Reports 84
10.6.2 SAEs During Pregnancy 84
10.6.3 Pregnancy Report Processing at UCL CTC 84
10.7 Annual Safety Reports 85

11.0 Incident Reporting and Serious Breaches 85
11.1 Incident Reporting 85
11.2 Serious Breaches 85

12.0 Trial Monitoring and oversight 86
12.1 On-site monitoring 86
12.2 Self-assessment monitoring 87
12.3 Central monitoring 87
12.4 Non-Compliance/'for cause' on-site monitoring 87
12.5 Oversight Committees 88
12.5.1 Trial Management Group (TMG) 88
12.5.2 Independent Trial Steering Committee (ITSC) 88
12.5.3 Independent Data Monitoring Committee (IDMC) 88
12.5.4 Role of UCL CTC 89

13 Withdrawal of patients 90
13.1 Withdrawal from Trial Treatment 90
13.1.1 Discontinuation of Trial Drugs 90
13.1.2 Procedures for discontinuation from treatment 91
13.2 Withdrawal of Consent to Data Collection 92
13.3 Losses to follow-up 92
13.4 Withdrawal from Genetic Research 93
13.4.1 Procedures for discontinuation from genetic aspects of the study 93

14.0 Trial Closure 95
14.1 End of Trial 95
14.2 Archiving of Trial Documentation 95
14.3 Early discontinuation of trial 95
14.4 Withdrawal from trial participation by a site 96

15.0 Quality Assurance 97

16.0 Statistics 98
16.1 Sample size calculation 98
16.2 Population for analysis 99
16.3 Statistical Analysis of the primary endpoint 99
16.4 Statistical Analysis of the Secondary Endpoints 100
16.5 Statistical Analysis of Safety Data 103
16.6 Statistical Analysis of other endpoints 104
16.7 Handling missing data 104
16.8 Interim Analysis 104

17.0 Ethical and Regulatory Approvals 105
17.1 Ethical Approval 105
17.2 Regulatory Approval 106
17.3 Local Site Approval 106
17.4 Protocol Amendments 106
17.5 Patient Confidentiality & Data Protection 106

18.0 Sponsorship and Indemnity 107
18.1 Sponsor Details 107
18.2 Indemnity 107

19.0 Funding 108

20.0 Publication Policy 109

21.0 Translational Research 110

22.0 References 112

Appendix 1: Abbreviations 118
Appendix 2: Recommended Skin Care products 120
Appendix 3: Recommended Oral Care products 121
Appendix 4: Drugs thought to significantly interact with CYP450 enzymes 122
Appendix 5: Performance Status Evaluation 123
Appendix 6: Quality of Life Assessments 124
Appendix 7: QTc with Bazetts Correction 127
Appendix 8: MRI Acquisition form 128
Appendix 9: Protocol Version History 129

1.0 Protocol Summary

1.1 Summary of Trial Design

**Title:**	Multi-centre, randomised, double-blind phase II study comparing cediranib (AZD2171) plus gefitinib (Iressa, ZD1839) with cediranib plus placebo in subjects with recurrent/progressive glioblastoma
**Short Title/acronym:**	DORIC
**EUDRACT no:**	2010-021531-13
**Sponsor name & reference:**	University College London, UCL/10/0035
**Funder name & reference:**	AstraZeneca, endorsed by CR-UK
**Clinicaltrials.gov no:**	**(To Be Confirmed)**
**Design:**	Multi-centre, randomised, double-blind phase II study
**Overall aim:**	The primary objective of the study is to determine the efficacy of cediranib in combination with oral gefitinib and cediranib alone by assessment of progression free survival (PFS) defined as the time from randomisation to first progression or death (whichever occurs first)
**Primary endpoint:**	Progression free survival (PFS) defined as the time from the date of randomisation to the date of first progression or death due to any cause, whichever one comes first.
**Secondary endpoints:**	• Overall survival (OS)<br>• Radiographic response rate (RR)<br>• PFS rate at 6 months (24 weeks)<br>• 12 months survival rate<br>• Steroid use<br>• Time to deterioration of neurological status<br>• Safety and tolerability
**Target accrual:**	112 patients
**Planned number of sites:**	6 centres
**Target countries:**	UK
**Treatment summary:**	Enrolled subjects will be randomised to receive **either** cediranib 30mg od orally and gefitinib 500mg od orally **or** cediranib 30mg od plus placebo.<br>Each cycle of treatment lasts 6 weeks. Patients will be treated until progression (unless clinician feels patient is receiving benefit in which case treatment can continue), patient decision or the development of unacceptable toxicity.
**Anticipated duration of recruitment:**	18 months
**Duration of patient follow up:**	All patients will be followed up until death
**Definition of end of trial:**	The end of trial declaration will be sent 6 months following last patient finishing treatment, or when last patient has died, whichever is sooner. Follow up will be continued until the last patient has died.
**Translational component:**	Potential biomarker roles of EGFR, EGFR vIII, PTEN, methylation of MGMT, IDH 1 and 2 will be assessed.
**Other related research:**	Exploratory work with genetic and expression profiling will be carried out on post-mortem tumour samples donated where consent is given for this.

1.2 Trial Schema

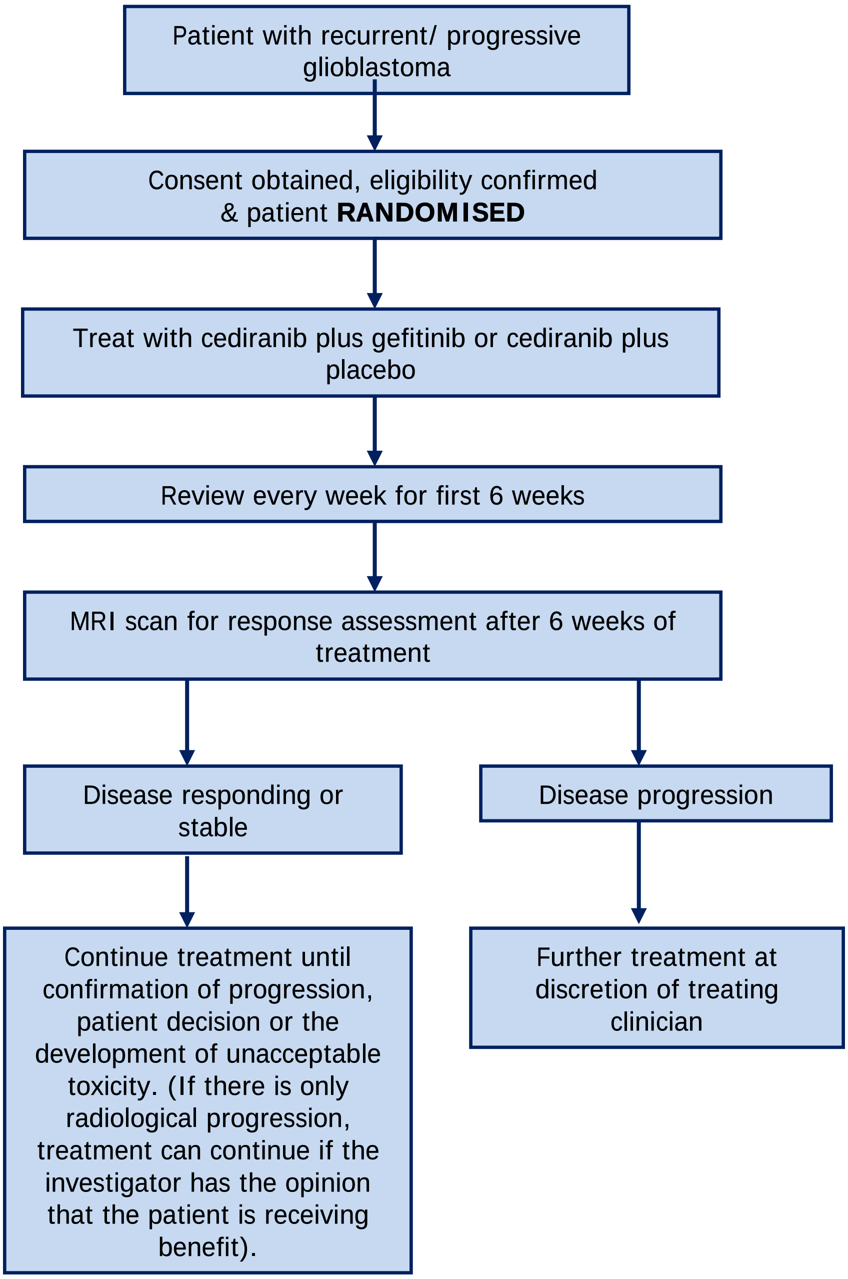

2.0 Introduction

2.1 Background

2.1.1 Glioblastoma

There are approximately 3,500 new cases of malignant glioma diagnosed per year in England and Wales (Office for National Statistics, Cancer Registration Statistics, series MB1 no.37, 2006. http://www.statistics.gov.uk). Gliomas account for 50-60% of all primary brain tumours, and occur at an incidence of 3-4 per 100,000 population; glioblastoma (WHO grade IV) accounts for around 40-50% of gliomas, with an annual incidence of 1.2-2 per 100,000 population (Dinnes et al. 2000). Glioblastoma is a highly infiltrative, rapidly progressive primary brain tumour that continues to be associated with a poor prognosis with most patients dying within a year of diagnosis.

The initial treatment of glioblastoma consists of surgical resection where possible. This is followed by radiotherapy with concurrent temozolomide chemotherapy followed by 6 months of maintenance temozolomide chemotherapy (Stupp et al. 2005). The addition of temozolomide to radiotherapy led to an increase in survival, in a population in whom outcomes from treatment had not improved for three decades with median survival in the temozolomide and radiotherapy versus radiotherapy alone of 14.6 months (95% CI 13.2-16.8) versus 12.1 months (11.2-13.0) and five-year survival of 9·8% (95% CI 6·4–14·0) versus 1·9% (0·6–4·4) (Stupp et al. 2009; Stupp et al. 2005).

Disease relapse invariably occurs during or after standard first-line treatment. No standard treatment exists for the management of patients with recurrent glioblastoma and the prognosis associated with relapsed disease remains poor. Patients with relapsed disease are traditionally offered nitrosoureabased chemotherapy or investigational therapies. Historically these have been associated with objective tumour response rates of less than 10% and less than 20% of patients are alive and progression free at 6 months, with a median progression free survival of 2 months (Wong et al. 1999; Yung et al. 2000). This is supported by more recent data from a phase III study comparing efficacy and safety of enzastaurin versus lomustine (Wick et al. 2010a). This trial was terminated at a planned interim analysis due to non-superior results for enzastaurin. The results were for enzastaurin and lomustine, respectively, median PFS (1.5 v 1.6 months; HR = 1.28; 95% CI, 0.97 to 1.70), overall survival (6.6 v 7.1 months; HR = 1.20; 95% CI, 0.88 to 1.65) (Wick et al. 2010a). Consequently there remains a substantial unmet need to develop and implement novel treatment approaches to improve morbidity and mortality in this patient group.

2.1.2 Glioblastoma genetics

Glioblastomas have subdivided into 'primary' and 'secondary' glioblastoma (Ohgaki and Kleihues 2007). The patients with primary glioblastoma are generally older with activating mutations in EGFR. The secondary glioblastoma patients are generally younger and have tumours containing p53 mutation, which have developed from lower grade tumours. More recently this classification has been expanded based on genomic and expression changes with "classical" being defined by aberrations in EGFR, "mesenchymal" by NF1, "proneuronal" by changes in PDGFRA and IDH1 and a "neural" group defined by neural markers (Verhaak et al. 2010; Yan et al. 2009). Co-amplification of PDGFRA, KIT and VEGFR (KDR) occur in 15% of glioblastoma and have been cited as targets for tyrosine kinase inhibitor therapy (Holtkamp et al. 2007).

Differential response to therapy has been demonstrated depending on the methylation status of the MGMT promotor for both radiotherapy and temozolomide (Hegi et al. 2005). In addition, EGFR mutation with an intact PTEN has been suggested as a predictor of response to small molecule EGFR inhibitors (Mellinghoff et al. 2005). However despite an improved understanding of the molecular aberrations that occur in these tumours the use of molecularly targeted therapies have been disappointing (Grossman et al. 2010; Wick et al. 2010a; Wick et al. 2010b).

In this investigational study we propose the use of a combination of therapies targeting the vascular endothelial growth factor (VEGF) (cediranib) and the epidermal growth factor receptor (EGFR) (gefitinib) pathways in patients with recurrent glioblastoma, a group of patients with a clearly unmet clinical need. The combination of cediranib and gefitinib has been explored in a phase I study in solid tumours (van Cruijsen et al. 2010).

2.2 The rationale for therapeutic targeting

2.2.1 Rationale for targeting VEGF pathway

Angiogenesis is the process through which new blood vessels are formed. Glioblastomas are profusely vascular tumours featuring prolific levels of angiogenesis and this is in part attributable to their high levels of vascular endothelial growth factor (VEGF) expression (Salmaggi et al. 2003). Pathologically glioblastomas are distinguished from low-grade gliomas by microvascular hyperplasia and focal necrosis. The aberrant vasculature that characterises high-grade gliomas is induced by a hypoxic microenvironment which in turn drives the expression of hypoxia inducible factor (HIF-1). This leads to the secretion of pro-angiogenesis factors (including VEGF and IL-8). Hypoxia also drives the local

expression of tissue factor which initiates pro-thrombotic conditions and these in turn contribute to the anomalous processes of neo-vascularisation that define glioblastomas (Rong et al. 2006a; Rong et al. 2006b). Disrupting the processes involved in angiogenesis is being increasingly adopted as a key element of the management of many tumour types including glioblastoma (Jain et al. 2007).

The use of anti-angiogenesis agents ‘normalises’ tumour vasculature, reducing tumour hypoxia, correcting interstitial hypertension and oedema. Each of these processes is thought to be desirable in enabling the more effective delivery of therapeutic agents (Batchelor et al. 2007). By reducing interstitial hypertension and oedema ‘anti-VEGF’ treatments have demonstrated pronounced ‘steroid sparing’ effects. In the context of recent phase II trials the use of the monoclonal antibody bevacizumab on its own or in combination with chemotherapeutic agents has resulted in unprecedented levels of radiographic response in patients with recurrent glioblastoma, resulting in FDA approval for use in recurrent glioblastoma (Cohen et al. 2009b; Friedman et al. 2009; Vredenburgh et al. 2007). Bevacizumab has EMEA approval for use in some cancers but not glioblastoma (EMEA/CHMP/746208/2009).

In health the blood-brain barrier effectively limits the delivery of therapeutic drugs to the central nervous system. The blood-brain barrier (BBB) is formed by the interactions of the specialised endothelial cells of the cerebral microvasculature and the end-foot processes of astrocytes. It is essential in maintaining homeostasis within the central nervous system. It acts as a selective barrier to the diffusion of molecules on the basis of their size and lipid solubility. In general macro-molecular drugs are excluded from crossing the blood-brain barrier whilst small, lipid soluble agents can cross the barrier passively and small hydrophilic substances require active transport mechanisms. Targeted therapies currently include both monoclonal antibodies and small molecule inhibitors. Monoclonal antibodies are complex proteins measuring 150,000 Daltons in size whilst small molecule inhibitors are of the order of 400 Daltons (Esteva 2004). Small molecule targeted therapies have been demonstrated to cross the blood brain barrier in-vivo unlike monoclonal antibodies which are readily excluded by virtue of their size. The development of malignant lesions within the brain (primary and secondary) perturbs the function of the blood-brain barrier as is evidenced by the sequelae of vasogenic oedema, but some of the ability of the barrier to prevent macro-molecular entry is retained. There is therefore a rationale for testing of small molecule inhibitors in preference of antibodies when performing targeted therapy for glioblastoma.

2.2.2 Rationale for targeting EGFR

The epidermal growth factor receptor (EGFR) (ErbB-1) is a member of a family of 4 structurally related membrane spanning receptor tyrosine kinases including ErbB-3, ErbB-4 and ErbB-2 or HER-2.

They are activated with varying efficacy by epidermal growth factor ligands. Receptor-ligand interaction leads to dimer formation and activation, which stimulates the intrinsic intracellular protein-tyrosine kinase activity. The downstream targets of EGFR include the signaling proteins with important roles in cell lineage determination and cell survival. Mutations leading to the over-expression or amplification of EGFR contribute to oncogenesis by inducing cells to proliferate and to resist apoptosis. A variety of mutations affecting the expression and function of this family of receptors have been demonstrated in a variety of cancers (Nicholson et al. 2001).

In 40-50% of cases of glioblastoma the EGFR is over-expressed and co-expression of the constitutively activated mutant variant of EGFR, the epidermal growth factor variant III (EGFRvIII) is observed in nearly half of these cases (Ekstrand et al. 1991; Frederick et al. 2000; Libermann et al. 1985; Pelloski et al. 2007; Wong et al. 1987). The Epidermal growth factor receptor variant III (EGFRvIII) is the product of a common, tumour-specific mutation of the EGFR consisting of an in-frame deletion of exons 2-7 (801bp) from its extracellular ligand–binding region.

2.2.3 Rationale for targeting both the VEGF and EGFR pathways

A network of interconnected signal transduction pathways is responsible for the development and maintenance of many solid tumours. Consequently, blockade of a single pathway may be ineffective in the long term because activation of other pathways can serve as escape mechanisms for the tumour (Scagliotti 2007).

Parallel and reciprocal pathways exist between the VEGFR and EGFR signalling cascades, clearly linking these pathways within tumours (Tabernero 2007). Therefore, dual inhibition of VEGFR and EGFR signaling cascades may be important for optimal suppression of tumour growth, and have been shown to have synergistic effects in preclinical models (van Cruijsen 2005). A randomised phase II study in non small cell lung cancer patients found a comparative effect with a more favourable toxicity profile using a combination of the VEGF antibody bevacizumab with the EGFR small molecule inhibitor erlotinib, in comparison to bevacizumab in combination with standard chemotherapy (Herbst 2007).

EGFR, as well as being involved in controlling cell proliferation and apoptosis, has also been shown to play a role in tumour angiogenesis (Ellis 2004). Activation of the EGFR pathway by either EGF or TGF increases the production of angiogenic factors (VEGF and VEGFR) in a variety of tumour cells including gastric (Akagi et al. 2003), bladder (Perrotte et al. 1999), and pancreatic cell lines (Parikh et al. 2003). Preclinical studies using high grade glioma cell lines have similarly demonstrated that EGFR expression up-regulates the production of VEGF through mechanisms distinct from those under

hypoxic regulation (Clarke et al. 2001; Maity et al. 2000; Pore et al. 2003). Conversely, inhibition of the EGFR pathway has been shown to reduce the production of angiogenic molecules in gastric, colonic, pancreatic and breast cancer cell lines (van Cruijsen et al. 2005). Of particular relevance to this study, gefitinib treatment has been shown to cause a dose- and time-dependent decrease in VEGF production in in-vitro studies of various cancer cell lines (Ciardiello et al. 2001) whilst also inhibiting the expression of other mediators of tumour-induced angiogenesis, such as cyclooxygenase-2, in squamous cell carcinoma cell lines of the head and neck (Chen et al. 2004).

Additional evidence that the EGFR and VEGFR pathways are linked has come from a recent study demonstrating that inhibition of the downstream EGFR-mediated effector, mammalian target of rapamycin (mTOR), reduces VEGF expression and capillary tube formation by endothelial cells (Bianco et al. 2008). Further support justifying the strategy of dual inhibition came from the discovery that combined inhibition of multiple targets has the potential to overcome resistance to monotherapies (Rubin and Duensing 2006). In one preclinical study, Van Cruijsen et al. demonstrated that resistance to anti-EGFR therapy could be overcome by adding antiangiogenic therapy to an anti-EGFR regimen (Van Cruijsen et al 2005).

Although some patients initially respond to EGFR TKIs, nearly all eventually acquire resistance to therapy following multiple or prolonged treatment (Camp et al. 2005). Resistance can be acquired via several mechanisms. Mutation or over-activity of EGFR-independent signaling pathways, such as the acquisition of K-ras mutation, or loss of PTEN expression are often responsible. Conformational changes in the TKI binding domain, and ligand-independent activation may also occur. The induction of higher angiogenic potential via upregulation of VEGF and other pro-angiogenic molecules by tumour cells has also been shown to be play a role (Ciardiello et al. 2004; Naumov et al. 2009; Viloria-Petit et al. 2001; Viloria-Petit and Kerbel 2004). If this is the case, then dual inhibition of both pathways may act to prevent resistance to EGFR inhibition through VEGFR.

2.3 Agents in the trial

2.3.1 Cediranib

Cediranib is a potent, orally bio-available small molecule inhibitor of the vascular endothelial growth factor receptor (VEGFR) tyrosine kinases (Wedge et al. 2005). Cediranib is active against each variant of VEGFR with particular potency against VEGFR-2, the main mediator in endothelial cell proliferation, differentiation and vascular permeability. It also has activity against both platelet derived growth factor receptor (PDGFR) and the c-Kit receptor, which are implicated in angiogenesis and cell cycle

regulation respectively. Adopting a small molecule approach to the inhibition of angiogenesis may improve upon the efficacy already observed with bevacizumab in glioblastomas, by reducing the potential influence of the blood-brain barrier.

Common side effects associated with cediranib include diarrhoea, dehydration, hand and foot syndrome, fatigue, nausea and vomiting, muscle weakness, dry or sore mouth, tiredness, hoarseness, proteinuria, hypertension, bleeding, altered thyroid function, increased transaminases, thrombocytopenia, anorexia, weight loss, headache and GI perforation. Reversible posterior leukoencephalopathy syndrome has been associated with cediranib but according to CIOMS criteria is not common (> 0.1%, <1%). See the current Investigators Brochure for more details.

2.3.2 Gefitinib (Iressa, ZD1839)

Gefitinib is an orally administered, small molecule inhibitor of the EGFR tyrosine kinase; it competes with adenosine triphosphate (ATP) for binding sites on the catalytic domain of the receptor, inhibiting auto-phosphorylation and activation.

Common side effects associated with gefitinib include diarrhoea, skin reactions, nausea, vomiting, pruritus (very common), asthenia (very common) proteinuria, haemorrhage (such as epistaxis and haematuria), interstitial lung disease (1.3%), anorexia and liver function test disturbances. Rare side effects (about 1%) include bullous conditions including Toxic epidermal necrolysis, Stevens Johnson syndrome and cutaneous vasculitis, and hepatitis. Gefitinib might also cause increased sensitivity to sunlight. See the current SmPC for more details.

The gefitinib SmPC mentions that in a phase I/II trial studying the use of gefitinib and radiation in paediatric patients, with newly diagnosed brain stem glioma or incompletely resected supratentorial malignant glioma, 4 cases (1 fatal) of Central Nervous System (CNS) haemorrhages were reported from 45 patients enrolled. Patients on the trial should therefore be monitored closely for haemorrhagic events which may not be simply due to disease progression.

2.4 Clinical trial experience

2.4.1 Cediranib

Trial title	Phase	Identifier	Recruiting
AZD2171 in Treating Patients With Recurrent Glioblastoma Multiforme	II	NCT00305656	No
Cediranib in Combination With Lomustine Chemotherapy in Recurrent Malignant Brain Tumour	I	NCT00503204	No
AZD2171 and Whole Brain Radiation Therapy (WBRT) in Patients With Brain Metastases	I/II	NCT00937482	Yes
AZD2171 in Treating Young Patients With Recurrent, Progressive, or Refractory Primary CNS Tumors	I	NCT00326664	Yes
Bevacizumab and AZD2171 in Treating Patients With Metastatic or Unresectable Solid Tumor, Lymphoma, Intracranial Glioblastoma, Gliosarcoma or Anaplastic Astrocytoma	I	NCT00458731	Yes
Cediranib Maleate and Cilengitide in Treating Patients With Progressive or Recurrent Glioblastoma	I	NCT00979862	Not yet open
Gamma-Secretase Inhibitor RO4929097 and Cediranib Maleate in Treating Patients With Advanced Solid Tumors	I	NCT01131234	Yes
Cediranib, Temozolomide, and Radiation Therapy in Treating Patients With Newly Diagnosed Glioblastoma	I/II	NCT00662506	Yes
Temozolomide and Radiation Therapy With or Without Cediranib Maleate in Treating Patients With Newly Diagnosed Glioblastoma	II	NCT01062425	Yes

Table 1 Trials of cediranib (AZD2171) in brain cancer (www.clinicaltrials.gov)

2.4.1.1 Phase III study of cediranib versus lomustine versus cediranib plus lomustine in glioblastoma

The results of a recent phase III trial in recurrent glioblastoma using cediranib and lomustine (the REGAL trial) were presented at the European Society for Medical Oncology (ESMO) congress, 2010 (Bachelor et al, 2010). Patients were randomised at 2:2:1 to cediranib alone (30mg od), cediranib (20mg od) plus lomustine (110mg/m^2 every 6 weeks), or lomustine alone (110mg/m^2 every 6 weeks) and placebo.

325 patients were randomised: Ced (n=131), Ced + Lom (n=129) or Lom (n=65). The study showed no statistical difference between either cediranib arm vs. lomustine alone on the primary endpoint of progression free survival (Ced vs Lom: HR=1.05, 95% CI 0.74–1.50, P=0.899; Ced + Lom vs. Lom:

HR=0.76, 95% CI 0.53–1.08; P=0.162 [median PFS (days): 92 (ced), 125 (ced+ lom), 82 (lom)]), or for overall survival (Ced vs Lom: HR=1.43, 95% CI 0.96–2.13, P=0.100; Ced + Lom vs. Lom: HR=1.15, 95% CI 0.77–1.72; P=0.499 [median OS (months): 8.0 (ced), 9.4 (ced + lom), 9.8 (lom)]). Cediranib did show evidence of clinical activity at other endpoints, including a statistically significant increase in the time to deterioration of neurological status when cediranib was given with lomustine compared to lomustine alone (Ced vs Lom:HR=0.82, 95% CI 0.55–1.22, P=0.573; Ced + Lom vs. Lom: HR=0.63, 95% CI 0.42–0.95, P=0.009 [median TDNS (days): 126 (ced), 170 (ced + lom), 111 (lom)]). There was also a numerical increase in the cediranib-containing arms with regards to response rate – which was not determined by RANO criteria (Wen 2010), (15.3% (ced), 17.2% (ced + lom), 8.9% (lom)). The mean change in steroid use from baseline to progression was significantly reduced for both cediranib arms when compared to lomustine alone (-26.2% (ced), -23.4% (ced + lom), +5.3% (lom); ced vs lom = p0.006, ced+lom vs lom= p0.012). There was no increased risk of fatal outcome or intracranial bleeding in the cediranib arms.

Baseline characteristics were balanced across treatments, although the lomustine arm did have a slightly higher Karnofsky Performance Status and lower steroid use compared to the other arms.

Notably early effects were seen with cediranib (as demonstrated by change in contrast-enhancing area and response rate) which were not maintained throughout the trial. Many of the patients on the lomustine arm went on to receive bevacizumab as subsequent therapy after the trial, influencing the overall survival S for these patients and thus confounding the overall survival findings. The lomustine alone arm had a relatively small number of patients in the context of a phase III trial and a few patients had a good response which influenced the overall outcome. Despite some weaknesses in design this trial showed evidence of clinical activity of cediranib on some endpoints.

2.4.1.2 Trials using cediranib in other cancers

Cediranib has shown activity in other cancers including metastatic colorectal cancer, although trials have yet to show a clinical advantage over bevacizumab in this setting (HORIZON II and III trials) (as reported by AstraZeneca, 2010).

2.4.2 Gefitinib

Trial title	Phase	Identifier	Recruiting
A Phase II Study of Gefitinib in Benefited Patients With Asymptomatic Brain Metastasis Advanced Non-Small Cell Lung Cancer by Chemotherapy	II	NCT00614809	No
ZD1839 (Iressa) And Radiation In Pediatric Patients Newly Diagnosed With Brain Stem Tumors Or Incompletely Resected Supratentorial Malignant Gliomas With Phase II Limited To Brain Stem Tumors	I/II	NCT00042991	No
Whole Brain Radiotherapy in Combination With Gefitinib (Iressa) or Temozolomide (Temodal) for Brain Metastases From Non-Small Lung Cancer (NSCLC) A Randomized Phase II Trial	II	NCT00238251	No
A phase I study of ZD1839 and temozolomide for the treatment of gliomas	I	NCT00027625	No
ZD1839 FOR Treatment Of Recurrent Or Progressive Malignant Astrocytoma Or Glioblastoma And Recurrent Or Progessive Meningioma: A Phase II Study With A Phase I Component For Patients Receiving EIAEDs (enzyme-inducing anti-epileptic drugs)	I/II	NCT00025675	No
A Phase I/II Trial to Assess the Tolerability of RAD 001 With Gefitinib in Patients With Glioblastoma Multiforme and Prostate Cancer and Efficacy in Patients With Castrate Metastatic Prostate Cancer	I/II	NCT00085566	No
A Phase II Study of ZD 1839 (NSC 715055) for Patients With First Relapse Glioblastoma Multiforme	II	NCT00016991	No
A Phase II Exploratory, Multicentre, Open-Label, Non-Comparative Study of ZD1839 (Iressa™) and Radiotherapy in the Treatment of Patients With Glioblastoma Multiforme	II	NCT00238797	No
A Phase I/II Study Of An Oral Epidermal Growth Factor Receptor Tyrosine Kinase Inhibitor (EGFR-TKI), ZD 1839 (IRESSA) [NSC# 715055] With Radiation Therapy In Glioblastoma Multiforme	I/II	NCT00052208	No
Phase II Study Of ZD 1839 (NSC 715055) In Newly Diagnosed Patients With Glioblastoma	II	NCT00014170	No
Phase I Study of High Dose Gefitinib (Iressa) for the Treatment of Carcinomatous Meningitis in Adult Patients With Non-Small Cell Lung Cancer and Known or Suspected EGFR Mutations	I	NCT00372515	No

Table 2 Trials of gefitinib in brain cancer (www.clinicaltrials.gov)

Phase II studies using single agent gefitinib in recurrent glioblastoma have demonstrated only modest activity in genetically unselected populations (Franceschi et al. 2007; Rich et al. 2004). The genetic heterogeneity observed in glioblastomas renders their growth less ‘addicted’ to a solitary oncogene

and hence there is an increasing acceptance of the need to appropriately combine targeted therapies (Pillay et al. 2009).

2.4.3 Trials targeting EGFR and VEGFR

Many clinical studies investigating combinations of VEGFR and EGFR blocking agents in a variety of tumour types, including breast, colon, renal, head and neck, non-small cell lung cancer, and adenocarcinoma of unknown primary have been undertaken (Bukowski et al. 2007; Cohen et al. 2009a; Dickler et al. 2008; Hainsworth et al. 2005; Hainsworth et al. 2007; Herbst et al. 2005; Saltz et al. 2007). Generally, these have been phase I or phase II trials performed as second or third line treatment in patients with recurrent or metastatic cancer, who have exhausted multiple lines of standard therapy. Overall response rates ranging from 0%-44% have been demonstrated, with stable disease being achieved in up to 65% of patients (van Cruijsen et al. 2005). Toxicity in these studies seldom necessitated discontinuation of therapy, and was limited most commonly to rash, diarrhoea, hypertension and fatigue. The combined EGFR/VEGF blocking agent Vandetanib showed promise in Phase I and II studies in advanced NSCLC (Tamura et al. 2006). The results of three phase III trials so far have shown mixed success in these patients, although many more phase III trials in NSCLC are still ongoing (Flannigan et al. 2010). No results are available yet for this drug in glioblastoma although trials are currently recruiting.

Trial title	Phase	Identifier	Recruiting
Angiogenic and EGFR Blockade With Curative Chemoradiation for Advanced Head and Neck Cancer	I/II	NCT00140556	No
Docetaxel in Combination With Zactima (ZD6474) in Patients With Locally Advanced Squamous Cell Carcinoma of the Head and Neck	II	NCT00459043	No
Bevacizumab/Tarceva and Tarceva/Sulindac in Squamous Cell Carcinoma of the Head and Neck	II	NCT00392665	No
Combining Erlotinib Plus Bevacizumab and Gemcitabine Plus Capecitabine to Treat Advanced Pancreatic Cancer (TARGET)	I/II	NCT00260364	No
Vandetanib in Treating Patients With Unresectable or Metastatic Kidney Cancer	II	NCT00608114	Yes
Combination of Bevacizumab, Pertuzumab, and Sandostatin for Advanced Neuroendocrine Cancers	II	NCT01121939	Yes
Bevacizumab and Lapatinib in Children With Recurrent or Refractory Ependymoma	II	NCT00883688	Yes
Vandetanib Gemcitabine Or Placebo Plus Gemcitabine Or Vandetanib Monotherapy In Advanced Biliary Tract Cancer (VANGOGH)	II	NCT00753675	Yes
A Targeted Ph I/II Trial of ZD6474 (Vandetanib;Zactima) Plus the Proteasome Inhibitor, Bortezomib	I/II	NCT00923247	Yes

(Velcade(Registered Trademark)), in Adults With Solid Tumors With a Focus on Hereditary or Sporadic, Locally Advanced or Metastatic Medullary Thyroid Ca (MTC)			
A Phase II Study of 2 Doses of ZD6474 (Vandetanib) in Combination With FOLFOX vs FOLFOX Alone for the Treatment of Colorectal Cancer	II	NCT00500292	No
An Efficacy Study Comparing ZD6474 to Placebo in Medullary Thyroid Cancer	II	NCT00410761	No
Zactima in Non Small Cell Lung Cancer (NSCLC) ELderly Patients In Combination With or Versus Gemcitabine	II	NCT00753714	No
Study of VandetanibCombined With Chemotherapy to Treat Advanced Non-small Cell Lung Cancer	II	NCT00687297	No
Radiation Therapy (XRT)and ZD6474 in Non-Small Cell Lung Cancer (NSCLC)	I/II	NCT00745732	No
Efficacy and Safety of Zactima™ in Patients With Metastatic Papillary or Follicular Thyroid Cancer	II	NCT00537095	No
Phase II Study of Best Support Care (BSC) Plus ZD6474 in Patients With Inoperable Hepatocellular Carcinoma (HCC)	II	NCT00508001	No
Efficacy and Safety of Zactima™ in Patients With Castration-refractory Metastatic Prostate Cancer	II	NCT00659438	No
Efficacy and Safety Study of Vandetanib (ZD 6474) in Combination With Bicalutamide Versus Bicalutamide Alone in Patients With Chemotherapy Naive Hormone Refractory Prostate Cancer.	II	NCT00757692	Yes
ZD6474 (ZACTIMA™) Phase III Study in EGFR Failures	III	NCT00404924	No
Randomized Study of Docetaxel +/- ZD6474 in Metastatic TCC	II	NCT00880334	No
Docetaxel in Combination With Zactima (ZD6474) in Patients With Locally Advanced Squamous Cell Carcinoma of the the Head and Neck	II	NCT00459043	No
Vandetanib, Oxaliplatin, and Docetaxel in Treating Patients With Advanced Cancer of the Esophagus or Gastroesophageal Junction	I/II	NCT00732745	No
ZACtima FASlodex Trial (ZACFAST)	II	NCT00752986	Yes
Phase II of Zactima Maintenance for Locally Advanced or Metastatic NSCLC Following Platinum-doublet Chemotherapy	II	NCT00777179	No
Docetaxel With or Without Vandetanib in Treating Patients With Persistent or Recurrent Ovarian Epithelial Cancer, Fallopian Tube Cancer, or Primary Peritoneal Cancer	II	NCT00872989	Yes
Vandetanib in Treating Patients With Unresectable or Metastatic Kidney Cancer	II	NCT00608114	Yes
Vandetanib to Treat Children and Adolescents With Medullary Thyroid Cancer	I/II	NCT00514046	Yes
Phase II Study of Vandetanib in Individuals With Kidney Cancer	II	NCT00566995	Yes
Docetaxel With or Without Vandetanib in Treating Patients With Metastatic Stomach Cancer or Gastroesophageal Junction Cancer	II	NCT00683787	No
Docetaxel With or Without Vandetanib in Treating Patients With Previously Treated Stage IV Transitional Cell Cancer	II	NCT00378794	Yes

Trial to Evaluate the Therapeutic Benefit of Fulvestrant in Combination With ZACTIMA in Postmenopausal Women With Bone Predominant, Hormone Receptor Positive Metastatic Breast Cancer (ZAMBONEY)	II	NCT00811369	Yes
BATTLE Program: ZD6474 in Previously Treated Subjects With NSCLC	II	NCT00410189	No
Paclitaxel, Carboplatin, and High-Dose Radiation Therapy With or Without Vandetanib in Treating Patients With Stage IIIA or Stage IIIB Non-Small Cell Lung Cancer	I/II	NCT00975260	No
Chemotherapy and Radiation Therapy With or Without Vandetanib in Treating Patients With High-Risk Stage III or Stage IV Head and Neck Cancer	II	NCT00720083	No
Efficacy and Tolerability of ZD6474 in Patients With Thyroid Cancer	II	NCT00098345	No
Carboplatin and Gemcitabine Hydrochloride With or Without Vandetanib as First-Line Therapy in Treating Patients With Locally Advanced or Metastatic Urinary Tract Cancer	II	NCT01191892	Yes
ZD6474, Carboplatin, and Paclitaxel in Treating Patients With Stage IIIB, Stage IV, or Recurrent Non-Small Cell Lung Cancer	II	NCT00093392	Yes
Cisplatin + Etoposide +/- Concurrent ZD6474 in Previously Untreated Extensive Stage Small Cell Lung Cancer	II	NCT00613626	Yes
Malignant Pleural Effusion With ZD6474	II	NCT00402896	No
Phase II Anastrozole and ZD6474 in Neoadjuvant Treatment of Postmenopausal Hormone Receptor-Positive Breast Cancer	II	NCT00481845	No
BAY 43-9006 Plus Cetuximab to Treat Colorectal Cancer	II	NCT00326495	Yes
Combination of Bevacizumab, Pertuzumab, and Sandostatin for Adv. Neuroendocrine Cancers	II	NCT01121939	Yes
PEAK: A Phase 2 Study of Panitumumab Plus mFOLFOX6 vs. Bevacizumab Plus mFOLFOX6 for First Line Treatment of Metastatic Colorectal Cancer Subjects With Wild-Type KRAS Tumors	II	NCT00819780	Yes
A Phase III Trials Program Exploring the Integration of Bevacizumab, Everolimus (RAD001), and Lapatinib Into Current Neoadjuvant Chemotherapy Regimes for Primary Breast Cancer (GeparQuinto)	III	NCT00567554	Yes

Table 3 Phase II and III Trials of combination anti-VEGF and EGFR therapy in all cancers excluding brain cancer (see Table 4) (www.clinicaltrials.gov)

2.4.3.1 Phase I study of cediranib and gefitinib

A phase I study of cediranib plus gefitinib in patients with advanced solid tumours showed this combination to be well tolerated with manageable side effects. Diarrhoea, hypertension, fatigue, anorexia, skin toxicity and altered thyroid function were most commonly seen adverse events. The maximum tolerated dose (MTD) of cediranib in combination with 250 mg gefitinib was found to be 30 mg (mainly due to dose-limiting toxicities of hypertension at 45 mg), although in combination with

500 mg gefitinib, 45 mg cediranib was tolerated. Encouraging anti-tumour activity was seen, with eight patients (9%) achieving a partial response. In addition, thirty-eight patients (42%) had stable disease, of which nine patients had confirmed reductions of 10–30% on consecutive visits (van Cruijsen et al. 2010).

2.4.3.2 Trials of VEGFR and EGFR combination therapy in glioblastoma

Trial title	Phase	Identifier	Recruiting
Phase II Bevacizumab + Erlotinib for Patients With Recurrent Malignant Glioma	II	NCT00671970	No
Combination of Bevacizumab, Pertuzumab, and Sandostatin for Advanced Neuroendocrine Cancers	II	NCT01121939	Yes
Bevacizumab and Lapatinib in Children With Recurrent or Refractory Ependymoma	II	NCT00883688	Yes
Zactima With Temodar During Radiation Treatment for Newly Diagnosed Stage IV Brain Tumors	I/II	NCT00441142	Yes
Ph I Dose Escalation Trial of Vandetanib in Combo w Etoposide for Malignant Gliomas	I	NCT00613223	Yes
Investigate the Maximum Tolerated Dose of Vandetanib and Concurrent Whole Brain Radiotherapy (WBRT) in Patients With Non-small Cell Lung Cancer (NSCLC) and Brain Metastases	I	NCT00807170	Yes
Ph I Zactima + Imatinib Mesylate & Hydroxyurea for Pts w Recurrent MG	II	NCT00613054	Yes
A Randomized Phase II Trial of Vandetanib (ZD6474) in Combination With Carboplatin Versus Carboplatin Alone Followed by Vandetanib Alone in Adults With Recurrent High-Grade Gliomas	II	NCT00995007	Yes
Vandetanib and Radiation Therapy in Treating Young Patients With Newly Diagnosed Diffuse Brainstem Glioma	I	NCT00472017	No
ZD6474 Alone and in Combination With Retinoic Acid in Pediatric Neuroblastoma	I	NCT00533169	Yes
Phase I Dose Escalation Study of Vandetanib w/ Hypofractionated Stereotactic Radiotherapy in Patients w/ Recurrent Malignant Gliomas	I	NCT00822887	Yes
Vandetanib and Sirolimus in Patients With Recurrent Glioblastoma	I	NCT00821080	Yes
Clinical Trial Evaluating the Combination of Vandetanib and Dasatinib During and After Radiation Therapy (RT) in Children With Newly Diagnosed Diffuse Intrinsic Pontine Glioma (DIPG)	I	NCT00996723	Yes
ZD6474 in Treating Patients With Recurrent or Progressive Gliomas	I/II	NCT00293566	No

Table 4 Trials of combination anti-VEGF and EGFR therapy in brain cancer (www.clinicaltrials.gov)

Early studies using single agent targeted therapies directed against EGFR in patients with recurrent glioblastoma have shown disappointing results (Franceschi et al. 2007; Rich et al. 2004). As mentioned above, such resistance to anti-EGFR therapy is thought to result, in part, from

upregulation of VEGF and other pro-angiogenic molecules by tumour cells, imparting upon them a higher angiogenic potential (Ciardiello et al. 2004; Naumov et al. 2009; Viloria-Petit et al. 2001; Viloria-Petit and Kerbel 2004). This has clear implications for the single agent use of EGFR targeted therapy in the ‘avidly vascular’ model of glioblastoma, and is the basis of the move to develop clinical trials examining the efficacy and safety of anti-VEGF and anti-EGFR targeted therapy in combination in recurrent glioblastoma. The combined EGFR/VEGF blocking agent AEE788 was well tolerated in a Phase I study in patients with recurrent glioblastoma. Seven (27%) patients achieved stable disease and progression free survival at 6 months was 14% (Reardon et al. 2005). Another combined EGFR/VEGF blocking agent vandetanib has been studied in a phase I study in combination with etoposide in recurrent glioblastoma patients. At dose level 1, higher than expected rates of grade 4 neutropaenia were observed. Consequently the dose level has been reduced, and dose escalation continues. A phase II trial is planned when the maximum tolerated dose of vandetanib with reduced dose etoposide is determined (Herndon et al 2009). In a recent phase II trial Hasselbalch et al treated forty-three patients with recurrent glioblastoma with a combination of cetuximab, bevacizumab and irinotecan. Radiological responses were noted in 34% of patients, of which two had complete responses and nine had partial responses. The 6-month progression-free survival probability was 30% and median overall survival was 29 weeks (95% CI: 23–37 weeks). However this was non-superior compared with results with bevacizumab and irinotecan alone (Hasselbalch et al. 2010). In a phase II trial of bevacizumab plus erlotinib in twenty-five patients with recurrent glioblastoma, twelve patients (48%) achieved radiological response and 6 month progression-free survival rate was 24% (Sathornsumetee et al 2008). The combination was well tolerated, with common side effects being rash, diarrhoea, mucositis and fatigue.

Given that the recent phase I study combining cediranib with gefitinib demonstrated combination treatment to be generally well tolerated, and to show encouraging antitumour activity in patients with advanced solid tumours (van Cruijsen et al. 2010), we aim to follow on directly from this and both build on the experience with cediranib as a single agent in recurrent/progressive glioblastoma and investigate whether the efficacy of cediranib is improved with the addition of gefitinib.

2.5 Proposed Trial

This is a phase II, randomised, double blind placebo controlled study in patients with recurrent or progressive glioblastoma (WHO grade IV). Patients are to receive cediranib in combination with gefitinib or cediranib with placebo.

2.5.1 Primary Objective

- The primary objective of the study is to compare the efficacy of cediranib in combination with oral gefitinib with cediranib alone.

2.5.2 Secondary Objectives

- To document the safety and tolerability of cediranib in combination with oral gefitinib and cediranib alone.
- To compare cediranib in combination with oral gefitinib and cediranib alone in terms of steroid sparing effects
- To document the quality of life and neurological symptoms reported by patients taking cediranib in combination with oral gefitinib and cediranib alone.
- To investigate the relationship between the effects of cediranib (+/- gefitinib) on levels of soluble angiogenesis biomarkers and clinical efficacy
- To investigate the relationship between the patient's genetic profile and efficacy of cediranib (+/- gefitinib)

2.6 Outcome variables

2.6.1 Efficacy

2.6.1.1 Primary outcome variable

Progression free survival (PFS) defined as the time from the date of randomisation to the date of first progression or death due to any cause, whichever one comes first.

The progression definition will be based on modified RANO criteria (Wen 2010), such that progression will be defined as the earliest time that at least one of the following occurs:

As assessed by site staff at clinical assessments:

1. **Clinical deterioration** not attributable to other causes apart from the tumour (e.g seizures, medication adverse events, complications of therapy, cerebrovascular events, infection, and so on) or changes in dexamethasone dose.
   Clinical deterioration will be defined as:
   - Decline in Karnofsky performance status from 100 or 90 to 70 or less, or a decline of at least 20 from 80 or less, or a decline from any baseline to 50 or less, for at least 7 days

- Decline in ECOG performance status from 0 or 1 to 2 or 2 to 3

2. **Failure to return for evaluation as a result of death or deteriorating condition**
   In this instance, the earliest known date of death or deterioration will be used as the date of progression. Where unknown, the clinical assessment date at which the patient did not attend will be used (only for patients known to have not returned for evaluation due to death or deteriorating condition).

As determined by retrospective radiographic central review:

3. **Any new lesion** (short axis must be ≥10 mm on at least 2 axial slices that are 5mm apart with 0mm skip and the new lesion should be outside the original tumour volume)

4. **Increase in ≥25% of sum of the products of perpendicular diameters of enhancing lesions compared with baseline scan, on stable or increasing doses of steroids (dexamethasone) compared to baseline (T1 post-contrast scan)**

5. **Clear progression of non-measureable disease** (clinically significant increase in size with a short axis ≥10mm on at least 2 axial slices that are 5mm apart with 0mm skip)

6. **Significant increase in T2/FLAIR non-enhancing lesion – on stable or increasing steroids (dexamethasone) compared with baseline or best response not caused by co-morbid events.** Precise quantification of the increase in T2/FLAIR signal can be difficult and must be differentiated from other causes of increased T2 or FLAIR signal, such radiation effects, decreased corticosteroid dosing, demyelination, ischemic injury, infection, seizures, postoperative changes, or other treatment effects before making a determination of progressive disease. Changes in T2/FLAIR signal that suggest infiltrating tumour include mass effect (as determined by sulcal effacement, ventricular compression, and thickening of the corpus callosum), infiltration of the cortical ribbon, and location outside of the radiation field. The initiation of these changes can be subtle, and convincing non–contrast-enhancing growth may require one or two confirmatory scans. If non-enhancing progression is determined after retrospective review of images, the scan at which these changes were first detected should serve as the progression scan.
   Examples of progression on T2/FLAIR:
   a. Substantial change in size of tumour with clear margins on T2/FLAIR
   b. New lesion(s) (≥10mm) which is completely distinct from original enhancing volume on T2 (≥50mm distance)

Change in signal intensity in T2/FLAIR within the original enhancing volume would not be sufficient for evidence of tumour progression.

The date of progression will be determined as being the date of the first MRI scan fitting the criteria for progression, or the date the clinical deterioration or death was first reported, whichever is earliest.

Locally reported progression should be determined by the above criteria. However, due to the possibility of pseudoprogression in these patients, a confirmatory second scan taken 6 weeks after the first should be used when making treatment decisions based on radiological progression. Please note it is not necessary to stop the trial drug in patients who have progressed only radiologically (even if confirmed at subsequent visits) if the clinician feels that the patient is benefiting from treatment.

2.6.1.2 Secondary outcome variables

- Overall survival (OS) – date from randomisation to death due to any cause
- Radiographic response rate (RR) at each assessment point
- PFS rate at 6 months (24 weeks). Progression is defined in the same way as described for the primary endpoint.
- Safety and tolerability
- Overall Survival rate at 12 months
- Time to sustained increase (≥ 21 days) in steroid dosage by ≥ 8mg/day compared to baseline or sustained nadir (≥ 21 days)

  (This is defined as time from randomisation to first increase in dexamethasone dose. An increase in dexamethasone dose is defined as a change from the baseline of ≥8mg/day for at least 21 days (i.e. the increase is sustained for at least 21 days consecutively). The earliest date when the dexamethasone dose was ≥ 8mg/day compared to baseline or sustained nadir in this period will be used for comparison between the treatment groups. Sustained nadir refers to the highest steroid dose taken during a ≥ 21 days period where the patient is consistently taking a lower dose of steroids compared to the baseline dose.)
- Average daily steroid dosage change from baseline until first progression
- Average number of progression/steroid free days

- Time to deterioration of neurological status (assessed via Neurological examination) or death

2.6.2 Patient reported outcomes (PROs)

- Neurological symptoms (visual disorder, motor dysfunction, communication deficit, and drowsiness) as measured by the EORTC brain tumour module BN-20.

- Quality of life as measured by the EORTC QLQ-C30 and other domains as measured by BN-20 questionnaire

2.6.3 Safety

- Adverse Events (AE)
- Vital signs including blood pressure (BP)
- Electrocardiogram (ECG) parameters
- Laboratory findings (clinical chemistry, haematology, urinalysis, TSH, T3 and T4)
- Physical examination including neurological examination

2.6.4 Pharmacodynamic and Pharmacogenetic

- Levels of soluble markers of angiogenesis and tumour growth including changes from baseline using optional blood samples

- Biomarker analysis from archival and postmortem tumour samples (both optional), including:

    IDH1 and 2 (Yan et al. 2009)
    MGMT methylation status
    EGFR, EGFRvIII and PTEN (Mellinghoff et al. 2005)

- Pharmacogenetic analysis using optional blood samples and/or tumour samples

Patients will give separate optional consent for donation and use of archival tumour samples and/or trial blood samples, and/or post-mortem tumour samples.

2.7 Trial activation

UCL CTC will ensure that all trial documentation has been reviewed and approved by all relevant bodies and that the following have been obtained prior to activating the trial:

- Research Ethics Committee approval
- Clinical Trial Authorisation from the Medicines and Healthcare products Regulatory Agency (MHRA).
- ‘Adoption’ into NIHR portfolio
- NHS permission
- Adequate funding for central coordination
- Confirmation of sponsorship
- Adequate insurance provision

3.0 Selection of Sites/Site Investigators

3.1 Site selection

In this protocol trial **"Site"** refers to the hospital or site where trial-related activities are actually conducted.

Sites must be able to comply with:

- Trial treatment(s), imaging, follow up schedules and all requirements of the trial protocol
- Requirements of the Research Governance Framework and the Medicines for Human Use (clinical trials) Act (SI 2004/1031 and all amendments)
- Data collection requirements
- Collection, preparation, temporary storage and shipment of biological samples where agreed

3.1.1 Selection of Principal Investigator and other investigators at sites

Sites must have an appropriate Principal Investigator (PI) i.e. a health care professional authorised by the site and ethics committee to lead and coordinate the work of the trial on behalf of the site. Other investigators at site wishing to participate in the trial must be trained and approved by the PI. All investigators must have experience of treating glioblastoma, have consultant status and routinely present cases to a Multi-Disciplinary Team.

Once the site has been activated by UCL CTC, the PI is responsible for ensuring:

- Adherence to the most recent version of the protocol
- All relevant site staff are trained in the protocol requirements
- Appropriate recruitment and medical care of patients in the trial
- Timely completion and return of CRFs
- Prompt notification and assessment of **all** adverse events
- Storage and shipment of biological samples where applicable

3.2 Site initiation and activation

3.2.1 Site initiation

Site initiation will be performed for each site by a site visit.

The following documentation must be in place prior to a site being opened to recruitment by UCL CTC trial team:

- Trial specific Declaration of Participation/Site Registration Form including signature and delegation log (completed by all members of the core trial team at the site (research nurses, trial administrators, lead pharmacy staff responsible for the trial), and signed off by the Principal investigator)
- All relevant institutional approvals (e.g. local NHS permission)
- A signed Clinical Trial Site Agreement (CTSA) between the Sponsor and the relevant institution (usually a NHS Trust)

The PI, other delegated site investigators and all staff involved in the conduct of the trial at the site must be identified on the site delegation log held at site and copied to UCL CTC prior to site activation.

Sites must also have in place facilities for providing **24 hour medical advice** for trial patients.

3.2.2 Site activation

Once the trial team at UCL CTC have confirmed that all documentation is in place a site activation letter will be issued to the Principal Investigator, at which point the site may start to approach patients.

4.0 Informed consent

Sites are responsible for assessing a patient's capability to give informed consent.

Sites are responsible for ensuring all patients have been given the current version of the patient information sheet, are fully informed about the trial and have confirmed their willingness to take part in the trial by signing a consent form. In addition to the consent form to indicate consent to the trial, there are additional consent forms to indicate that the patient is willing to donate any of the optional biological specimens for the trial (archival biopsy samples, blood samples throughout the trial, and/or a post mortem tumour sample). The PI or other delegated site staff are required to provide a full explanation of the trial and all relevant treatment options to each patient prior to trial entry. During these discussions the current detailed patient information sheet for the trial will be given to the patient. A minimum of twenty four hours must be allowed for the patient to consider and discuss participation in the trial. Written informed consent on the current version of the consent forms for the trial must be obtained before any trial-specific procedures are conducted.

For patients in this trial, consent will also be obtained from the patient's named carer, because the carer may be required to assist with CRF completion should the patient's condition deteriorate or in the event that the patient is unable to speak or write. In these circumstances the carer may give answers on behalf of the patient for questions regarding quality of life, Karnofsky performance score, health economics, and adverse events.

Site staff are responsible for:

- checking that information on the consent forms are complete and legible
- checking that the patient has completed/initialled all relevant sections and signed and dated the form
- Checking that an appropriate member of staff has countersigned and dated the consent forms to confirm that they provided information to the patient
- Checking that an appropriate member of staff has made dated entries in the patient's medical notes relating to the informed consent process (i.e. info given, consent signed etc.)
- Adding the patient trial number to all copies of the consent form

The original signed consent form and a copy must be stored at site (in the Investigator Site File and the patient's medical notes). A further copy must be given to the patient.

The right of the patient to refuse to participate in the trial without giving reasons must be respected. All patients are free to withdraw at any time (see section 13.0 – withdrawal of patients).

5.0 Selection of Patients

5.1 Patient Eligibility

There will be no exception to the eligibility requirements at the time of randomisation. Queries in relation to the eligibility criteria should be addressed prior to calling/faxing for randomisation. Patients are eligible for the trial if all the inclusion criteria are met and none of the exclusion criteria applies.

5.1.1 Inclusion Criteria

For inclusion in the clinical trial, patients must fulfil all of the following criteria:

- Provision of informed consent
- Age ≥18 years
- Life expectancy ≥ 12 weeks
- Histological/cytological confirmation of glioblastoma (WHO grade IV)
- Patients with measurable disease (contrast-enhancing tumour ≥10 mm by shortest diameter on 2 axial slices) by MRI imaging within 7 days prior to enrolment. (If patients have recently had a routine MRI scan, this should be assessed before deciding whether or not to screen the patient, and booking the screening/baseline MRI.)
- Patients must have been on no steroids or a stable dose of steroids (dexamethasone) for at least 5 days before the baseline MRI
- Patients must have completed standard first-line treatment for glioblastoma including surgery (with exception, if patient does not receive surgery as part of first-line treatment due to anatomical location, based on neurosurgeon's assessment), cranial radiotherapy and chemotherapy with concomitant temozolomide.
    - It is not essential that the entire Stupp regimen of 6 cycles of adjuvant temozolomide following chemoradiotherapy has been completed.
    - The last dose of temozolomide must be more than 28 days from enrolment**.**
    - Gliadel® wafers are permitted, as it is part of local treatment.
    - No other previous treatment for glioblastoma is permitted (other than steroids).

- Patients must have a Karnofsky Performance Score of 70 or above

- Patients must have a mini-mental status examination score of 15 or greater

- Patients who require either oral anticoagulants (coumadin, warfarin) or low molecular weight heparin are eligible provided there is increased vigilance with respect to monitoring INR.

For inclusion in the genetic research, patients must fulfil the following criterion:

- Provision of informed consent for genetic research (separate consent required for tumour biopsy, blood sample, and post mortem donations)

If a patient declines to participate in any of the genetic research, there will be no penalty or loss of benefit to the patient. The patient will not be excluded from other aspects of the study described in this Clinical Study Protocol, so long as they consent to the main study.

5.1.2 Exclusion Criteria

Any of the following is regarded as a criterion for exclusion from the study:

- Patients on enzyme-inducing anti-epileptic drugs (see section 7.5) within 2 weeks prior to study enrolment. Note: Patients are eligible if they switched to non-enzyme inducing agents and discontinued enzyme-inducing agents for more than or equal to 2 weeks prior to randomisation

- Inadequate bone marrow reserve as demonstrated by an absolute neutrophil count $\leq 1.5 \times 10^9$ /L or platelet count $\leq 100 \times 10^9$ /L or requiring regular blood transfusions to maintain haemoglobin >9g/dL

- Serum bilirubin ≥1.5 x ULRR (except for patients with known documented cases of Gilbert's Syndrome)

- ALT or AST ≥5 x ULRR

- Serum creatinine >1.5 x ULRR or a creatinine clearance of ≤50mL/min calculated by Cockcroft-Gault

- Greater than +1 proteinuria on two consecutive dipsticks taken no less than 1 week apart unless urinary protein <1.5g in a 24 hr period or UPC (Urine Protein: Creatinine) ratio <1.5

- History of significant gastrointestinal impairment, as judged by the investigator, that would significantly affect the absorption of cediranib or gefitinib, including the ability to swallow the tablet whole

- Patients with a history of poorly controlled hypertension with resting blood pressure >150/100mmHg in the presence or absence of a stable regimen of anti-hypertensive therapy, or patients who are requiring maximal doses of calcium channel blockers to stabilise blood pressure

- Any evidence of severe or uncontrolled diseases (e.g. unstable or uncompensated respiratory, cardiac, hepatic or renal disease)

- Unresolved toxicity >CTC AE grade 1 from previous anti-cancer therapy (including radiotherapy) except alopecia (if applicable)

- Mean QTc with Bazetts correction >470msec in screening ECG or history of familial, long QT syndrome (see Appendix 7)

- Cardiac ventricular arrhythmias requiring anti-arrhythmic therapy

- Significant haemorrhage (>30mL bleeding/episode in previous 3 months) or haemoptysis (>5mL fresh blood in previous 4 weeks)

- Recent (<14 days) major surgery or brain biopsy. Recent craniotomy (<28 days) prior to first dose, or a surgical incision that is not fully healed

- Pregnant or breast-feeding women or women of childbearing potential with a positive pregnancy test prior to receiving study medication (see section 5.1.3)

- Known hypersensitivity to cediranib, gefitinib or any of its excipients

- History of other malignancies (except for adequately treated basal or squamous cell carcinoma or carcinoma *in situ*) within 5 years, unless the patient has been disease free for 2 years and they have tissue diagnosis of the target lesion

- Known infection with hepatitis B or C or HIV

- Involvement in the planning and conduct of the study (applies to both UCL CTC, AstraZeneca staff and staff at the study site)
- Past medical history of interstitial lung disease, idiopathic pulmonary fibrosis, drug-induced interstitial disease, radiation pneumonitis which required steroid treatment or any evidence of clinically active interstitial lung disease
- Previous enrolment as part of the present study
- Treatment with an investigational drug within 30 days prior to the first dose of cediranib/gefitinib
- Other concomitant anti-cancer therapy except steroids (dexamethasone only)
- Previous anti-angiogenesis (e.g. bevacizumab, sorafenib, sunitinib) therapy
- Previous anti-EGFR treatments (e.g. cetuximab, panitumumab or small molecule tyrosine kinase inhibitors etc.) or downstream targets e.g. mTOR inhibitors.
- Patients with evidence of any intratumoural or peritumoural haemorrhage deemed significant by the treating physician
- Patients who have received any form of cranial radiation within 3 months prior to study entry (excluding imaging)
- Patients who have progressed within 3 months of completion of standard cranial radiation
- Patients that have received radiosurgery or brachytherapy
- Patients on >8mg/day dexamethasone or equivalent steroids on any day of the 2 weeks prior to randomisation

Restrictions

- For elective surgery during the study, or any procedure that carries a risk of internal bleeding, it is recommended that cediranib be stopped for 2 consecutive weeks prior to the surgical procedure. Cediranib treatment can be restarted when the surgical wound has healed. If emergency surgery is performed, precautions should be taken to minimise the potential risk of bleeding and thrombosis associated with this class of agents, cediranib should be stopped and

close monitoring for bleeding, wound healing and thromboembolic complications should be initiated. Patients should not receive cediranib within 2 weeks of major surgery.

- Caution in the concomitant use of any medication that may markedly affect renal function. Such medications may be used with caution if deemed essential for treatment or may be continued if already in use prior to entry in the study with no effect on renal function.

- Caution should be exercised in concomitant use of any medication that may significantly affect hepatic P450 drug metabolising activity by way of enzyme induction or inhibition within 2 weeks of the first dose of cediranib or gefitinib and throughout the study period (see Appendix 3 for a list of drugs)

- Patients who are blood donors should not donate blood during the study and for 3 months following their last dose of study treatment

- Medicinal products that cause significant sustained elevation in gastric pH, such as proton pump inhibitors and h2 antagonists may reduce bioavailability and plasma concentrations of gefitinib and therefore, may reduce efficacy. Antacids if taken regularly close in time to administration of gefitinib may have a similar effect. These should be avoided where possible.

- Patients should take their medication at least 1 hour prior to the consumption of a meal or at least 2 hours after a meal has been ingested.

5.1.3 Pregnancy and Birth Control

The potential risk to patients who conceive or are pregnant whilst taking study medication is not known, although reproductive toxicity has been reported in animals administered with either gefitinib or cediranib. Women of childbearing potential will be asked to take a pregnancy test by a healthcare professional at the screening assessment within 14 days of starting trial treatment.

Women of child bearing potential will be defined as all female trial participants who are not postmenopausal. Postmenopausal females are defined as:

- Natural menopausal with menses >2 years ago
- Radiation-induced oophorectomy with last menses > 2 years ago
- Chemotherapy-induced menopause with 2 years interval since last menses

- Serum follicle stimulating hormone, luteinising hormone and plasma oestradiol levels in the postmenopausal range for the institution
- Bilateral oophorectomy or hysterectomy

Contraception

Female patients on the study must be post-menopausal, surgically sterile, sexually abstinent or use two reliable forms of contraception from starting cediranib and/or gefitinib and for 2 weeks after the last dose. Reliable methods of contraception should be used consistently and correctly, acceptable methods include barrier methods, implants, injectables, combined oral contraceptive methods, some IUDs, or partner had a vasectomy.

Male patients must use a barrier method of contraception from starting trial medications and for 2 weeks after the last dose.

In the event of a pregnancy occurring on the trial, please report immediately to the UCL CTC (see section 10.6 Pregnancy reporting).

5.2 Pre-randomisation evaluation

Patients must give written informed consent before any trial specific screening investigations may be carried out. The following assessments or procedures are required to evaluate the suitability of patients prior to entry:

- Confirmation (histological/cytological) that patient has glioblastoma (WHO grade IV)
- Within 7 days prior to randomisation:
    - Brain Tumour assessment: MRI scan of the tumour region, where the tumour is the largest. –See section 7.7 (Imaging) for the list of required scans.
    - Demographic details including date of birth, sex, and race
    - Relevant medical and surgical history, including previous hypertension, ongoing medication and documentation of baseline symptoms
    - Physical examination: height and weight, vital signs (including pulse, lying and 5 min standing blood pressure, respiratory rate and temperature)
    - ECOG performance status
    - Karnofsky performance status

- Mini-mental status exam
- Neurological examination
- Full blood count (FBC)
- Serum biochemistry: sodium, potassium, urea, creatinine, phosphate, total protein, albumin, globulin, adjusted calcium, total bilirubin, alkaline phosphatase, AST and/or ALT, LDH and gamma-GT.
- Clotting function tests (PT/APTT)
- Thyroid function tests
- Urinalysis: dip-stick test for protein. If a patient has two consecutive urine protein measurements of greater than one plus (+) taken no less than 1 week apart then measure urine protein:creatinine ratio or collect a 24-hour urine for total protein. A urine protein/creatinine ratio of 0.15 (urine protein and urine creatinine expressed in mg/dl) approximates a 24-hour urine protein of 150mg/24 hours or 0.15 g/24 hours, which is the upper limit of normal.
- Pregnancy test (if applicable – i.e females of child bearing potential (see section 5.1.3))
- ECG with estimation of QTc interval (see Appendix 7)

5.3 Screening Log

A screening log should be maintained by the site and kept in the Investigator Site File. This should record each patient screened for the trial and whether or not they were randomised to the trial. If they were not randomised the reasons should be given. The log should be sent to UCL CTC when requested with patient identifiers removed prior to sending.

6.0 Randomisation procedures

6.1 Randomisation

Patient randomisation will be performed centrally at the UCL CTC, after the relevant site has faxed the fully completed randomisation form to UCL CTC and it has been checked by a trained UCL CTC staff member to confirm eligibility and informed consent. Randomisation must be performed prior to commencement of any trial treatment. Patient randomisation will be performed using an interactive web-based recognition system (Cenduit IWRS), with a link to an Access randomisation programme. Randomisation will be undertaken using minimisation, and stratified by age (<65 or ≥65yrs) and resection for the recurrent disease (previous resection / no previous resection), Karnofsky performance status (>80 / ≤80). Within each stratum patients will be randomised in a 1:1 ratio to the treatment arms.

Sites should ensure that the following have been completed before randomising:

- Patient consent form must be signed
- Pre-randomisation evaluations should be carried out at sites as detailed in Section 5.3 (following consent if not done routinely)
- The randomisation form must be fully completed prior to faxing the UCL CTC
- Screening MRI scan must have been completed (a copy should be sent to UCL CTC following randomisation, see Section 7.7)

On receipt of completed randomisation form at UCL CTC:

- If the patient is confirmed as eligible, a patient trial number will be assigned at UCL CTC
- The UCL CTC will then randomise the patient on the Cenduit interactive web-based response system (IWRS). The IWRS will send an e-mail to site staff and pharmacy, notifying them of medication codes which correspond to stock sufficient to cover the patient's first 6 weeks of treatment i.e. 6 weeks of daily cediranib plus daily gefitinib/placebo. Site staff will be responsible for using IWRS for drug resupply visits (for more information, refer to the IWRS user manual).
- A unique patient trial number will be assigned. The UCL CTC will fax a confirmation form to the site's lead research contact and also the lead pharmacist, confirming both the patient's inclusion in the trial and their trial number. The site should then enter the patient's trial number in the randomisation CRF and arrange for the MRI scan to be sent to the UCL CTC.

- The original randomisation form should be posted to UCL CTC and a copy kept at site.
- Case report forms will be sent by UCL CTC to the main contact at site.

**Randomisation telephone number:**	+44 (0)20 7679 9860
**Randomisation fax number:**	+44 (0)20 7679 9861
**Office hours:**	09:00 to 17:00 Monday to Friday (UK Time)

Once a patient has been randomised onto the trial they must be provided with the following:

- A copy of their signed consent form(s) and patient information sheet
- A patient contact card. Site on-call contact details for out-of-hours medical care must be added to this card and patients informed to carry this with them at all times while on the trial

The baseline assessment should be carried out on the day that study treatment begins, which must be within 2 weeks of the screening assessment. If screening laboratory and ECG tests were carried out more than 7 days prior to baseline these should be repeated at baseline. The baseline MRI should be within 7 days before randomisation. Baseline assessments should be carried out as per Section 8.1 and Section 5.2.

Interactive Web-based Recognition System (IWRS)

The IWRS will be used for randomised allocation, trial medication assignment, initial drug supply and resupply, discontinuation from study treatment, emergency code breaks (i.e. unblinding) and trial drug shipment confirmation. The IWRS technology will be managed and maintained by Cenduit, in collaboration with Fisher Clinical Services. The system is accessible via the internet 24 hours a day, 7 days a week. The IWRS user manual will be provided to all sites, and contains full details regarding the operation and use of the system.

6.2 Trial drug supply

6.2.1 Initial trial drug supply

An initial supply of cediranib, gefitinib and placebo will be shipped to each site by Fisher upon site activation by the UCL CTC, via the Cenduit/Fisher IWRS. This initial supply is prompted by the UCL

CTC using the IWRS to confirm each site activation. Please see the Pharmacy Site File, IWRS User Guide, and Pharmacy Manual for further details.

6.2.2 Resupply of cediranib & gefitinib/placebo

Resupply of cediranib and gefitinib/placebo will be managed automatically by IWRS, which will count down as patients are allocated trial stock. A resupply shipment will be triggered once a site's stock becomes sufficiently low. However, site pharmacies will be able to manually request additional stock via the IWRS. For exceptional circumstances (e.g. a site intending to randomise several patients on one day), **sites will need to provide the UCL CTC with a week's notice where possible**, so that IWRS is able to ensure that sufficient stock is provided to meet the site's dispensing requirements.

7.0 Trial Treatment

7.1 Treatment summary

For the purpose of this protocol, the active IMPs are cediranib and gefitinib, with gefitinib-matched placebo.

Enrolled subjects will be randomised to receive **either** cediranib 30mg od orally and gefitinib 500mg od orally **or** cediranib 30mg od plus placebo. Each cycle of treatment lasts 6 weeks.

Treatment will continue until confirmation of progression as defined in 2.6.1.1, patient decision or the development of unacceptable toxicity (if there is radiological progression only treatment can continue if the investigator has the opinion that the patient is receiving benefit. It is not compulsory to stop trial treatment at any indication of radiological progression, even at confirmed radiological progression on multiple scans if it is felt that the patient is receiving benefit. Investigators should be wary of pseudoprogression on MRI scans.).

If none of the above are thought to have occurred on the last day of the treatment cycle, the patient will start the next cycle of treatment the following day. Treatment pauses of up to 14 days are permitted (21 days are permitted in consultation with the CI) but should be recorded on the next CRF. Treatment delays of longer than 14 days (or 21 days in consultation with the CI) will result in patient withdrawal from the study.

7.2 Summary Treatment Schedule

Patients randomised to receive cediranib and gefitinib will receive their drugs according to the following schedule:

**Week**	**1**							**2**							**3**						
Cediranib 30mg od orally	•	•	•	•	•	•	•	•	•	•	•	•	•	•	•	•	•	•	•	•	•
Gefitinib 500mg od orally	•	•	•	•	•	•	•	•	•	•	•	•	•	•	•	•	•	•	•	•	•
**Week**	**4**							**5**							**6**						
Cediranib 30mg od orally	•	•	•	•	•	•	•	•	•	•	•	•	•	•	•	•	•	•	•	•	•
Gefitinib 500mg od orally	•	•	•	•	•	•	•	•	•	•	•	•	•	•	•	•	•	•	•	•	•

Patients randomised to receive cediranib and placebo will receive their drugs according to the following schedule:

Week	1							2							3						
Cediranib 30mg od orally	•	•	•	•	•	•	•	•	•	•	•	•	•	•	•	•	•	•	•	•	•
Placebo 500mg od orally	•	•	•	•	•	•	•	•	•	•	•	•	•	•	•	•	•	•	•	•	•
**Week**	**4**							**5**							**6**						
Cediranib 30mg od orally	•	•	•	•	•	•	•	•	•	•	•	•	•	•	•	•	•	•	•	•	•
Placebo 500mg od orally	•	•	•	•	•	•	•	•	•	•	•	•	•	•	•	•	•	•	•	•	•

For both arms, each cycle will last 6 weeks and treatment will continue until progression as defined in 2.6.1.1, patient decision or the development of unacceptable toxicity (if there is progression treatment can continue if the investigator has the opinion that the patient is receiving benefit). There is therefore not a set number of treatment cycles.

At the beginning of each treatment cycle, all patients will be dispensed enough drug for one cycle (6 weeks) plus an extra weeks worth of tablets in case of delayed cycle start assessments. This is to prevent unnecessary treatment delays. Unused tablets must be returned to the pharmacy for accountability and destruction.

7.3 Trial Treatment Details

**Name of IMP**	Cediranib
**Status of drug**	Unlicensed
**Supplied by**	AstraZeneca
**Pharmaceutical form**	35 x film-coated tablets per bottle, containing 30mg cediranib/tablet (20mg and 15mg tablets also available where dose reductions are necessary, see section 7.3.4). Other ingredients are mannitol, dibasic calcium phosphate anhydrous, sodium starch glycolate and magnesium stearate with a film coat containing hypromellose 2910, polyethylene glycol 400, red iron oxide, yellow iron oxide, black iron oxide and titanium dioxide
**Dose and scheduling**	30mg once daily* (1 tablet)
**Mode of administration**	Orally (in the morning at least one hour before food. If a meal is taken before the drug at least two hours should elapse before taking cediranib)
**Storage**	Less than 30°C. Protect from light, protect from moisture.

Name of IMP	Gefitinib (Iressa)
Status of drug	Licensed for treating Non Small Cell Lung Cancer. Not licensed for treatment indication (Glioblastoma).
Supplied by	AstraZeneca
Pharmaceutical form	100 x film-coated tablets per bottle, containing 250mg of gefitinib/tablet. Other ingredients are lactose monohydrate, microcrystalline cellulose (E460), croscarmellose sodium, povidone (K29-32) (E1201), sodium laurilsulfate, magnesium stearate, hypromellose (E464), macrogol 300, titanium dioxide (E171), yellow iron oxide (E172) and red iron oxide (E172).
Dose and scheduling	500mg once daily* (2 tablets)
Mode of administration	Orally (may be taken with or without food)
Storage	Less than 30°C

Name of IMP	Placebo to match Gefitinib
Status of drug	Unlicensed
Supplied by	AstraZeneca
Pharmaceutical form	100 x 250mg film-coated tablets identical to Gefitinib per bottle. The tablets contain lactose monohydrate, microcrystalline cellulose, croscarmellose sodium, povidone, sodium lauryl sulphate and magnesium stearate with a film coat containing hypromellose, macrogol 300, red iron oxide, yellow iron oxide and titanium dioxide.
Dose and scheduling	500mg once daily* (2 tablets)
Mode of administration	Orally (may be taken with or without food)
Storage	Less than 30°C

*unless dose reduction required, see section 7.3.4.

Cediranib and gefitinib/matching placebo should be taken at approximately the same time each day, except on days they have a study visit, at which time patients should not take their study medication until after their assessments.

Cediranib, gefitinib and placebo will be packaged in high-density polyethylene (HDPE) bottles fitted with child resistant caps and tamper evident induction seals.

7.3.1 Treatment compliance

Patients should be given clear instructions on how and when to take their study drugs. The investigator or pharmacy must retain records of all study drugs administered. The UCL CTC will check these records to confirm compliance with the protocol administration schedule.

Patients should be encouraged to record when they take their study drugs each day in patient diaries.

Any dose reductions will be documented on the CRFs, along with reasons for the dose reduction. If a patient forgets to take a tablet, and it is within 6 hours of the scheduled time, then the patient should be advised to take the tablet as soon as possible. If it is more than 6 hours after the scheduled time, then study medication should not be taken for that day. Study medication should continue as previously scheduled on the subsequent day. A patient should not take more than a single day's dose of tablets within a day. Use of study medication in doses in excess of that specified in the protocol is considered to be an overdose. Refer to section 10.2.1.1 for procedures in case of an overdose.

If the patient vomits within 30 minutes from taking the tablet or if they can identify the tablet in the vomit content, the patient can take a new tablet from the bottle. Trial medication should continue as usual on the subsequent day.

7.3.1.1 Difficulties swallowing tablets

Entry into the DORIC trial requires that a patient is able to swallow tablets whole. However, for patients who are unable to swallow tablets due to their underlying disease or who become unable to swallow the tablet whole whilst on study, and who are considered to be benefiting from therapy, tablets may be administered by alternative methods as outlined below.

Cediranib

From the Cediranib IB (dated 08.11.2010).
Cediranib film-coated tablets have not been deliberately formulated to be dispersible tablets. However they may be administered as a dispersion in water (ie, either drinking water, sterile water [for

injection] or purified water). Liquids other than water should not be used, and the tablets should not be crushed or ground. Care should be taken to ensure that the whole dose is administered.

The following procedure is recommended:
Cediranib tablets may be dispersed in half a glass (2 fluid ounces or 50 ml) of non-carbonated drinking water. No other liquids should be used. The tablet is dropped in water, without crushing, stirred until dispersed (approximately 10 minutes) and the resultant dispersion swallowed immediately. Any residues in the glass are mixed with half a glass of water and swallowed. The liquid can also be administered through nasogastric or gastrostomy tubes.

Where patients require the dose to be administered by nasogastric or gastrostomy tubes, cediranib tablet(s) dispersed in water can be dosed with:

- Polyurethane (PUR) or Poly vinylchloride (PVC) naso-gastric, naso-intestinal or percutaneous endoscopic gastrostomy (PEG) feeding systems in conjunction with PUR syringes.
- PVC syringes are not recommended for use.
- Two system washes are conducted through the giving set to ensure the correct dose is obtained.

Gefitinib

From the Iressa SmPC (dated 16.07.2009).
The tablet can be swallowed whole with some water or if dosing of whole tablets is not possible, tablets may be administered as a dispersion in water (non carbonated). No other liquids should be used. Without crushing it, the tablet should be dropped in half a glass of drinking water. The glass should be swirled occasionally, until the tablet is dispersed (this may take up to 20 minutes). The dispersion should be drunk immediately after dispersion is complete (i.e. within 60 minutes). The glass should be rinsed with half a glass of water, which should also be drunk. The dispersion can also be administered through a naso gastric or gastrostomy tube.

7.3.2 Pharmacy responsibilities:

Labelling

The clinical study drugs (cediranib and gefitinib and gefitinib placebo) will be supplied by AstraZeneca Investigational Products and shipped by Fisher. Cediranib and gefitinib and gefitinib placebo will be

packed into white high-density polyethylene bottles with child resistant, tamper evident closures. Each container of cediranib, gefitinib or placebo will have an investigational use label permanently affixed to the outside and will be labelled in accordance with local regulations, stating that the drug is for investigational use only and should be kept out of reach of children. Instructions stating that the tablets should be taken at the same time each day will be included.

It is the investigator's responsibility to ensure the patient receives the correct dose.

All products distributed through the Interactive Web Response System (IWRS) system will be labelled with a unique medication ID number to allow drug tracking.

Storage

All investigational products must be kept in a secure place under appropriate storage conditions. A description of the appropriate storage and shipment conditions are specified on the investigational product bottle label and in the Investigator's Brochure for cediranib (AZD2171, 2010) and SmPC for gefitinib. The investigator will instruct the patient about storage requirements for study medication. In the event of deviations from acceptable storage temperatures during the storage of trial drug at the site, the temperature excursions procedure should be followed (details will be held in the pharmacy file).

Accountability

It is the investigator/institution's responsibility to establish a system for handling study treatments, including investigational medicinal products, so as to ensure that:

- Deliveries of such products from Fisher are correctly received by a responsible person.
- Such deliveries are recorded.
- Study treatments are handled and stored safely and properly.
- Study treatments are only dispensed to study patients in accordance with the protocol.
- Any unused products are accounted for and returned for destruction, or destroyed locally, in liaison with UCL CTC
- The study personnel will account for all drugs dispensed.
- Certificates of delivery, destruction and return must be signed

Destruction of these drugs will be conducted according to a site's local policy, at the request of AstraZeneca and/or the UCL CTC. Drug destruction logs must be maintained and returned to the UCL CTC as requested.

7.3.3 Drug accountability

Accountability for cediranib, gefitinib and placebo at participating sites is the responsibility of the Principal Investigator, who may delegate this responsibility to the local pharmacist, or other appropriately qualified personnel. The responsible person will ensure that the cediranib, gefitinib and placebo are used only in accordance with this protocol and that appropriate records are maintained.

The site pharmacy must maintain accountability records for each drug including receipt, dispensing, return and destruction of medication. Accountability logs will be provided for each site pharmacy and accountability will be carried out throughout the trial whilst patients receive study medication. Drug delivery receipts, certificates of destruction of unused/expired medication and records of returned unused/expired medication must be kept in the pharmacy file. Temperature logs should be kept in the pharmacy file or centrally.

Patients should return any unused medication at their next visit to the pharmacy (e.g when collecting their next batch of trial medication). Returns should be counted by the pharmacist and recorded on accountability logs in the trial pharmacy site files. Patients who withdraw from the trial should arrange for medication to be returned to the pharmacy for accountability and destruction in cytotoxic waste according to local procedures.

Cediranib, gefitinib and placebo are supplied for DORIC patients only and must not be used outside the context of this protocol. Under no circumstances should the P.I. or other site personnel supply cediranib, gefitinib or placebo to other investigators, patients, or clinics, or allow supplies to be used other than directed by this protocol without prior authorisation from the supplier and notification to the Sponsor.

7.3.4 Dose modifications

If cediranib or gefitinib is not tolerated, patients will be managed by allowing a dose reduction by one or more levels according to Table 5.

Table 5 Dose reduction levels for cediranib and gefitinib

Dose Reduction level	Cediranib
0	30mg/od
-1	20mg/od
-2	15mg/od
-3	Stop drug

Dose Reduction level	Gefitinib / placebo
0	500mg/od
-1	250mg/od
-2	Stop drug

The dose reduction schedule for specific toxicities are described in Section 7.4.1 (Cediranib-related), Section 7.4.2 (Iressa-related) and Section 7.4.3 (related to both agents).

7.4 Management of general toxicity

The following general guidance is recommended for management of toxicities and dose de-escalation.

- All dose changes should be documented with clear reasoning and documentation of the approach taken
- Treat each of the toxicities with maximum supportive care (including withholding the experimental therapy where required)
- If the symptoms promptly resolve with supportive care, consideration should be given to continuing the same dose of study medications along with appropriate continuing supportive care, providing that continued treatment with study therapy is considered medically appropriate, in the opinion of the investigator as the patient is considered to be receiving benefit. If medically appropriate, dose reductions are permitted for study medications

Prophylatic antiemetics need only be given as required.

7.4.1 Management of toxicity attributable to cediranib

Dose interruptions should be used as the first approach to managing toxicity and dose reduction may be considered.

The events for which de-escalation of the cediranib dose is recommended include, with the EXCEPTION of hypertension:

- CTCAE grade 3 or higher toxicities of duration >3 days that are considered to be related to study treatment that are not responding to maximal supportive care within 48 hours, at the discretion of the investigator.

With the EXCEPTION of

- proteinuria (see Section 7.4.1.1),
- hypothyroidism (see Section 7.4.1.2)
- hypertension (see Section 7.4.1.4), and
- reversible posterior leucoencephalopathy syndrome (RPLS) (see Section 7.4.1.7),

the following management plan should be followed for management of toxicity attributable to cediranib:

Dose interruptions should be used as the first approach to managing toxicity. For CTC Grade 3 or more, dosing with cediranib should be interrupted**.** Cediranib dosing may be withheld for up to 14 days (21 days in consultation with the CI) for management of toxicity. If a longer interruption is required due to unresolved toxicity, cediranib should be discontinued.

- If the symptoms promptly resolve to grade 1 or below with supportive care, consideration should be given to continuing the same dose of cediranib along with appropriate continuing supportive care

- If the symptoms of the toxicity are considered related to cediranib and do not resolve to grade 1 or below with maximum supportive care and following a dose interruption of up to 14 days (21 days in consultation with the CI), the next dose level of cediranib below that being dosed should be instituted

- If symptoms do not resolve, and it is considered medically appropriate, investigators may choose to permanently discontinue cediranib

For patients who have multiple low grade adverse events (for example, diarrhoea, weight loss, dehydration and fatigue) short dose interruptions (ie, 2 - 5 days) of cediranib tablets may help. Treatment can be restarted on resolution at the same dose.

Two dose reductions for the cediranib treatment will be permitted during the study. No re-escalation of dose is permitted.

A maximum of a 14-day (21-day in consultation with the CI) delay in dosing for cediranib is permitted. If a longer interruption is required due to unresolved toxicity, cediranib should be discontinued. In addition, there are certain circumstances in which study medication should be permanently discontinued (see section 13.1).

7.4.1.1 Management of proteinuria

If a patient has a change of two plus (++) from baseline on two consecutive urine protein dipstick measurements, please measure urine protein creatinine ratio or collect a 24-hour urine for total protein. A urine protein/creatinine ratio of 0.15 (urine protein and urine creatinine expressed in mg/dL) approximates a 24-hour urine protein of 150 mg/24 hours or 0.15g/24 hours, which is the upper limit of normal. If 24 hour proteinurea or UPC ratio is classified as CTCAE grade 3 or more please follow guidance in Section 7.4.1.

Table 6. Approximation of the urinary protein / creatinine ratio to the CTC grading of proteinuria (from CTCAE v4.03:

Assessment (for Proteinuria)	CTC Grade 1	CTC Grade 2	CTC Grade 3	CTC Grade 4	CTC Grade 5
Dipstick	1+	2+			
24-hour urine protein	<1.0 g/24 hrs	>1.0 – 3.4 g/24 hrs	>3.5 g/24 hrs		
Urinary protein / creatinine ratio		0.5-1.9	>1.9		

Note: For UPC Ratio, obtain first morning void samples if possible and obtain at least 2 baseline samples. Urine protein and urine creatinine expressed in mg/dL.

7.4.1.2 Management of abnormal thyroid function tests

Cediranib therapy has been associated with increases in TSH. In the majority of patients this has not resulted in reductions in either total triiodothyronine (T3) or free thyroxine (T4) to below the lower limit of the normal range, but clinical hypothyroidism has been reported in a small number of patients. Patients have responded to replacement therapy without the need for stopping or reducing the dose of cediranib.

Pharmacological levothyroxine substitution should be given when clinically indicated to normalise the thyroxine level to within the normal range, and before the patient becomes clinically symptomatic. Levothyroxine therapy should also be considered in patients with TSH increases (and thyroxine levels within the normal range), with adverse events and symptoms suggestive of hypothyroidism such as fatigue. Thyroid function and related symptoms should be monitored frequently and the dose of levothyroxine should be titrated as required (Rini et al. 2007).

7.4.1.3 Management of haematological toxicity

Thrombocytopenia, of CTC grade 1 or 2 in the majority of cases, has also been seen with cediranib monotherapy and combination therapy with other treatments. In view of this finding, close monitoring of platelets at study visits is recommended. Patients with platelet count ≤100 x 109/L at baseline should not be included in studies with cediranib and are not eligible for this trial.

Although the effect of short treatment breaks of cediranib on platelet count recovery has not been studied, it is possible that a short break of a few days may help in the situation of CTC grade 3 or grade 4 or prolonged thrombocytopenia. Treatment breaks should be taken at the discretion of the treating clinician and patient response monitored. As guidance, where platelets are $<50 \times 10^9$/L a dose reduction or interruption may be required. Concerns may be discussed with the Chief Investigator.

7.4.1.4 Hypertension management

Patients will be provided with blood pressure monitors at the beginning of the trial to monitor blood pressure at home on a daily basis and will be encouraged to record this in the patient diaries. Data from the diaries will be used by the site staff to complete the CRFs at each assessment.

Table 7 Hypertension management protocol for emergent hypertension

Hypertension severity	Actions
**Mild to moderate hypertension:**	1. Repeat reading at least 1 hour later. If isolated increase, increase BP monitoring to weekly by health professional or daily home monitoring. Continue all study medication at the same dose.
BP 140/90 mmHg on 2 consecutive occasions >24 hours apart	2. If confirmed by second reading, continue all study medication at the same dose and initiate monotherapy with a long acting dihydropyridine calcium-channel blocker (e.g. nifedipine, amlodipine, felodipine) at low dose.
	3. If calcium channel blocker is contraindicated, use thiazide diuretic or angiotensin converting enzyme inhibitors or angiotensin receptor blockers first line.
OR	4. Monitor BP 3 weekly by health professional until it stabilises.
Increase in diastolic pressure by ≥20 mmHg or to ≥100 mmHg or increase in systolic pressure to ≥150 mmHg	5. If BP >140/90 mmHg after 3 weeks INCREASE the first agent to the full dose and consider adding an additional agent in combination (e.g., selective β-blocker, thiazide diuretic, angiotensin converting enzyme inhibitors or angiotensin receptor blockers).
	6. If BP >140/90 mmHg after a further 3 weeks, add an additional agent if the patient is only on one new agent or increase to full doses of the 2-drug combination.
	7. If BP >160/95 mmHg and is static or increasing after a further 7 days, **temporarily stop cediranib** and continue anti-hypertensive therapy under close supervision.
	8. Restart cediranib at the same dose (with maintenance anti-hypertensive therapy) when BP ≤140/90 mmHg. Monitor BP every week until BP stabilised.
	9. If BP >160/95 mmHg and is static or increasing after 7 days, **temporarily stop cediranib** and continue anti-hypertensive therapy under close supervision.
	10. Restart cediranib but at 1 dose lower than the starting dose when BP ≤140/90 mmHg. Monitor BP every week by health professional until BP stabilised.
	11. If BP increases to >160/95 mmHg, temporarily stop cediranib and continue anti-hypertensive therapy under close supervision
	12. Restart cediranib but at 2 doses lower than the starting dose when BP<140/90 mmHg. Monitor BP every week until steady state is BP stabilised
	13. If BP increases to >160/95 mmHg after dose reduction to 15 mg od, despite anti-hypertensive therapy, permanently stop cediranib.

Hypertension severity	Actions
**Severe hypertension:**<br><br>Increase in diastolic pressure to ≥110 mmHg or increase in systolic pressure to ≥180 mmHg on 2 readings >1 hour apart	1. Temporarily stop cediranib and consider if hospitalisation is necessary.<br><br>2. Initiate treatment with a 2-drug combination including a calcium channel blocker licensed for use in severe hypertension, tailored to the patient's underlying conditions and previous anti-hypertension treatment.<br><br>3. If there is evidence of target-organ damage, intravenous therapy should be considered while continuing oral therapy.<br><br>4. Nitrates may adversely affect the therapeutic mechanism of action of cediranib but should be used if clinically indicated.<br><br>5. Restart cediranib at 1 dose lower than the starting dose when BP <140/90 mmHg. Monitor BP every 3 to 7 days until steady state is BP stabilised<br><br>6. If BP increases to >160/95 mmHg, temporarily stop cediranib and continue anti-hypertensive therapy under close supervision.<br><br>7. Restart cediranib at 2 doses lower than the starting dose when BP <140/90 mmHg. Monitor BP every week until steady state is BP stabilised.<br><br>8. If BP increases to >160/95 mmHg after dose reduction to 15 mg od, despite anti-hypertensive therapy, permanently stop cediranib.

The development of hypertension in patients treated with cediranib is generally an early event, usually occurring in the first 4 weeks of treatment. In a minority of patients hypertension can also occur later in a treatment schedule.

The rigorous monitoring of BP and adherence to the hypertension management guidelines are necessary in order to achieve optimal hypertension control. Patients on prior anti-hypertension therapy may be particularly at risk of developing moderate or severe hypertension on cediranib. Therefore, patients with pre-existing hypertension or on anti-hypertension medications are likely to benefit from having their BP management optimised before starting cediranib.

For all BP thresholds described in this protocol, a trigger level is considered to be met if either the systolic and/or diastolic pressure reaches the threshold. If the threshold is recorded at home, it must be confirmed by a healthcare professional as defined above before commencing any treatment. CLEAR reasons for progressing to the next step in the management protocol must be documented.
When managing mild to moderate hypertension, the following principles should be noted:

- Cediranib may cause rapid increases in BP in some patients
- Calcium-channel antagonists are the first line agents of choice
- Increase anti-hypertensives to maximum doses and add additional agents as required.
- It is recommended that no more than 2 drugs are added in a 48-hour period before temporarily stopping cediranib.

The following cautions and contraindications should be noted:

- Calcium-channel blockers: use with caution in patients with tachyarrhythmias, aortic stenosis, unstable angina or congestive cardiac failure and may cause headache
- Short-acting non-dihydropyridines such as diltiazem or verapamil should be avoided since they may precipitate abrupt fall in BP and increase risk of myocardial ischaemia, infarction or stroke
- Beta-blockers: contraindicated in patients with asthma, chronic obstructive pulmonary disease and A-V block; they should be used with caution in patients with peripheral vascular disease and glucose intolerance and may cause fatigue
- If diuretics are to be used, thiazides rather than loop diuretics are recommended

A record of the management of hypertension will be maintained.

CTCAE grade 3 should NOT be assigned to hypertension AEs on the basis of the number of drugs used according to this protocol to treat mild-moderate hypertension since this is a proactive treatment approach. CTCAE grade 3 should be assigned **if hypertension is not controlled after 7 days of per-protocol anti-hypertensive therapy.**

7.4.1.5 Dermatological toxicity

The cumulative exposure of trial subjects to oral VEGFR tyrosine kinase inhibitors is associated with the dose dependent development of palmar plantar erythrodysesthesia (PPE) (Porta et al. 2007; Rosenbaum et al. 2008). Patients with PPE develop a painful blistering condition of the acral surfaces that is associated with focal areas of desquamation and callus formation. Xerosis (dryness) and erythema are often prominent features within the surrounding skin. The most commonly involved surfaces are those that are particularly subject to the forces of friction and pressure (palms, digits and soles of feet). In the most severe cases of PPE patients may require breaks in treatment and sometimes dose reductions.

General measures for managing PPE associated with oral VEGFR tyrosine kinase inhibitors (Schwandt A et al. 2009):

- Patients should be advised to make use of the following: cotton socks, shoes with extra support and gel insoles
- Topical emollient creams and lotions should be used to minimise dryness, pruritus and discomfort
- The use of oral anti-histamines can also be considered for the symptomatic management of pruritus
- The use of urea containing creams can be considered for the management of areas affected by hyperkeratosis

See Appendix 1 for details of recommended skin care products.

7.4.1.6 Oral toxicity

Mucositis may also occur as a schedule limiting toxicity in patients receiving cediranib.

General supportive measures to prevent/ minimise oral toxicity include the following:

- The gentle brushing of teeth and the use of non-alcohol based mouthwashes and toothpastes
- Symptomatic relief from chelitis and mucositis may be provided by the use of lip balms and lignocaine containing mouth washes

See Appendix 2 for details of recommended oral care products

7.4.1.7 Reversible posterior leukoencephalopathy syndrome

Reversible posterior leukoencephalopathy syndrome (RPLS) is a rare syndrome affecting vascular endothelial cells in the brain that may lead to capillary leak and oedema, and was first described in 1996 (Hinchey et al. 1996). It has been associated with a number of conditions, including renal failure, hypertension, fluid retention, and the use of cytotoxic or immunosuppressive drugs. It has also been reported rarely in association with the use of VEGF inhibitors including bevacizumab and sunitinib. Cases of Magnetic Resonance Imaging (MRI)-documented RPLS have rarely been reported in patients receiving cediranib in clinical studies.

The syndrome can present in a variety of non-specific ways, including headache, seizures, lethargy, confusion, blindness and other visual and neurological disturbances. Hypertension may be present, but is not necessary for the diagnosis of RPLS. MRI is the most sensitive imaging modality to detect RPLS and is recommended in suspected cases to confirm the diagnosis. RPLS is reversible upon removal of any possible precipitating factors and control of hypertension.

Active management of hypertension according to the hypertension management guidelines in this section may be expected to reduce the incidence of RPLS. However, if any case of RPLS occurs that is confirmed by imaging (CT or MRI), cediranib should be immediately discontinued in addition to any other measures to alleviate symptoms and control blood pressure.

7.4.1.8 Fatigue

Fatigue experienced by patients taking cediranib and/or gefitinib may be rapid in onset. During appointments patients should be specifically interrogated about fatigue, and estimated fatigue levels (according to CTC) should be recorded.

Patients should seek medical advice early if Grade 2 fatigue develops (moderate fatigue causing difficulty performing some activities of daily living).

Fatigue management

- In case of clinically significant fatigue in the investigators assessment, the patient may consider short treatment breaks (cediranib and gefinitib) of 2-3 days (max. 14 days allowed (21 days in consultation with the CI)). Care should be taken to ensure that the patients' nutritional status is optimised.
- Patients should be encouraged to drink plenty of fluids (1.5L/day- sips).
- Consideration should also be given to other causes e.g. depression, insomnia, thyroid function (see section 7.4.1.2), diarrhoea/dehydration, anaemia, drug use e.g. CNS depressants (antiepileptics) and anxiolytics or adrenal function.
- Patients should be encouraged to manage fatigue by alternating periods of rest with frequent light exercise, which may improve the symptoms in some cases.
- Patients should restart treatment when symptoms have improved.

7.4.2 Management of toxicity attributable to gefitinib

Dose interruptions should be used as the first approach to managing toxicity and dose reduction may be considered.

The events for which de-escalation of the gefitinib dose is recommended include:

- CTCAE grade 3 or higher toxicities of duration >3 days that are considered to be related to study treatment that are not responding to maximal supportive care within 48 hours, at the discretion of the investigator.

With the EXCEPTION of

- Rash (section 7.4.2.1)
- Interstitial lung disease (section 7.4.2.2)
- Diarrhoea (section 7.4.3)

the following management plan should be followed for management of toxicity attributable to gefitinib:

Dose interruptions should be used as the first approach to managing toxicity. For CTC Grade 3 or more, dosing with gefitinib should be interrupted**.** Gefitinib dosing may be withheld for up to 14 days (or 21 days in consultation with the CI) for management of toxicity. If a longer interruption is required due to unresolved toxicity, gefitinib should be discontinued.

- If the symptoms promptly resolve to grade 1 or below with supportive care, consideration should be given to continuing the same dose of gefitinib along with appropriate continuing supportive care

- If the symptoms of the toxicity are considered related to gefitinib and do not resolve to grade 1 or below with maximum supportive care and following a dose interruption of up to 14 days (or 21 days in consultation with the CI), the next dose level of gefitinib below that being dosed should be instituted

- If the symptoms do not resolve and it is considered medically appropriate, investigators may choose to permanently discontinue gefitinib

For patients who have multiple low-grade adverse events short dose interruptions (ie, 2- 5 days) of the gefitinib tablets may help. Treatment can be restarted on resolution at the same dose

One dose reduction for the gefitinib treatment will be permitted during the study. No re-escalation of dose is permitted.

A maximum of a 14-day (21-day in consultation with the CI) delay in dosing for gefitinib is permitted. If a longer interruption is required due to unresolved toxicity, gefitinib should be discontinued.
In addition, there are certain circumstances in which study medication should be permanently discontinued (see section 13.1).

7.4.2.1 Skin toxicity

Skin rash is a commonly reported outcome of treatment with gefitinib (Herbst et al. 2003; Wacker et al. 2007). Typically the rash comprises an acneiform dermatitis but also includes xerosis (dry skin), hyperpigmentation, telangiectasia, nail and hair changes. Gefitinib should be interrupted or discontinued in patients that develop severe bullous, blistering or exfoliating conditions. It is recommended that patients use sunscreen and limit sun exposure while receiving gefitinib as sunlight can exacerbate any skin reactions that may occur. See Appendix 1 for a list of recommended skin care products.

Table 8. Suggested dose adjustments for rash

Adverse Event Grade	Dose modification	Management Guideline
**Grade A**	None	The following agents have been used to treat rash with varying results:<br>Alcohol-free emollient cream<br>Chlorphenamine<br>Topical corticosteroids<br><br>Topical drying agents are not recommended
**Grade B**	None	
**Grade C**	Dose reduction (optional *).	
**Grade D**	Dose reduction as for Grade C or discontinue (optional)	

*Dose reductions for rash are not required and should only be considered if the rash is causing distress or discomfort to the patient.

Scale for describing gefitinib induced acneiform eruption

Grade
A = erythema
B = erythema with papules
C = erythema with papules and pustules
D = erythema with papules and confluent pustules

7.4.2.2 Interstitial Lung Disease

The emerging safety profile of the gefitinib, in non-small cell lung cancer patients, suggests an overall treatment related incidence of Interstitial Lung Disease (ILD) of 1% (Cohen et al. 2003). Almost a third of the cases of ILD have been associated with fatal outcomes. The reported incidence of gefitinib associated ILD has been around 2% in the post-marketing experience in Japan, around 0.3% in approximately 23,000 patients treated with gefitinib in a US expanded access program and about 1% in studies utilising gefitinib in the first-line treatment of NSCLC (with similar rates of ILD in both treatment and placebo groups). In the Iressa Pan Asia (IPASS) study in NSCLC, 2.6% of the gefitinib arm suffered ILD compared to 1.4% of the comparator arm (carboplatin/paclitaxel).

Patients with concurrent idiopathic pulmonary fibrosis whose condition worsens while receiving gefitinib have been observed to have an increased mortality compared to those without concurrent idiopathic pulmonary fibrosis (Naito et al. 2008).

Patients typically present with acute dyspnoea that progresses relatively rapidly and invariably requires inpatient admission. In some instances patients develop associated symptoms of cough and low-grade fever.

Chest radiographs are found to be normal in 10% of patients with ILD and therefore there should be a low threshold for the further investigation of the following:

- Patients with symptoms of progressive breathlessness, exertion (dyspnoea) +/- a persistent non-productive cough.
- Patients with an abnormal chest radiograph
- Patients who have or develop a restrictive ventilatory pattern of lung function abnormalities

Treatment with gefitinib should be ceased in all patients with a suspected diagnosis of ILD. High resolution computed tomography (HRCT) scanning is suggested as the most appropriate investigation to follow routine chest radiography. Providing that the respective patient is sufficiently stable then formal pulmonary function testing (including spirometry and gas transfer measurements) should be performed. High-dose dexamethasone therapy can be initiated at the discretion of the treating physician (Camus et al. 2004; Seto et al. 2006).

7.4.3 Diarrhoea

Both cediranib and gefitinib are associated with diarrhoea and therefore action should be taken to manage diarrhoea as soon as symptoms develop.

The recommendations for diarrhoea management in this study are based on guidelines from the American Society of Clinical Oncology. The guidelines suggest the aggressive management of ‘treatment induced’ diarrhoea to ensure that complications are avoided and that further treatments can be administered without delays (Benson et al. 2004).

Table 9. Assessment of diarrhoea according to CTCAE version 4.03

CTCAE v4.03 Grade	Patient without stoma	Patient with stoma
1	Increase of <4 stools per day over baseline	mild increase in ostomy output compared to baseline
2	Increase of 4 - 6 stools per day over baseline	Moderate increase in ostomy output compared to baseline
3	Increase of ≥7 stools per day over baseline; incontinence; hospitalization indicated	severe increase in ostomy output compared to baseline; limiting self care ADL
4	Life-threatening consequences; urgent intervention indicated	Life-threatening consequences; urgent intervention indicated
5	Death	Death

7.4.3.1 Initial management of diarrhoea

- Patients should be made aware that they are likely to experience diarrhoea
- Patients should be given loperamide to take home with them
- If diarrhoea occurs patients should immediately start loperamide after the first episode (4mg initially then 2mg every loose stool) and continue to take it until they have been free from diarrhoea for at least 12 hrs (up to a maximum of 16mg/day)
- Patients should be encouraged to drink plenty of fluids. Electrolyte replacements (e.g dioralyte) are recommended.

Patients should seek advice early, from their physician or study nurse, if

- Grade 1 or 2 diarrhoea persists for over 24hrs despite treatment with loperamide
- Grade 3 diarrhoea develops

- Any grade of diarrhoea associated with vomiting, marked abdominal distension or inability to take oral fluids develops

7.4.3.2 Management of persistent (>24h) or uncontrolled severe diarrhoea despite loperamide

- Patients should be advised to stop the cediranib and gefitinib study tablets and care should be taken to prevent dehydration.

- Following evaluation, consider ciprofloxacin for 7 days particularly if the patient has a fever or is neutropenic (although ciprofloxacin is a potential CYP450 inhibitor (see Appendix 3), in this instance it will be given whilst the patient is not on study medication).

- Consider infectious causes and aetiologies such as Clostridium difficile/viral gastroenteritis.

7.4.3.3 Management of persistent (>48h) diarrhoea despite loperamide

- The physician or study nurse should see patients in this situation.

- Hospitalisation and IV fluids may be needed.
- Consider infectious causes and aetiologies such as C-diff/viral gastroenteritis.
- Consider ciprofloxacin for 7 days particularly if the patient is neutropenic or has a fever.
- Octreotide (Sandostatin) may be considered**.**

7.4.3.4 Dose modifications for cediranib and gefitinib for diarrhoea

Diarrhoea which is Grade 1 in severity can be managed symptomatically without necessitating interruption of the trial drugs. Diarrhoea which is ≥ Grade 2 in severity will require the trial drugs to be interrupted and restarted when the diarrhoea resolves to Grade 1 or less, with or without a dose reduction as shown in Table 10. Dose reductions of cediranib and gefitinib will be made depending on the current dose of each agent according to the flowchart in Figure 1, giving preference to the reduction of cediranib prior to gefitinib.

Table 10. Guidelines for dose modification for diarrhoea (for grading see table 9)

**1st occurrence**	**Grade 1**	**Grade 2**	**Grade 3**	**Grade 4**
	Symptomatic Rx. Consider interruption of one or both trial drugs	Symptomatic Rx. Interrupt trial drugs, redose at same dose	Symptomatic Rx. Interrupt trial drugs, redose 1 step lower	Symptomatic Rx. Interrupt trial drugs, redose 2 or more steps lower
**2nd occurrence**	**Grade 1**	**Grade 2**	**Grade 3**	**Grade 4**
	Symptomatic Rx. Consider interruption of one or both trial drugs	Symptomatic Rx. Interrupt trial drugs. If previously: a) grade 1 redose at same dose b) ≥ grade 2 redose 1 step lower	Symptomatic Rx. Interrupt trial drugs. If previously: a) grade 1 redose 1 step lower b) grade 2 redose 1 step lower c) grade 3 redose at 1 or more steps lower	Symptomatic Rx. Interrupt trial drugs. If previously: a) grade 1,2 redose 2 or more steps lower b) grade 3,4 off study
**3rd occurrence**	**Grade 1**	**Grade 2**	**Grade 3**	**Grade 4**
	Symptomatic Rx. Consider interruption of one or both trial drugs	Symptomatic Rx. Interrupt trial drugs. If previously: a) grade 1 redose at same dose b) ≥ grade 2 redose 1 step lower	Symptomatic Rx. Interrupt trial drugs. If previously: a) grade 1 redose 1 step lower b) grade 2 redose 1 or more steps lower c) grade 3 redose at 1 or more steps lower	Symptomatic Rx. Interrupt trial drugs. If previously: a) grade 1,2 redose 2 or more steps lower b) grade 3,4 off study

Figure 1. Stepwise dose reductions to cediranib and gefitinib for diarrhoea

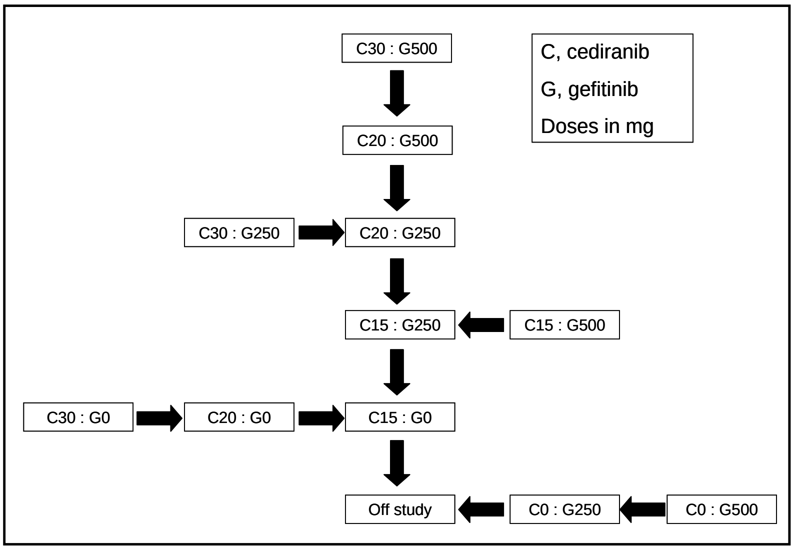

The cediranib should be re-introduced first and dose modifications made to find a tolerable dose before the gefitinib is re-introduced.

7.4.4 Renal Impairment

From the Iressa SmPC (dated 16.07.2009):

No dose adjustment is required in patients with impaired renal function at creatinine clearance >20ml/min. Only limited data are available in patients with creatinine clearance ≤20 ml/min and caution is advised in these patients.

No advice concerning dose adjustments in patients with impaired renal function is given in the Cediranib Investigators Brochure (dated 08.11.10).

For the purposes of this trial it is recommended that patients with a creatinine clearance of <30ml/min have a dose interruption for both trial drugs. Dose modifications could be considered depending on the cause of impairment.

7.4.5 Hepatic Impairment

Increases in transaminases, which are sometimes associated with increases in total bilirubin, have been seen in cediranib clinical studies.

Patients with ≥5x ULRR AST or ALT tests, or with ≥1.5 x ULRR serum bilirubin (except for patients with known documented cases of Gilbert's Syndrome) should dose interrupt both drugs and reintroduce at investigators discretion until cause established. Liver function tests should be monitored regularly (timing at investigators discretion). In the event of elevated bilirubin, direct and indirect bilirubin fractions should be measured.

Possible alternative causes for the abnormalities should also be investigated and documented, including concomitant medications, concomitant medical conditions, alcohol or IV drug abuse, blood transfusions, and recent travel abroad. As appropriate, tests may be performed to identify possible causes of the liver injury, such as liver ultrasound/computed tomography or MRI; serology and molecular virology; clotting screen and serum albumin; and autoantibody screening.

7.5 Concomitant medication

As discussed in section 5.1 Patient Eligibility, the following drugs are **not permitted** to be used concomitantly with study medication:

- Enzyme-inducing anti-epileptic drugs (from 2 weeks prior to study enrolment) (e.g carbamazepine, eslicarbazepine, oxcarbazepine, phenytoin, phenobarbital, primidone, topiramate, rifabutin)
- Other Investigational drugs (from 30 days prior to the first dose of cediranib/gefitinib)
- Other concomitant anti-cancer therapy except steroids (dexamethasone only)
- Other anti-angiogenesis (e.g. bevacizumab, sorafenib, sunitinib) therapy
- Other anti-EGFR treatments (e.g. cetuximab, panitumumab or small molecule TKIs etc.) or downstream targets mTOR inhibitors.

Caution should be exercised in the use of the following drugs, which are not recommended unless deemed essential for treatment:

- Medications which may markedly affect renal function (eg, vancomycin, amphotericin, ibuprofen, pentamidine)
- Any medication that may significantly affect hepatic P450 drug metabolising activity by way of enzyme induction or inhibition, or which require P450 for metabolism (from 2 weeks prior to the first dose of cediranib/gefitinib) (See Appendix 3 for a list)
- Medicinal products that cause significant sustained elevation in gastric pH, such as proton pump inhibitors and h2 antagonists.
- Antacids if taken regularly close in time to administration of gefitinib
- Oral anticoagulants (coumadin, warfarin) or low molecular weight heparin

Other medication, which is considered necessary for the patient's safety and well-being, may be given at the discretion of the investigator(s). The administration of all medication (including investigational products) must be recorded in the appropriate sections of the CRF.

7.6 Unblinding

7.6.1 Methods for ensuring blinding

The randomisation schedule that provides details of individual patient treatment will be produced by computer software that incorporates a standard procedure for generating random numbers. All study personnel will be unaware of the randomised treatment until all decisions regarding the integrity and evaluability of the data from all patients have been made and documented.

The gefitinib study medication will be provided as beige, round, film-coated tablets. The active and placebo tablets for each strength will be identical. Trial medication will be labelled using a unique material pack code which is linked to the randomisation procedure. Packaging, labelling and preparation of the study medication will be performed in a way that will ensure blinding throughout the study.

The treatment groups are double blind.

7.6.2 Methods for unblinding

The IWRS will hold information indicating the treatment randomisation for each randomised patient; this will be available to the investigator(s) or pharmacists via the website.

The treatment code must not be broken except in medical emergencies when the appropriate management of the patient necessitates knowledge of the treatment randomisation.

The investigator(s) must document and report to UCL CTC any breaking of the treatment code. UCL CTC retains the right to break the code for SAEs that are unexpected and are suspected to be causally related to an investigational product and that potentially require expedited reporting to regulatory authorities.

If an event causes concern, the dose of the trial drug should be reduced or discontinued according to this protocol, and the event treated.

Please note that once a patient has been unblinded, they will no longer have access to trial treatment.

Unblinding should occur following one of the two processes outlined below.

Unblinding Monday-Friday, 9am-5pm UK time

If a patient's randomised treatment requires unblinding during working hours i.e. 9am-5pm UK time Monday-Friday (but excluding UK bank holidays), the relevant site **must** contact the UCL CTC prior to any potential unblinding, and delegated UCL CTC trial staff will, where necessary, authorise the site to unblind the treatment arm using IWRS, following consultation with the Chief Investigator and/or clinical members of the Trial Management Group.

For any unblinding during working hours, individual treatment codes, indicating the treatment randomisation for each patient, will be available from the IWRS and accessible by the PI and/or site pharmacist, including email notification. Further procedures for these processes will be outlined in the IWRS user manual that will be provided to sites prior to trial activation.

Unblinding outside of working hours

If a patient's randomised treatment requires unblinding outside working hours i.e. outside 9am-5pm UK time Monday-Friday (and additionally UK bank holidays), sites are permitted to access the IWRS to unblind, **but** with the provisos outlined above **i.e. that unblinding must only be undertaken where knowledge of the randomised treatment is essential to patient safety/management.**

UCL CTC must be contacted BEFORE unblinding*:

DORIC Trial Coordinator
0207 679 9860
Doric@ctc.ucl.ac.uk
*except in cases of out-of-hours emergencies

Treatment codes will not be broken for the planned analyses of data until all decisions on the evaluability of the data from each individual patient have been made and documented. The treatment arms of patients with SARs thought to be unexpected for the trial medication (potential SUSARs) will be viewed in an unblinded fashion internally at UCL CTC where required for SUSAR reporting only, in these instances site staff will not be unblinded.

24 hour Emergency Medical Advice

A telephone number for the AstraZeneca switchboard, which will be open 24 hours a day, 365 days a year, will be listed on the Trials Contacts list in the investigator site file. Sites should use this number in the event that emergency medical advice is required in events that the investigator is unavailable or unable to advise.

7.7 Other trial treatments/interventions: Imaging

Central to the collection of the outcomes of this trial is regular MRI brain scans at screening/baseline and 6-weekly thereafter.

The same MRI scanner should be used for all images for the patient throughout the trial where possible. There should not be any switching between magnets of different field strengths.

The screening/baseline scan should be performed within 7 days prior to the randomisation date. This scan will be used to determine whether or not the patient is eligible for the trial. Routine scans prior to baseline should be assessed when deciding whether or not a patient should be screened for the trial, but will not be collected for this trial.

The follow up scans must be performed +/- 10 days of the due date (every 6 weeks after the start of trial treatment), irrespective of the trial treatment administration. Follow up scans should be consistent with baseline scans with regards to modality, scanner and all associated scanning parameters.

If a patient discontinues from the trial treatment, the discontinuation MRI should be completed within 10 days of discontinuation (if possible).

Patients must be on a stable dose of steroids (dexamethasone) for at least 5 days prior to baseline scan and at least 10 days prior to all follow-up scans (where possible).

The following images will be taken on a 1.5 or greater Tesla MR scanner:

IMAGE	Orientation	Slice gap (mm)	Slice thickness (mm)
FLAIR (Fluid-attenuated inversion-recovery image)	Axial	0mm	≤ 5mm
DWI (diffusion weighted image) Spin echo –echo planar	Axial	0mm	≤ 5mm
T2 (T2-weighted fast spin echo images)	Axial	0mm	≤ 5mm
T1 volumetric (three-dimensional gradient echo images)	Coronal	0mm	≤ 5mm
**Post-contrast** T1 volumetric (three-dimensional gradient echo images)	Coronal	0mm	≤ 5mm
**Post-contrast** FLAIR (Fluid-attenuated inversion-recovery image)	Axial	0mm	≤ 5mm

Table 11. Required MRI acquisition parameters

Post contrast images must be taken in the order stated above. Measurements for all scans on MRI will be in the plane where the tumour is largest. All images are required for the trial. The critical scans are FLAIR, T2, post-contrast T1.

If additional scans are performed according to local guidelines, these should be submitted for the trial central review in addition to the trial images.

Images should be reported locally for the follow up CRFs. Images should be sent to UCL CTC in DICOM format on CDs as soon as possible after acquisition (ideally also with a DICOM reader), along with a completed Acquisition report (see Appendix 8) and local imaging report (where available). Images should contain a reference to the date of the scan. Images must have patient names removed, and refer to patients via study number (in addition, initials, NHS number and date of birth are permitted but discouraged). The central review of MRI scans will be carried out retrospectively at the end of the trial. If there are problems accessing scan images during the review, sites may be contacted again to create a new CD.

Locally reported radiological response should be reported as defined in 16.4, with progression as defined in 2.6.1.1. Please note that there is a high possibility of pseudoresponse and pseudoprogression on MRI scans in patients receiving angiogenesis inhibitors. Where treatment decisions are based upon radiological response, a confirmatory second scan at the next 6 week visit should be used to make these decisions. Please note that patients who have radiologically detected progressive disease only (even if confirmed at the second scan) do not need to stop trial treatment if it is felt that they may be receiving benefit from this.

Recommended acquisition parameters for 3T Philips model are listed below for reference. Please use parameters which are the equivalent on your model of scanner:

IMAGE	Orientation	Pulse parameters			Flip Angle	Matrix	FOV	Slice gap (mm)	Slice thickness (mm)	No of acquisitions	TIME (min)
		TR (Repetition Time) / msec	TE (Echo time) / msec	TI (Inversion time) / msec							
FLAIR (Fluid-attenuated inversion-recovery image)	Axial	8000	125	2400	NA	240x180	240	0mm	3	1	3.4
DWI (diffusion weighted image) Spin echo –echo planar	Axial	2628	69		NA	192x113	230x 230	0mm	3	2	1
T2 (T2-weighted fast spin echo images)	Axial	3500	85		NA	240x180	240	0mm	3	1	4
T1 volumetric (three-dimensional gradient echo images)	Coronal	6.8	3.1		8°		256x 256	0mm	1	1	6.3
**Post-contrast** T1 volumetric (three-dimensional gradient echo images)	Coronal	6.8	3.1		8°		256x 256	0mm	1	1	6.3
**Post-contrast** FLAIR (Fluid-attenuated inversion-recovery image)	Axial	8000	125	2400	NA	240x180	240	0mm	3	1	3.4
**TOTAL**											24.4

Table 12. Suggested MRI acquisition parameters for 3T Philips model

8.0 Assessments

8.1 Schedule of Assessments

Visit	1	2 (start treatment)	3	4	5	6	7	8	9 onwards: 3 weekly Follow Up[k]		Discontinuation	Long term Follow Up
**Investigation**	**Screening**	**Baseline – Week 0**	**Week 1**	**Week 2**	**Week 3**	**Week 4**	**Week 5**	**Week 6**	**MID-CYCLE ASSESSMENTS** **Weeks 9, 15, 21, 27, 33, etc (every 3 weeks when not having a 6-weekly assessment, until discontinuation)**	**CYCLE START ASSESSMENTS** **Weeks 12, 18, 24, 30, 36 etc (Every 6 weeks until discontinuation)**	**Any time a patient Discontinues ALL study treatment due to progression or toxicity**	**After discontinuation, where possible[h]**
Visit window	Max 2 weeks before baseline[l]	Day 1 of Week 0	±3 days	±3 days	±3 days	±3 days	±3 days	±3 days	±3 days	±3 days	Day of discontinuation	Every 6 or 12 weeks[h]
CRFs to be completed	Consent forms, Registration CRF	Baseline CRF, AE form	Week 1 CRF, AE form, Con med form	Week 2 CRF, AE form	Week 3 CRF, AE form	Week 4 CRF, AE form	Week 5 CRF, AE form	Week 6 CRF, AE form, Con med form	Interim Assessment CRF, AE form, Con med form	6-Weekly Assessment CRF, AE form, Con med form	Withdrawal Assessment CRF, AE form, Con med form, Death/SAE form if relevant	Follow Up CRF, Death/SAE form if relevant
Timeframe for return of CRFs to UCL CTC	ASAP	Within 2 weeks	Within 1 month	Within 1 month	Within 1 month	Within 1 month	Within 1 month	Within 1 month	Within 1 month	Within 1 month	ASAP	Within 1 month
Informed consent	X											
Patient details	X											
Medical History	X											
Inclusion/exclusion criteria	X											
Physical Examination[a]	X	X	X	X	X	X	X	X		X	X	
Neurological Examination	X	X			X			X		X	X	X[h]
ECG[b]	X	X			X						X	
Clinical chemistry[b]	X	X			X			X		X	X	
Full blood count[b]	X	X	X	X	X	X	X	X	X	X	X	
Randomisation	X											

Visit	1	2 (start treatment)	3	4	5	6	7	8	9 onwards: 3 weekly Follow Up		Discontinuation	Long term Follow Up
**Investigation**	**Screening**	**Baseline – Week 0**	**Week 1**	**Week 2**	**Week 3**	**Week 4**	**Week 5**	**Week 6**	**MID CYCLE ASSESSMENTS**<br><br>**Weeks 9, 15, 21, 27, 33 etc (every 3 weeks when not having a 6-weekly assessment, until discontinuation)**	**CYCLE START ASSESSMENTS**<br><br>**Weeks 12, 18, 24, 30, 36 etc (Every 6 weeks until discontinuation)**	**Any time a patient Discontinues ALL study treatment due to progression or toxicity**	**After discontinuation, where possible[h]**
Clotting function tests[b]	X	X			X			X		X	X	
Thyroid function tests[b]	X	X			X			X		X	X	
Urinalysis[bc]	X	X			X			X		X	X	
EORTC QLQ-C30 plus BN-20 [d]		X						X		X (EVERY 3 MONTHS: WKS 18, 30, 42, 54, 66 ETC)	X	
Pregnancy tests (females of child-bearing potential) [e]	X											
MRI[f]	X							X		X	X	X
Karnofsky Performance Status	X	X			X			X		X	X	X
Mini-mental status exam	X							X				
Dispense gefitinib		X						X		X		
Dispense cediranib		X						X		X		
Adverse Events[g]	X	X	X	X	X	X	X	X	X	X	X[g]	X[g]
Dexamethasone dose per day	X	X	X	X	X	X	X	X	X	X	X	X[h]
Other Concomitant Medication	X	X			X			X		X	X	X[M]
Overall Survival			X	X	X	X	X	X	X	X	X	X
Biomarker samples (blood)[i]		X						X		X (WEEKS 12, 18, 24 ONLY)	X[j]	

8.2 Schedule of Assessments – further details

a) Physical Examination

Including height, weight, lying and 5 minute standing blood pressure, ECOG performance status. Patient's blood pressure needs to be monitored weekly for the first 6 weeks of treatment of cediranib +/- gefitinib. Patients/carers will monitor the patient's blood pressure daily and record this in the patient diaries. CRFs should reflect an average of the patient-reported daily values. If a patient has BP ≥150/100 mmHg at Screening, a further reading should be taken at least 24 hours later to assess eligibility. In event of hypertension, refer to hypertension management plan, see Section 7.4.1.4.

b) Baseline measurements

Lab, TSH and ECG measurements should be retested at baseline **only** if the screening period is greater than 7 days. Labs and ECGs must be evaluated within 7 days prior to randomisation to confirm eligibility.

c) Urinalysis tests

Urinalysis- if a patient has two consecutive one plus (+) urine protenuria dipsticks measurements at baseline, please measure urine protein/creatinine ratio (UPC) or collect 24 hours urine for total protein. If the patient has a change of two plus (++) or greater from baseline on two consecutive proteinuria dipsticks, please measure urine protein/creatinine ratio or collect 24 hours urine for total protein. If 24 hours protein or UPC Ratio is classified as CTCAE grade 3 please refer to and follow guidance in section 7.4.1.1, management of proteinuria.

d) Quality of life questionnaire timing

EORTC QLQ-C30 and BN-20 questionnaires should be completed before the patient receives any treatment and before given the results of their tumour assessments.

e) Pregnancy test

Women of child-bearing potential (see section 5.1.3) must have a negative urine or serum pregnancy test at the screening visit within 14 days prior to starting trial treatment.

f) MRI timing and guidance

Baseline contrast enhanced MRI must be completed within 7 days prior to randomisation according to acquisition guidelines. Follow-up scans should be performed at scheduled time points ± 10 days (every 6 weeks from the baseline date), independent of when the follow up assessment was completed or trial treatment administered. For acquisition guidelines see Section 7.7 (Imaging).

Patients must be on a stable dose of steroids (dexamethasone) for at least 5 days prior to baseline scan and at least 10 days prior to all follow-up scans. At discontinuation the MRI should be performed within 10 days of discontinuation where possible.

For patients who are withdrawn from study treatment for reasons other than disease progression every effort will be made to continue with collecting the trial MRI scans every 6 weeks.

g) Adverse event collection duration

Adverse events will be collected from the time informed consent is given, throughout the treatment period and up to and including the 30 days after last dose of study medication. AEs thought to be related to trial treatment will be followed up until resolution or death. Any ongoing SAE at discontinuation must be monitored until resolution. SAEs occurring after 30 days following the last dose of study drug do not need to be reported to UCL CTC unless thought to be related to the study treatment.

h) Follow up assessment schedule after discontinuation

If a patient discontinues due to reasons other than progression, the patient will continue to be followed-up every 6 weeks until progression or death. If a patient discontinues due to progression, or has progressed after discontinuation, the patient will continue to be followed-up every 12 weeks for survival (via telephone call). All efforts should be made by the Site to contact the patient's GP to assess their condition, if a patient fails to attend a clinic or cannot be followed up at site. The trial will be registered with the NHS Information Centre in the event that any patients become Lost to Follow Up.

Neurological status assessments should be continued until neurological deterioration has been documented on 2 consecutive 6 weekly assessments. Dexamethasone dose per day should be collected until progression documented (as outlined in section 2.6.1.1)

i) Blood samples for biomarkers

Samples must only be taken after provision of separate consent for genetic sampling and ideally prior to administration of investigational product. Blood samples taken at discontinuation should have a record of the time passed since last dose of trial treatment. See section 21 for details of samples.

j) Post-mortem samples

Post-mortem tumour samples should be arranged if the patient has discontinued due to death, and the deceased patient has given explicit consent for this. See section 21.

k) Assessment dates during trial treatment

The cycle start assessment should be carried out on the day the patient begins the next cycle of study treatment. **Clinic assessments and MRI assessments will occur at the specified time points irrespective of trial treatment holidays.** E.g a 6 week cycle may only contain 3 or 4 weeks of trial treatment if a patient has had a trial treatment holiday during that cycle. If either treatment is delayed for more than 14 days due to toxicity (or 21 days if the CI has permitted a 21 day treatment holiday), the patient should cease taking the medication and continue with the cycles of the other study treatment. If both the study treatments are delayed for more than 14 days due to toxicity (or 21 days if the CI has permitted a 21 day treatment holiday), the discontinuation assessment should be performed and the patient withdrawn from the trial.

l) Time between screening and baseline

A maximum of 14 days between screening and baseline is permitted. However for patients switching from enzyme inducing anti-epileptic drugs to non-enzyme inducing anti-epileptic drugs, this can be extended to a maximum of 21 days before baseline (as a wash-out period of 2 weeks is required. See 5.1.2 Exclusion criteria). Screening assessments must be performed within 7 days before randomisation, as per section 5.2 (pre-randomisation evaluation). **If more than 14 days pass between screening and baseline, the randomisation form must be updated with pre-randomisation tests completed within 14 days of the baseline visit and this reassessed for eligibility at UCL CTC before the patient proceeds with the baseline assessments.**

m) Concomitant medication collection after discontinuation

After discontinuation – concomitant medication for treatment of brain tumour only should be collected (e.g radiotherapy, chemotherapy, investigational drugs).

8.3 Patient diaries

Patients will be provided with a diary and encouraged to record daily blood pressure readings, compliance in taking study medication, and dexamethasone dose taken. These will be read by the site staff at assessment visits to assist with completing the CRFs. Patient diaries should be completed until progression is documented and returned to the site staff at each clinic visit.

8.4 Blood pressure monitors

Patients with be provided with a blood pressure monitor at the beginning of the trial (supplied to the site by either the Chief Investigator or the UCL CTC trial coordinator at site activation).

9.0 Data Management Guidelines

Data will be collected from sites on version controlled case report forms (CRFs) designed for the trial and supplied by UCL CTC. Data entered onto CRFs must reflect source data at site.

9.1 Completing Case Report Forms

All CRFs must be completed and signed by staff who are listed on the site staff delegation log and authorised by the PI to perform this duty. The PI is responsible for the accuracy of all data reported in the CRF.

Once completed the original CRFs must be sent to UCL CTC and a copy kept at site. All entries must be clear and legible. The use of abbreviations and acronyms must be avoided.

9.2 Corrections to CRFs

Any corrections made to a CRF at site must be made by drawing a single line through the incorrect item ensuring that the previous entry is not obscured. Each correction must be dated and initialled. Correction fluid must not be used. The amended CRF must be sent to UCL CTC and a copy retained at site.

9.3 Missing Data

To avoid the need for unnecessary data queries CRFs must be checked at site to ensure there are no blank fields before sending to UCL CTC. When data is unavailable because a measure has not been taken or test not performed, enter “ND” for not done. If an item was not required at the particular time the form relates to, enter “NA” for not applicable. When data are unknown enter the value “NK” (only use if every effort has been made to obtain the data).

9.4 Data Queries

Data arriving at UCL CTC will be checked for completeness, accuracy and consistency. Queries on incomplete, inaccurate or inconsistent data will be sent to the data contact at site. When responding to a query, site staff should attach an amended copy of the case report form held at site and send to

UCL CTC, keeping a copy at site. All amendments must be initialled and dated. All data which requires query will be queried at least once

9.5 Timelines for data return

Sites must complete and return CRFs to UCL CTC as soon as possible after patient visit and within one month of the patient being seen. The registration form should be faxed to the UCL CTC asap when completed to enable randomisation (see section 6.0). SAE reports should be sent via fax according to the timelines in section 10.2.2 (SAEs).

Sites who persistently do not return data within the required timelines may be suspended from recruiting further patients into the trial by UCL CTC.

10.0 Pharmacovigilance

10.1 Definitions of Adverse Events

The following definitions have been adapted from Directive 2001/20/EC, ICH E2A "Clinical Safety Data Management: Definitions and Standards for Expedited Reporting" and ICH GCP E6:

Adverse Event (AE)

Any untoward medical occurrence or effect in a patient treated on a trial protocol, which does not necessarily have a causal relationship with a trial treatment. An AE can therefore be any unfavourable and unintended sign (including an abnormal laboratory finding), symptom or disease temporally associated with the use of a trial treatment, whether or not related to that trial treatment.

Adverse Reaction (AR)

All untoward and unintended responses to a trial treatment related to any dose administered. A causal relationship between a trial treatment and an adverse event is at least a reasonable possibility, i.e. the relationship cannot be ruled out.

Serious Adverse Event (SAE) or Serious Adverse Reaction

An adverse event or adverse reaction that at any dose:

- Results in death
- Is life threatening (the term "life-threatening" refers to an event in which the patient was at risk of death at the time of the event. It does not refer to an event that hypothetically might have caused death if it were more severe)
- Requires in-patient hospitalisation or prolongs existing hospitalisation
- Results in persistent or significant disability/incapacity
- Is a congenital anomaly or birth defect
- Is otherwise medically significant (e.g. important medical events that may not be immediately life-threatening or result in death or hospitalisation but may jeopardise the patient or may require intervention to prevent one of the other outcomes listed above)

Suspected Unexpected Serious Adverse Reaction (SUSAR)

A serious adverse reaction, the nature or severity of which **is not consistent** with the applicable trial treatment information.

10.2 Reporting Procedures

10.2.1 All Adverse Events (AEs)

All adverse events that occur between informed consent and 30 days post last trial treatment administration must be recorded in the patient notes and the trial CRFs. Information regarding dates of event onset and resolution, outcome, severity and causality for the trial treatment must be recorded. AEs thought to be caused by trial treatment will be followed up until resolution or death.

Those meeting the definition of a Serious Adverse Event (SAE) must also be reported to UCL CTC using the trial specific SAE Report (see section 10.2.2 (Serious Adverse Events) for details).

Pre-existing conditions do not qualify as adverse events unless they worsen.

10.2.1.1 Overdoses

All accidental or intentional overdoses, whether they result in an adverse event or not, must be recorded in the patient notes and CRFs. Overdoses resulting in an adverse event are classified as SAEs and must be reported to UCL CTC according to SAE reporting procedures. 'Other medically significant event' must be ticked as the classification of serious and the fact that an overdose has occurred must be clearly stated. See also section 10.2.2 (Serious Adverse Events).

Sites must inform UCL CTC immediately when an overdose has been identified. See also section 11.0 (Incident Reporting and Serious Breaches).

10.2.1.2 Adverse Event Term

An adverse event term must be provided for each adverse event, preferably using the term as listed in the Common Terminology Criteria for Adverse Events v4.03 (CTCAE), available online at: http://evs.nci.nih.gov/ftp1/CTCAE/About.html

10.2.1.3 Severity

Severity of each adverse event must be determined by using the Common Terminology Criteria for Adverse Events v4.03 (CTCAE) as a guideline, wherever possible. The criteria are available online at: http://evs.nci.nih.gov/ftp1/CTCAE/About.html

In those cases where the CTCAE criteria do not apply, severity should be coded according to the following criteria:

1 = Mild (awareness of sign or symptom, but easily tolerated)
2 = Moderate (discomfort enough to cause interference with normal daily activities)
3 = Severe (inability to perform normal daily activities)
4 = Life threatening (immediate risk of death from the reaction as it occurred)
5 = Fatal (the event resulted in death)

10.2.1.4 Causality

The PI, or other delegated site investigator, must perform an evaluation of causality for each adverse event.

As this is a placebo-controlled trial, the evaluation of causality must be performed as if the patient is on active treatment.

Causal relationship to each trial treatment must be determined as follows:

- **None**
  There is no evidence of any causal relationship.
- **Unlikely**
  There is little evidence to suggest a causal relationship (e.g. because the event did not occur within a reasonable time after administration of a trial treatment). There is another reasonable explanation of the event (e.g. the patient's clinical condition, other concomitant treatments).
- **Possible**
  There is some evidence to suggest a causal relationship (e.g. because the event occurs within a reasonable time after administration of a trial treatment). However, the influence of other factors may have contributed to the event (e.g. the patient's clinical condition, other concomitant treatments).
- **Probable**
  There is evidence to suggest a causal relationship and the influence of other factors is unlikely.
- **Definitely**

There is clear evidence to suggest a causal relationship and other possible contributing factors can be ruled out.

10.2.2 Serious Adverse Events (SAEs)

All SAEs that occur between the signing of informed consent and 30 days post the last trial treatment administration (or after this date if the site investigator feels the event is related to the trial treatment) must be submitted to UCL CTC by fax within **1 business day** of observing or learning of the event, using the trial specific SAE Report. All sections on the SAE Report must be completed. If the event is **not being reported within 1 business day** to UCL CTC, the circumstances that led to this must be detailed in the SAE Report to avoid unnecessary queries.

10.2.2.1 Events which do not require immediate reporting on an SAE Report

The following events do not require immediate reporting on an SAE Report for this trial, but must be recorded in the relevant section(s) of the CRF:

- disease progression*
- disease related deaths*

* Investigators should take care to distinguish these from side effects of the trial drugs (e.g bleeds).

Please note that hospitalisation for elective treatment or palliative care, or problems with home carers, does not qualify as an SAE.

All SAEs must be reported by site staff by faxing a completed SAE Report within 1 business day of becoming aware of the event to UCL CTC
Fax: +44 (0)20 7679 9861

10.2.2.3 Adverse Event Reporting Flowchart

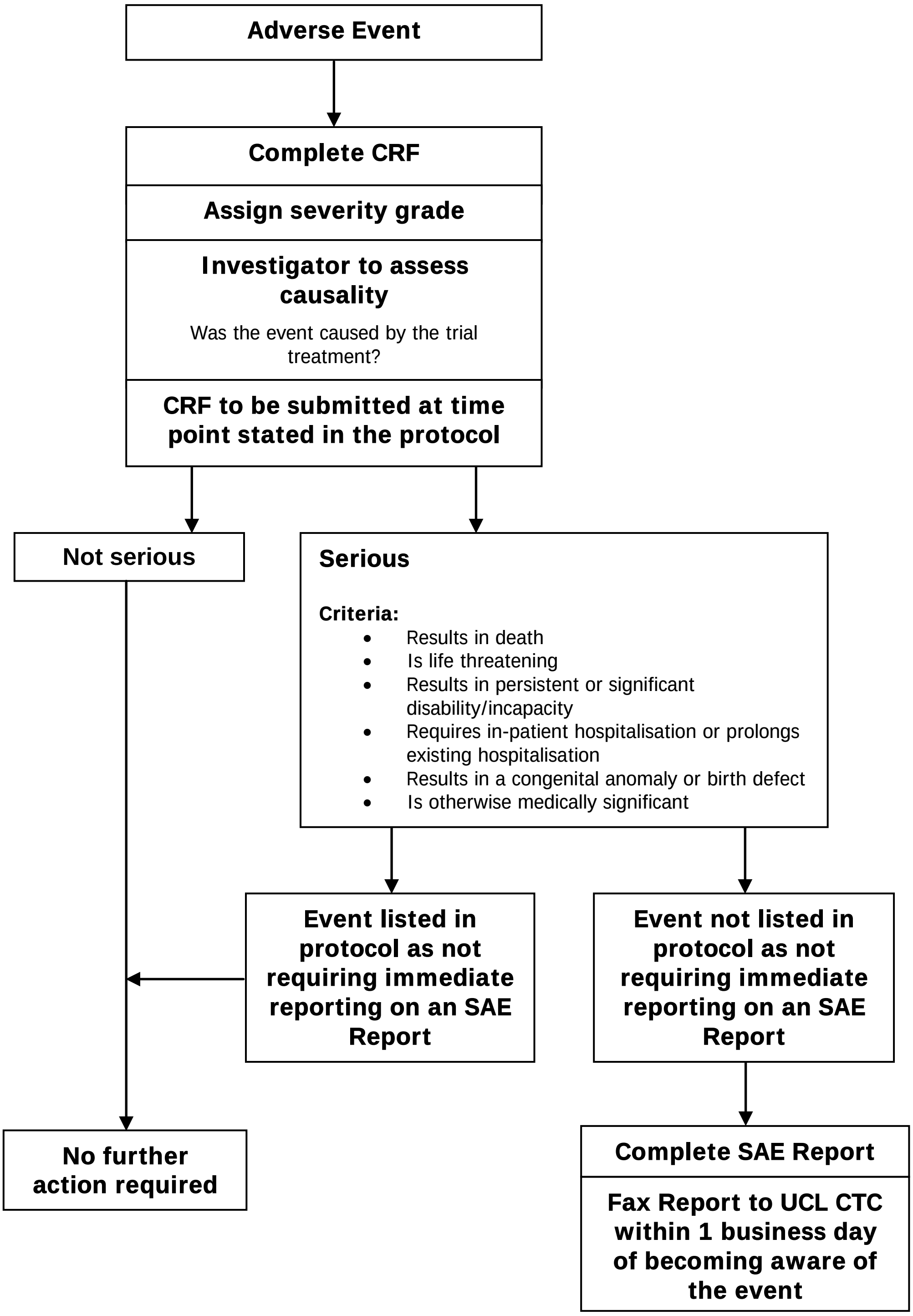

10.2.2.4 SAE Follow-Up Reports

All SAEs must be followed-up until resolution, and until there are no further queries. The PI, or other delegated site investigator, must provide follow-up SAE Reports if the SAE had not resolved at the time the initial report was submitted.

10.2.2.5 SAE Processing at UCL CTC

On receipt of the SAE Report, UCL CTC will check for legibility, completeness, accuracy and consistency. Expectedness will be evaluated, to determine whether or not the case qualifies for expedited reporting, using the list of expected adverse events in the current IB for cediranib and SmPC for gefitinib.

The CI, or their delegate (e.g. a clinical member of the TMG), will be contacted to review the SAE and to perform an evaluation of causality on behalf of UCL CTC. If UCL CTC has considered expectedness difficult to determine, the CI, or their delegate, will be consulted for their opinion at this time.

UCL CTC will send line-listings of all SAEs to AstraZeneca every 3 months.

10.2.2.6 Emergency unblinding of the treatment allocation

See section 7.6 for details.

10.3 SUSARs

If the event is evaluated by either the site or UCL CTC as a Suspected Unexpected Serious Adverse Reaction (SUSAR), UCL CTC will submit a report to the MHRA and REC within 7 calendar days for fatal/life threatening events, with a follow-up report within a further 8 calendar days, and 15 calendar days for all other events. In the case of conflicting evaluations of causal relationship by the site and the UCL CTC/CI, both opinions will be reported.

UCL CTC will unblind any SUSARs before expedited reporting, if not already unblinded. See section 7.6 (methods for unblinding) for details.

10.3.1 Informing Sites of SUSARs

UCL CTC will inform all PIs of all potential SUSARs that occur on the trial. PIs will receive a quarterly line listing which must be processed according to local requirements.

UCL CTC will forward reports regarding SUSARs that have occurred on other trials using cediranib to all PIs. These must be processed according to local requirements and filed with the IB.

10.4 Clinical Review

UCL CTC will provide safety information to the TMG and IDMC on a periodic basis for review. Should the outcome of the review result in upgrading/downgrading of SAEs to SUSARs and vice versa, UCL CTC will provide relevant reports to the MHRA and the REC.

10.5 Additional Safety Monitoring at UCL CTC

UCL CTC will also monitor safety data for any trial related events that are not considered related to the trial treatment. Should any trial procedures appear to be resulting in adverse events, the Trial Management Group will be contacted for their opinion. If it is declared necessary to review the conduct of the trial, UCL CTC will inform the MHRA and the REC as appropriate.

If UCL CTC detects a higher incidence in rare events than is stated in the IB/SmPC for the trial treatment, a report detailing the finding will be submitted to the MHRA and REC.

10.6 Pregnancy

If a female patient or a female partner of a male patient becomes pregnant at any point during the trial, a completed trial specific Pregnancy Report must be submitted to UCL CTC by fax within **1 business day** of learning of its occurrence. Consent to report information regarding the pregnancy must be obtained from the mother. The Pregnancy Monitoring Information Sheet and Consent Form for the partners of trial patients must be used for this purpose.

All pregnancies must be reported by site staff by faxing a completed Pregnancy Report within 1 business day of becoming aware of the pregnancy to UCL CTC
Fax: +44 (0)20 7679 9861

10.6.1 Pregnancy Follow-Up Reports

All pregnancies must be followed-up until an outcome is determined. Follow-up Pregnancy Reports must be submitted to UCL CTC by fax within **1 business day** of learning of the outcome. Reports must include an evaluation of the possible relationship of the trial treatment(s) to the pregnancy outcome.

10.6.2 SAEs During Pregnancy

Any SAE occurring in a pregnant patient must be reported using the trial specific SAE Report, according to SAE reporting procedures. See section 10.2.2 (Serious adverse events) for details.

10.6.3 Pregnancy Report Processing at UCL CTC

UCL CTC will submit Pregnancy Reports to the MHRA and the REC should the pregnancy outcome meet the definition of a SUSAR. See section 10.3 (SUSARs) for details.

10.7 Annual Safety Reports

UCL CTC will submit Annual Safety Reports to the MHRA and REC. This will commence one year from the date of CTA approval obtained for the trial. The final Annual Safety Report will be submitted in the year following trial closure. See section 14.1 (End of Trial) for details.

11.0 Incident Reporting and Serious Breaches

11.1 Incident Reporting

Organisations must notify UCL CTC of all deviations from the protocol or GCP immediately. UCL CTC may require a report on the incident(s) and a form will be provided if the organisation does not have an appropriate document (e.g. Trust Incident Form for UK sites).

UCL CTC will assess all incidents to check if they meet the definition of a serious breach.

11.2 Serious Breaches

Systematic or persistent non-compliance by a site with GCP and/or the protocol, including failure to report SAEs occurring on trial within the specified timeframe, may be deemed a serious breach.

In cases where a potential or actual serious breach has been identified, UCL CTC will inform the MHRA within 7 calendar days of becoming aware of the breach.

Sites must have written procedures for notifying the sponsor of serious breaches (MHRA Guidance on the Notification of Serious Breaches, 2009).

UCL CTC will use an organisation's history of non-compliance to make decisions on future collaborations.

12.0 Trial Monitoring and oversight

Participating sites and Principal Investigators must agree to allow trial-related on-site monitoring, including Sponsor audits and regulatory inspections by providing direct access to source data/documents as required. Patients are informed of this in the patient information sheet and are asked to consent to their medical notes being reviewed by UCL CTC on the consent form.

12.1 On-site monitoring

The degree of on-site monitoring will be proportionate to the objective, purpose, phase, design, size, complexity, blinding, endpoints and risks associated with the trial. UCL CTC will determine the appropriate level and nature of monitoring required for the trial. Risk will be assessed on an ongoing basis and adjustments made accordingly.

Sites will be sent a letter in advance confirming that a routine monitoring visit is due, and including a list of the documents that are to be reviewed, interviews that will be conducted, planned inspections of the facilities, who will be performing the visit and when the visit will likely occur.

On-site monitoring will be conducted by the UCL CTC Trial Monitor/Trial Coordinator. The frequency and level of monitoring will be specified in the Trial Monitoring Plan, which will be kept in the Trial Master File and regularly updated in accordance with the aforementioned risk assessments. However, the following key areas will form the core of any on-site monitoring that occurs: consent; eligibility; capturing and reporting of Serious Adverse Events; capturing of trial endpoints; data queries; filing of essential documents in the Investigator Site File and/or trial-specific patient files; drug accountability.

Monitoring report

Following a monitoring visit, the Trial Monitor/Trial Coordinator will provide a report to the site, which will summarise the documents reviewed and a statement of findings, deviations, deficiencies, conclusions, actions taken and actions required. The Principal Investigator at each site will be responsible for ensuring that monitoring findings are addressed (this may be delegated to an appropriate member of staff).

12.2 Self-assessment monitoring

Participating sites may be requested to complete a self-assessment monitoring report periodically, at a frequency detailed in the Trial Monitoring Plan. This report may include, but is not limited to, Investigator Site File and Pharmacy File document version checklists, progress of local approval for amendments and SAE review.

Responses to self-assessment monitoring will be reviewed at the UCL CTC to identify areas of non-compliance/fraud and to indicate training needs. Findings may trigger an on-site monitoring visit.

12.3 Central monitoring

Sites will be requested to submit screening logs and staff delegation logs to UCL CTC on request and these will be checked for consistency and completeness.

Eligibility of all patients entered in the trial will be checked by an appropriately trained UCL CTC staff member prior to randomisation.

Data stored at UCL CTC will be checked for missing or unusual values (range checks) and checked for consistency over time. If any problems are identified data queries will be issued to the site. Sites are required to resolve any queries and update the relevant CRF as required. All changes must be initialled and dated. The amended version must be sent to UCL CTC and a copy retained at site.

Copies of the drug accountability logs will be collected at UCL CTC for all trial patients. Sites will be required to submit logs on request. A proportion of these will be monitored centrally to ensure completeness and correlation with data captured in the CRF.

Patients will be flagged using the NHS Information Centre (please see section 8.2 for further details). MRI scans will be submitted to UCL CTC for retrospective review for the trial, conducted by imaging experts from University College London Hospital, led by a member of the Trial Management Group.

12.4 Non-Compliance/'for cause' on-site monitoring

Additional on-site monitoring visits may be scheduled where there is evidence or suspicion of non-compliance at a site with important aspect(s) of the trial protocol/GCP requirements. Sites will be

sent a letter in advance outlining the reason(s) for the visit, a list of the documents that are to be reviewed, interviews that will be conducted, planned inspections of the facilities, who will be performing the visit and when the visit will likely occur.

UCL CTC will assess whether it is appropriate for the site to continue participation in the trial and whether the incident(s) constitute a serious breach. See section 11.0 (Incident Reporting and Serious Breaches) for details.

12.5 Oversight Committees

12.5.1 Trial Management Group (TMG)

The TMG will include the Chief Investigator, clinicians and experts from relevant specialities and DORIC trial staff from UCL CTC (see page 1). The TMG will be responsible for overseeing the trial. The group will meet regularly and will send updates to Principal Investigators (via newsletters or at Investigator meetings) and to the NCRI Brain Tumour Clinical Studies Group.

The TMG will agree protocol amendments on behalf of the PIs prior to submission to the REC and MHRA. All PIs will be kept informed of substantial amendments through their nominated responsible individuals.

12.5.2 Independent Trial Steering Committee (ITSC)

The role of the ITSC is to provide overall supervision of the trial and ensure that it is conducted in accordance with GCP and the Protocol. The ITSC will review the recommendations of the Independent Data Monitoring Committee and, on consideration of this information, recommend any appropriate amendments/actions for the trial as necessary. The ITSC acts on behalf of the funder and Sponsor.

12.5.3 Independent Data Monitoring Committee (IDMC)

The role of the IDMC is to provide independent advice on data and safety aspects of the trial. Meetings of the Committee will be held regularly (yearly or based on any safety considerations or lack of efficacy considerations). The IDMC is advisory to the ITSC and can recommend premature closure of the trial to the ITSC. The IDMC charter will define the specific responsibilities of the IDMC.

12.5.4 Role of UCL CTC

UCL CTC will be responsible for the day to day coordination and management of the trial and will act as custodian of the data generated in the trial (on behalf of UCL). UCL CTC is responsible for all duties relating to pharmacovigilance which are conducted in accordance with section 10.0 (Pharmacovigilance).

13.0 Withdrawal of patients

In consenting to the trial, patients are consenting to trial treatment, assessments, follow-up and data collection.

13.1 Withdrawal from Trial Treatment

A patient may be withdrawn from all trial treatment whenever continued participation is no longer in the patient's best interests, but the reasons for doing so must be recorded. Reasons for discontinuing treatment may include:

- Voluntary discontinuation by the patient who is at any time free to discontinue his/her participation in the study, without prejudice to further treatment. At the time of discontinuation, the investigator should establish if the patient is willing to be followed for disease progression.
- Safety reasons as judged by the investigator and/or UCL CTC
- Severe non-compliance to protocol as judged by the investigator and/or UCL CTC
- Incorrect enrolment (i.e the patient does not meet the required inclusion/exclusion criteria) of the patient if there are safety reasons as judged by the investigator and/or UCL CTC
- Patient lost to follow-up
- Disease progression (unless, in the investigator's opinion the patient is receiving benefit from study treatment. It is not compulsory to stop trial treatment following radiological progression only. Where progression is radiologically determined only, a second confirmatory scan taken 6 weeks later should be assessed before withdrawal, to rule out pseudoprogression).
- Pregnancy
- Toxicity grade 3 or above causing an IMP treatment delay for >14 days (or >21 days if CI has permitted a 21 day treatment holiday), or any other unacceptable toxicity
- Intercurrent illness which prevents further treatment
- Patients withdrawing consent to further trial treatment
- Any alterations in the patient's condition which justifies the discontinuation of treatment in the site investigator's opinion

13.1.1 Discontinuation of Trial Drugs

In addition, both trial drugs should be permanently discontinued in patients with the following conditions:

- Gastrointestinal perforation or wound dehiscence requiring medical intervention
- Serious haemorrhage, i.e., requiring medical intervention
- Severe hypertension (see section 7.4.1.4 for hypertension management protocol)
- Nephrotic syndrome
- Severe arterial thromboembolic event
- If any case of RPLS occurs that is confirmed by imaging (CT or MRI), trial drugs should be immediately discontinued, in addition to any other measures to alleviate symptoms and control blood pressure
- Patients should not receive trial drugs within 2 weeks of major surgery. Where possible, trial drugs should be suspended for 2 weeks to allow clearance before major surgery.
- Interstitial lung disease (ILD)
- Severe change in liver function

In these cases patients should continue to have trial assessments for the purposes of follow-up. If a patient wishes to withdraw from trial treatment, sites should explain the importance of remaining on trial follow-up, or failing this of allowing routine follow-up data to be used for trial purposes and for allowing existing collected data to be used.

13.1.2 Procedures for discontinuation from treatment

Patients who discontinue the trial treatment should always be asked about the reason(s) for their discontinuation and the presence of any adverse events. If possible, they should be seen and assessed by an investigator(s). The patient should return investigational products.

If all study treatment is stopped during the study, the Principal Investigator/Sub investigator will perform the best possible observation(s), test(s) and evaluation(s) as well as give appropriate treatment and all possible measures for the safety of the patient. In addition, they will record on the Case Report Form (CRF) the date of withdrawal, the reasons, and treatment at the time of withdrawal.

If only one study treatment is stopped during the study, the patient will continue with the trial assessments as before. Reasons for stopping the first drug must be recorded on the CRF. The discontinuation assessment is only to be carried out upon discontinuation of both trial drugs.

At withdrawal, all on-going SAEs must be followed until resolution, unless in the Investigator's opinion, the condition is unlikely to resolve due the patient's underlying disease.

All new AEs occurring for up to 30 days after the last dose of any study medication must be recorded in the CRF. Ongoing AEs thought to be caused by trial treatment will be followed up until resolution or death. All SAEs occurring up to 30 days after the last dose of any study medication must be followed until resolution.

Patients who are not receiving treatment, but are being followed up for tumour assessments should also be followed up for procedure related SAEs, these should be reported on an SAE report form (see section 10.2.2 SAEs). AE collection beyond the 30 days post treatment is not required for patients who have progressed and are being followed up for survival only.

For patients who are withdrawn from study treatment for reasons other than disease progression every effort will be made to continue with collecting the trial MRI scans every 6 weeks. Follow up clinic assessments should be carried out every 6 weeks as detailed in section 8.

If disease progression has been documented and the patient has withdrawn from study treatment, the long-term follow up information should be collected at least every 12 weeks by telephone contact with the patient, patient's family, or by contact with the patient's physician (see section 8). Please note that withdrawal from trial treatment is not compulsory for patients with disease progression, if the investigator feels that the patient is receiving benefit from trial treatment.

13.2 Withdrawal of Consent to Data Collection

If a patient explicitly states they do not wish to contribute further data to the trial their decision must be respected and recorded on the relevant CRF. In this event details should be recorded in the patient's hospital records, no further CRFs must be completed and no further data sent to UCL CTC.

13.3 Losses to follow-up

If a patient moves from the area, every effort should be made for the patient to be followed up at another participating trial site and for this new site to take over the responsibility for the patient, or for follow-up via GP. Details of participating trial sites can be obtained from the UCL CTC trial team

who must be informed of the transfer of care and follow up arrangements. The date of last contact should be documented when it is certain the patient is lost to follow up.

If a patient is lost to follow-up at a site every effort should be made to contact the patient's GP (if consented) to obtain information on the patient's status. Patients lost to follow up will be tracked by UCL CTC via the NHS Information Centre.

13.4 Withdrawal from Genetic Research

A patient may withdraw consent from this genetic research at any time, independent of any decision concerning participation in other aspects of the main study described in this protocol. Voluntary discontinuation by the patient will not prejudice further treatment.

13.4.1 Procedures for discontinuation from genetic aspects of the study

Patients who discontinue from the study should always be asked specifically whether they are withdrawing or continuing their consent for this genetic research. It must be established whether the patient:

- Agrees to the genetic sample and any DNA extracted from the sample being kept for genetic research in the future.

- Withdraws consent for the sample to be kept for genetic research in the future and wishes the sample to be destroyed. Destruction of the sample (or the DNA extracted from the sample) will only be possible so long as the particular sample is traceable. In the event that genetic research has already been performed, UCL CTC will retain the results and associated data for regulatory reasons but these will not be used in any subsequent analyses.

Depending on which samples the patient had originally consented to give, the above should be checked for the use of tissue biopsy samples, blood samples and / or post-mortem tumour samples.

The investigator is responsible for providing written notification to UCL CTC of any patient who has withdrawn consent for the use or future donation of any sample taken for genetic research, and specifying which particular samples. UCL CTC will provide written confirmation to the investigator of

the actions taken with the request (including details of sample destruction where relevant), which must be filed in the investigator study file.

14.0 Trial Closure

14.1 End of Trial

For regulatory purposes the end of the trial will be 6 months from the date the last patient finished trial treatment (the day after the date that the last trial drug was taken, i.e the first day that the patient is no longer taking either trial drug (not including treatment holidays of <14 days, or 21 days in consultation with the CI. If a patient has a treatment holiday which then is extended to withdrawal from the trial, the date of finishing trial treatment will be the first day of the treatment holiday)), or the date the last patient dies, whichever is sooner, at which point the 'declaration of end of trial' form will be submitted to participating regulatory authorities and ethical committees, as required.

If patients are alive following the end of trial, they will be followed up every 12 weeks for survival as per section 8.1 (Schedule of assessments).

14.2 Archiving of Trial Documentation

At the end of the trial, UCL CTC will archive securely all centrally held trial related documentation for a minimum of 5 years. Arrangements for confidential destruction will then be made. It is the responsibility of Principal Investigators to ensure data and all essential documents relating to the trial held at site are retained for a minimum of 5 years after the end of the trial, in accordance with national legislation and for the maximum period of time permitted by the site.

Essential documents are those which enable both the conduct of the trial and the quality of the data produced to be evaluated and show whether the site complied with the principles of Good Clinical Practice and all applicable regulatory requirements.

UCL CTC will notify sites when trial documentation held at sites may be archived. All archived documents must continue to be available for inspection by appropriate authorities upon request.

14.3 Early discontinuation of trial

The trial may be stopped before completion as an Urgent Safety Measure on the recommendation of the ITSC or IDMC (see section 12.5.2 ITSC and section 12.5.3 IDMC). Sites will be informed in

writing by UCL CTC of reasons for early closure and the actions to be taken with regards the treatment and follow up of patients.

14.4 Withdrawal from trial participation by a site

Should a site choose to close to recruitment the PI must inform UCL CTC in writing. Follow up as per protocol must continue for all patients recruited into the trial at that site and other responsibilities continue as per CTSA.

15.0 Quality Assurance

All MRI scans used to produce the outcome data for the final analysis will undergo a retrospective central review at the end of the trial as per section 7.7, before analysis. Local treatment decisions will be based on local imaging results.

16.0 Statistics

16.1 Sample size calculation

Statistical methods and determination of sample size

In the UK there is no standard second line treatment. Lomustine based treatments may be given but have a median PFS of only 2 months (8.32 weeks) (Yung et al. 2000). However, on the basis of the FDA approval for bevacizumab in GBM we suggest that the AVF3708g and NCI 06-C-0064E studies serve as the comparator for outcomes for this phase II study:

AVF3708g and NCI 06-C-0064E studies

The AVF3708g study was a phase II trial in 167 patients (85 treated with single-agent bevacizumab) with glioblastoma at first or second relapse (Friedman, 2010). Of note only the efficacy data from the bevacizumab monotherapy arm (n=85) was used to support FDA drug approval. Findings were supported by the Phase II trial of bevacizumab in 48 patients with relapsed glioblastoma (Study NCI 06-C-0064E) (Kriesl, 2009).

- AVF3708g study - objective response rate was 28.2 % (95% CI: 18.5 -40.3%)
- AVF3708g study - median response duration was 5.6 months (95% CI: 3.0-5.8 months)
- AVF3708g study - the 6-month progression-free survival (PFS6) rate was 42.6% (97.5% CI: 29.6-55.5%)
- AVF3708g study - the 6-month PFS rates for patients at first and second relapse were 46.4% and 27.8% respectively
- AVF3708g study – the median PFS in first and second relapse was 4.4 and 3.1 months respectively
- AVF3708g study - the median overall survival was 9.2 months (95% CI: 8.2-10.7 months), median overall survival at first and second relapse was 9.1 months and 9.2 months respectively
- Study NCI 06-C-0064E - response rate was 35% percent by Macdonald criteria (one CR, 16 PRs)
- Study NCI 06-C-0064E – median PFS of 16 weeks (95% CI: 12-26 weeks)

Assuming a median PFS of 4 months with cediranib (as equivalent to bevacizumab) and an expected median PFS of 6 months on the combination cedirinib plus gefitinib arm a total of 112 patients are required to achieve 110 events in order to detect a hazard ratio of about 0.67 between the two arms. This assumes an accrual period of 18 months, maximum follow up of 36 months, lambda 1= 0.115, lambda 2=0.1733,and a one sided type I error of 10% with approximate power of 80%. The sample size was generated using Nquery Version 7.0.

16.2 Population for analysis

The primary population will be the Intention to Treat (ITT) population defined as all patients randomized irrespective of having been administered at least one dose of randomized study medication . All primary and secondary efficacy endpoints will be analysed using this population.

The safety population will be all patients randomized and having taken at least one dose of randomized treatment. The primary endpoint will also be analysed using this population.

16.3 Statistical Analysis of the primary endpoint

- **Progression free survival (PFS) defined as the time from the date of randomisation to the date of first progression or death due to any cause (see section 2.6.1.1)**

This endpoint will be analysed using a Cox proportional hazards model. All stratified covariates will be included in the model as well as additional covariates which are deemed to influence the response. These will be stated *a priori* in the statistical analysis plan (SAP). The 90% confidence interval for the Hazard ratio will be derived which will provide an estimate of the plausible ranges of the treatment effect. A Kaplan Meier plot of Survival probabilities over time will also be generated. Patients who take any concomitant medications which are likely to influence the primary endpoint will be censored at the date of starting the medication (if available). Patients who have not progressed or died will be censored at the last date of contact.

The primary endpoint will be analysed using both the ITT and Safety Populations. Additional analysis will be conducted as is deemed appropriate and documented in the SAP. An exploratory analysis will also be carried out using the unmodified RANO criteria (Wen et al 2010).

16.4 Statistical Analysis of the Secondary Endpoints

The secondary endpoints will be subject to an ITT analysis only.

- **Overall survival (OS)**

This is defined as time from date of randomization to date of Death due to any cause. This endpoint will be analysed in a similar way to the primary endpoint

- **Radiographic response rate (RR) at each assessment point**

The response rates will be summarised by treatment groups in terms of counts and percentages. The following definition of responses will be used.

- **Complete response:** Requires all of the following: disappearance of all contrast enhancing lesions on MRI compared to baseline sustained for 2 consecutive 6 weekly scans*; no new lesions; stable or improved nonenhancing (T2/FLAIR) lesions; patients must be off dexamethasone (or on physiologic replacement doses only); and stable or improved clinically. Patients with non-measureable disease only cannot have a complete response; the best response possible is stable disease.
- **Partial response**: Requires all of the following: ≥ 50% decrease compared with baseline in the sum of products of perpendicular diameters of all measureable enhancing disease percentage change sustained for 2 consecutive 6 weekly scans*; no progression of non measurable disease; no new lesions; stable or improved nonenhancing (T2/FLAIR) lesions on same or lower dose of dexamethasone compared with the baseline scan; the dexamethasone dose at the time of the scan evaluation should be no greater than the dose at the time of baseline scan; and stable or improved clinically. Note: Patients with non-measureable disease only cannot have a complete response; the best response possible is stable disease.
- **Progression**: (See Section 2.6.1.1)

  Stable Disease: Requires all of the following: a patient whose MRI scan does not meet the criteria for *Complete Response, Partial Response, or Progressive Disease*: stable nonenhancing (T2/FLAIR) lesions on same or lower dose of dexamethasone compared with baseline scan. In the event that the dexamethasone dose was increased for new symptoms and signs without confirmation of disease progression on neuroimaging, and subsequent follow-up imaging shows that this increase in dexamethasone dose was required because of disease progression, the last scan considered to show stable disease will be the scan obtained with the dexamethasone dose was equivalent to the baseline dose.

*In the absence of a confirmatory scan at 6 weeks later for CR or PR these should be recorded at a visit level as unconfirmed CR or PR, but the best overall response would be stable disease

For further details see the RANO criteria in Wen et al (2010).

- **PFS rate at 6 months (24 weeks)**

Progression is defined in the same way as described for the primary endpoint. This endpoint will be analysed in the same way as the primary endpoint. A comparison of the PFS rate at 6 month/ 24 weeks will be made and the 95% confidence interval will be generated for the difference in the PFS rate adjusted for any covariates. A survival plot will also be generated.

- **Overall Survival rate at 12 months.**

This will be analysed in the same way as the PFS and OS endpoints (above)

- **Time to sustained increase in total daily steroid dose by ≥ 8mg/day compared to baseline or sustained nadir** (≥ 21 days)

This is defined as time from randomization to first increase in dexamethasone dose. An increase in dexamethasone dose is defined as a change from the baseline of ≥8mg/day for at least 21 days (i.e. the increase is sustained for at least 21 days consecutively). The earliest date when the dexamethasone dose was ≥ 8mg/day compared to baseline or sustained nadir in this period will be used for comparison between the treatment groups. Sustained nadir refers to the highest steroid dose taken during a ≥ 21 days period where the patient is consistently taking a lower dose of steroids compared to the baseline dose. Daily dexamethasone dose will be recorded until progression.

This endpoint will be analysed as a time to event endpoint similarly to the OS and PFS. Additional covariates may also be included in the analyses for adjustment.

- **Average daily steroid dosage change from baseline until first progression**
- **Average number of progression/steroid free days**

For the above 2 endpoints the mean steroid dosage prior to treatment will be considered as the patient's baseline. The change in average daily steroid dosage from baseline is calculated as the mean daily dexamethasone dosage recorded from the first day of therapy to progression subtracted from baseline. If a patient does not progress, average daily steroid dosage is calculated as the mean

of daily actual dexamethasone dosage recorded prior to the last date the patient is recorded to have had a dose of dexamethasone.

The number of progression/steroid free days is the number of days that a patient is known not to have used any steroids (dexamethasone) prior to progression. This will be summarized by treatment group.

- **Time to deterioration of neurological status or death**

This endpoint will be summarized by treatment group. Neurological status based on investigator's neurological examination prior to and during treatment will be recorded on the CRF.

Patient's last pre-dose assessment will be defined as baseline. Patients without a baseline assessment will be excluded from analysis. Time to neurological status deterioration will be defined as the date of randomization to the date of first neurological status worsening in comparison to baseline (first of 2 confirmatory reports at 2 consecutive visits, 6 weeks apart) as assessed by the clinician, or until date of death, whichever is first.

Quality of Life

- **EORTC QLQ-C30**

Each domain will be summarised. The change from baseline will be summarized. For specific domains, a model based analysis will be undertaken (such as Mixed effects models). The scoring of the items/scales will be as described in the EORTC QOL Scoring Manual. Patients without a baseline assessment will be excluded from the analysis.

- **EORTC brain tumour module BN-20 questionnaire - Neurological symptoms (visual disorder, motor dysfunction, communication deficit, and drowsiness)**

Patients with recurrent glioblastoma commonly experience neurological symptoms. Therefore, improvement in visual disorders, motor dysfunction, communication deficits and drowsiness between the treatment arms for patients with neurological symptoms relative to baseline will be summarized by treatment group. Improvement in symptoms should also be concurrent with stable or decreasing use of steroids (dexamethasone). Visual disturbances will be assessed using questions 36, 37 and 38 of the BN-20. Motor dysfunction will be assessed using questions 40, 45, and 49 of the BN-20.

Communication deficits will be assessed using question 41, 42 and 43 of the BN-20. Drowsiness will be assessed by question 44.

16.5 Statistical Analysis of Safety Data

The following safety endpoints below will all be summarized using appropriate summary statistics. Changes from baseline may be conducted as is deemed appropriate. Safety data will be analysed and reported on an ongoing basis for IDMC meetings (where data is available).

- Adverse Events (AE)
- Vital signs including blood pressure (BP)
- Electrocardiogram (ECG) parameters
- Laboratory findings (clinical chemistry, haematology, urinalysis, TSH, T3 and T4)
- Physical examination including neurological examination

Safety data will be summarized as deemed appropriate. Relevant changes from baseline may be reported and summarized by treatment group. SAEs will be summarized by grade and treatment group. Safety data will be analysed and reported on an ongoing basis for IDMC meetings (where data is available).

Stopping criteria

A treatment-related grade ≥3 toxicity rate of 30% in any treatment group along with any other clinical data agreed by the Independent data Monitoring Committee would be used as a guide to consider stopping the trial. As soon as 17 patients out of 56 (30%) in one of either arm suffer a grade ≥3 treatment-related event, this would prompt us to review the safety data more frequently. If at least 24 patients (43%) have a grade ≥3 treatment-related event, this would require the IDMC to consider stopping the trial early for safety reasons. The IDMC will be able to compare the toxicity rates between the treatment arms. All safety data will be reviewed regularly. This will be at least every 6 months by members of the TMG in a blinded fashion and at every IDMC meeting. Should stopping be considered due to safety resons, the IDMC will meet at the earliest convenient time.

16.6 Statistical Analysis of other endpoints

Pharmacodynamic and Pharmacogenetic endpoints

- Levels of soluble markers of angiogenesis and tumour growth including changes from baseline using optional blood samples
- Biomarker analysis from archival and postmortem tumour samples
- Pharmacogenetic analysis using optional blood samples and/or tumour samples

Blood samples (plasma and serum) will be collected and assessed for biomarkers of disease activity, effect of cediranib and gefitinib, and angiogenesis. Archival tumour samples (from the primary tumour and/or any recurrent disease) will be collected from patients who provide informed consent for the retrospective analysis of biomarkers. Biomarkers will be investigated for possible correlation with key clinical outcomes and for the effects of the study medication.

Appropriate summaries of absolute levels and changes from baseline in soluble biomarkers will be produced, as well as correlates with clinical efficacy. Summaries of biomarkers in archival tumour samples and appropriate correlates will be produced. Additional analysis may be conducted as deemed appropriate.

16.7 Handling missing data

Missing data will be left as missing and no imputation will be carried out. Further details where relevant will be outlined in the SAP.

16.8 Interim Analysis

No formal interim analysis is planned. However data is reviewed on an ongoing basis for the IDMC. In addition, conditional power estimates may be provided to determine futility of the trial based on the observed and assumed hazard ratio. This will be calculated at 50% of the required events and 75% of the events stated in the sample size section. In general a conditional power of < 20% is suggestive of futility, however clinical judgment will also be taken into account before concluding formal futility of the trial.

17.0 Ethical and Regulatory Approvals

In conducting the Trial the Sponsor, UCL CTC and Sites shall also comply with all laws and statutes, as amended from time to time, applicable to the performance of clinical trials including, but not limited to:

- the principles of ICH Harmonised Tripartite Guideline for Good Clinical Practice (CPMP/ICH/135/95) as set out in Schedule 1 (Conditions and Principles of Good Clinical Practice and for the Protection of Clinical Trial Subjects) of the Medicines for Human Use (Clinical Trials) Regulations 2004 and the GCP Directive 2005/28/EC, as set out in SI 2006/1928
- the Human Rights Act 1998
- the Data Protection Act 1998
- the Freedom of Information Act 2000
- the Human Tissue Act 2004
- the Medicines Act 1968
- the Medicines for Human Use (Clinical Trials) UK Regulations SI 2004/1031, and subsequent amendments
- Good Manufacturing Practice
- the Research Governance Framework for Health and Social Care, issued by the UK Department of Health (Second Edition 2005) or the Scottish Health Department Research Governance Framework for Health and Community Care (Second Edition 2006)

17.1 Ethical Approval

The trial will be conducted in accordance with the World Medical Association Declaration of Helsinki entitled 'Ethical Principles for Medical Research Involving Human Subjects' (1996 version) and in accordance with the terms and conditions of the ethical approval given to the trial.

The trial has received a favourable opinion from the [REC name to be inserted when favourable opinion granted] Research Ethics Committee.

UCL CTC will submit Annual Progress Reports to the REC, which will commence one year from the date of ethical approval for the trial.

17.2 Regulatory Approval

A Clinical Trial Authorisation (CTA) has been granted for the trial.
The trial will be conducted at approved trial sites in accordance with the trial protocol and the terms of the CTA granted by the MHRA.

17.3 Local Site Approval

The Lead Comprehensive Local Research Network (CLRN) has given NHS permission following global governance checks. Local checks will be undertaken by local CLRNs associated with individual trial sites.

Evidence of local Trust R&D approval must be provided to UCL CTC prior to site activation. The trial will only be conducted at sites where all necessary approvals for the trial have been obtained.

17.4 Protocol Amendments

UCL CTC will be responsible for gaining ethical and regulatory approvals for all amendments made to the protocol and other trial-related documents. Once approved, UCL CTC will ensure that all amended documents are distributed to sites and CLRNs as appropriate.

Site staff will be responsible for acknowledging receipt of documents and for gaining local Trust R&D acknowledgement for all amendments and approval for substantial amendments, and for providing UCL CTC with evidence of this.

17.5 Patient Confidentiality & Data Protection

Patient identifiable data, including initials, date of birth, and NHS number will be required for the randomisation process and will be provided to UCL CTC. UCL CTC will preserve patient confidentiality and will not disclose or reproduce any information by which patients could be identified. Data will be stored in a secure manner and UCL CTC trials are registered in accordance with the Data Protection Act 1998 with the Data Protection Officer at UCL.

18.0 Sponsorship and Indemnity

18.1 Sponsor Details:

Name: University College London
Address: Gower Street
London
WC1E 6BT

Sponsor contact:
Senior Clinical Trials Manager or Divisional Manager

Tel: 020 7380 9995/6978 (unit admin)
Fax: 020 7380 9937
Postal Address: Joint UCLH and UCL Biomedical Research Unit
(1st floor, Maple House)
Ground Floor, Rosenheim Wing
25 Grafton Way
London
WC1E 5DB

18.2 Indemnity

University College London holds insurance to cover participants for injury caused by their participation in the clinical trial. Participants may be able to claim compensation if they can prove that UCL has been negligent. However, as this trial is being carried out in a hospital, the hospital continues to have a duty of care to the participant of the trial. University College London does not accept liability for any breach in the hospital's duty of care, or any negligence on the part of hospital employees. This applies whether the hospital is an NHS Trust or not. This does not affect the participant's right to seek compensation via the non-negligence route.

Participants may also be able to claim compensation for injury caused by participation in this trial without the need to prove negligence on the part of University College London or another party. Participants who sustain injury and wish to make a claim for compensation should do so in writing in the first instance to the Chief Investigator, who will pass the claim to the Sponsor's Insurers, via the Sponsor's office.

Hospitals selected to participate in this trial shall provide clinical negligence insurance cover for harm caused by their employees and a copy of the relevant insurance policy or summary shall be provided to University College London, upon request.

19.0 Funding

Cancer Research UK and AstraZeneca are supporting the central coordination of the trial through the UCL CTC. AstraZeneca will be providing the cediranib, gefitinib and placebo free of charge.

20.0 Publication Policy

All publications and presentations relating to the trial will be authorised by the Trial Management Group (TMG). The first publication of the trial results will be written by and in the name of the TMG, relevant trial staff from UCL CTC, and the Trial statistician. Authors will be cited by name if published in a journal where this does not conflict with the journal's policy. Contributing site Investigators (PIs plus Co-Investigators and research nurses where appropriate) will be acknowledged, along with AstraZeneca and the trial participants (anonymously). Data from all sites will be analysed together and published as soon as possible. Participating sites may not publish trial results prior to the first publication by the TMG or without prior written consent from the TMG. The trial data are owned by UCL the sponsor. However, AstraZeneca will be permitted to see the draft manuscripts and make comments at least 30 days prior to submission for publication. The Clinicaltrials.gov number allocated to this trial will be quoted in any publications based upon its results.

21.0 Translational Research

An exploratory translational part of the research will look at blood, tumour and post-mortem samples for which additional and optional consent will be obtained from patients.

The objectives of the translational research are:

- To investigate the relationship between the effects of cediranib on soluble angiogenesis biomarkers and clinical efficacy

- To obtain blood samples for pharmacogenetics (genetic variation giving rise to different response to the IMPs)

- To obtain archival tumour biopsy samples for biomarker analysis and potential sub-group stratification for:
    - IDH1 and 2 (Yan et al. 2009)
    - MGMT methylation status
    - EGFR, EGFRvIII and PTEN (Mellinghoff et al. 2005)

- To obtain post-mortem tumour samples for research into genomic sequence and/or gene expression patterns associated with response or resistance to cediranib and/or gefitinib.

As consent for this part of the research is optional, it is not known whether sufficient data will be generated to explore all of the above objectives. Patients will consent to donate samples for future research, consent will be generic and enduring and include consent for genetic research. Consent for blood and tumour samples will be separate from post-mortem samples.

Blood samples will be taken at time-points outlined in the schedule of assessments (section 8.1). Staff members taking samples should be appropriately qualified to do so. These will include serum samples and whole blood samples in EDTA. Samples will be stored locally at sites in freezers and collected in batches (remaining frozen) for storage at the laboratory in UCL.

Archival tumour biopsies will be collected from patients where they have given consent for this. Post mortem tumour samples will be collected where patients have given additional consent for this. These samples will be collected and stored at the laboratory in UCL.

Sample collection, storage and shipping arrangements will be described in a separate Laboratory Manual. Sample labels will refer to patient trial number, but not to patient names.

Translational research will be retrospective to the trial. Translational research will be in line with the current evolving literature at the end of the trial and available resources and where possible will also include the following: EGFR mutation analysis, amplification of 4q (VEGFR2, PDGFR, c-Kit), and VEGF/VEGFR polymorphisms. Where possible, DNA and/or RNA will be extracted from blood and tumour samples and analysed using methods such as whole-genome sequencing, microarray analysis and gene methylation profiling. Any additional objectives for the translational research will be submitted for additional ethics approval.

Extraction of post-mortem samples will be carried out by local pathologists and will only be available at sites where this has been agreed in the clinical trial site agreement. Families of patients who have consented to donation of post-mortem samples will need to contact the local investigator at the site to arrange this. Post-mortem tissue samples taken will be of the tumour and brain only.

SAMPLE	Baseline	Week 6	Week 12	Week 18	Week 24	Discontinuation	Death
Pharmacodynamic (serum)	10ml (drawn)	10ml (drawn)	10ml (drawn)	10ml (drawn)	10ml (drawn)	10ml (drawn)	
Pharmacodynamic (whole blood)	10-20ml in EDTA	10-20ml in EDTA	10-20ml in EDTA	10-20ml in EDTA	10-20ml in EDTA	10-20ml in EDTA	
Pharmacogenetics (whole blood)	10-20ml in EDTA						
Archival tumour sample	Collected at end or during trial						
Post-mortem tumour sample							Collected after contact from family

Table 12 Samples proposed for collection for translational research

If there might be any problems with sample collection at the site, the DORIC trial coordinator should be contacted prior to site activation.

22.0 References

Akagi, M., M. Kawaguchi, W. Liu, M.F. McCarty, A. Takeda, F. Fan, O. Stoeltzing, A.A. Parikh, Y.D. Jung, C.D. Bucana, P.F. Mansfield, D.J. Hicklin, and L.M. Ellis. 2003. Induction of neuropilin-1 and vascular endothelial growth factor by epidermal growth factor in human gastric cancer cells. Br J Cancer 88: 796-802.

Batchelor, T.T., A.G. Sorensen, E. di Tomaso, W.T. Zhang, D.G. Duda, K.S. Cohen, K.R. Kozak, D.P. Cahill, P.J. Chen, M. Zhu, M. Ancukiewicz, M.M. Mrugala, S. Plotkin, J. Drappatz, D.N. Louis, P. Ivy, D.T. Scadden, T. Benner, J.S. Loeffler, P.Y. Wen, and R.K. Jain. 2007. AZD2171, a pan-VEGF receptor tyrosine kinase inhibitor, normalizes tumor vasculature and alleviates edema in glioblastoma patients. Cancer Cell 11: 83-95.

Batchelor, T., P. Mulholland, B. Neyns, L.B. Nabors, M. Campone, A. Wick, W. Mason, J. Xu, Q. Liu, M. van den Bent. 2010. Abstract LBA7 A phase III randomised study comparing the efficacy of cediranib as monotherapy, and in combination with lomustine, with lomustine alone in recurrent glioblastoma patients. Presented at the European Society for Medical Oncology 35th Annual Congress, Milan October 8-12 2010.

Benson, A.B., 3rd, J.A. Ajani, R.B. Catalano, C. Engelking, S.M. Kornblau, J.A. Martenson, Jr., R. McCallum, E.P. Mitchell, T.M. O'Dorisio, E.E. Vokes, and S. Wadler. 2004. Recommended guidelines for the treatment of cancer treatment-induced diarrhea. J Clin Oncol 22: 2918-2926.

Bianco, R., S. Garofalo, R. Rosa, V. Damiano, T. Gelardi, G. Daniele, R. Marciano, F. Ciardiello, and G. Tortora. 2008. Inhibition of mTOR pathway by everolimus cooperates with EGFR inhibitors in human tumours sensitive and resistant to anti-EGFR drugs. Br J Cancer 98: 923-930.

Bukowski, R.M., F.F. Kabbinavar, R.A. Figlin, K. Flaherty, S. Srinivas, U. Vaishampayan, H.A. Drabkin, J. Dutcher, S. Ryba, Q. Xia, F.A. Scappaticci, and D. McDermott. 2007. Randomized phase II study of erlotinib combined with bevacizumab compared with bevacizumab alone in metastatic renal cell cancer. J Clin Oncol 25: 4536-4541.

Camp, E.R., J. Summy, T.W. Bauer, W. Liu, G.E. Gallick, and L.M. Ellis. 2005. Molecular mechanisms of resistance to therapies targeting the epidermal growth factor receptor. Clin Cancer Res 11: 397-405.

Camus, P., S. Kudoh, and M. Ebina. 2004. Interstitial lung disease associated with drug therapy. Br J Cancer 91 Suppl 2: S18-23.

Carro, M.S., W.K. Lim, M.J. Alvarez, R.J. Bollo, X. Zhao, E.Y. Snyder, E.P. Sulman, S.L. Anne, F. Doetsch, H. Colman, A. Lasorella, K. Aldape, A. Califano, and A. Iavarone. 2009. The transcriptional network for mesenchymal transformation of brain tumours. Nature.

Chen, Z., X. Zhang, M. Li, Z. Wang, H.S. Wieand, J.R. Grandis, and D.M. Shin. 2004. Simultaneously targeting epidermal growth factor receptor tyrosine kinase and cyclooxygenase-2, an efficient approach to inhibition of squamous cell carcinoma of the head and neck. Clin Cancer Res 10: 5930-5939.

Ciardiello, F., R. Bianco, R. Caputo, R. Caputo, V. Damiano, T. Troiani, D. Melisi, F. De Vita, S. De Placido, A.R. Bianco, and G. Tortora. 2004. Antitumor activity of ZD6474, a vascular endothelial growth factor receptor tyrosine kinase inhibitor, in human cancer cells with acquired resistance to antiepidermal growth factor receptor therapy. Clin Cancer Res 10: 784-793.

Ciardiello, F., R. Caputo, R. Bianco, V. Damiano, G. Fontanini, S. Cuccato, S. De Placido, A.R. Bianco, and G. Tortora. 2001. Inhibition of growth factor production and angiogenesis in human cancer cells by ZD1839 (Iressa), a selective epidermal growth factor receptor tyrosine kinase inhibitor. Clin Cancer Res 7: 1459-1465.

Ciardiello, F., T. Troiani, R. Bianco, M. Orditura, F. Morgillo, E. Martinelli, M.P. Morelli, T. Cascone, and G. Tortora. 2006. Interaction between the epidermal growth factor receptor (EGFR) and the vascular endothelial growth factor (VEGF) pathways: a rational approach for multi-target anticancer therapy. Ann Oncol 17 Suppl 7: vii109-114.

Claes, A., P. Wesseling, J. Jeuken, C. Maass, A. Heerschap, and W.P. Leenders. 2008. Antiangiogenic compounds interfere with chemotherapy of brain tumors due to vessel normalization. Mol Cancer Ther 7: 71-78.

Clarke, K., K. Smith, W.J. Gullick, and A.L. Harris. 2001. Mutant epidermal growth factor receptor enhances induction of vascular endothelial growth factor by hypoxia and insulin-like growth factor-1 via a PI3 kinase dependent pathway. Br J Cancer 84: 1322-1329.

Cohen, E.E., D.W. Davis, T.G. Karrison, T.Y. Seiwert, S.J. Wong, S. Nattam, M.F. Kozloff, J.I. Clark, D.H. Yan, W. Liu, C. Pierce, J.E. Dancey, K. Stenson, E. Blair, A. Dekker, and E.E. Vokes. 2009a. Erlotinib and bevacizumab in patients with recurrent or metastatic squamous-cell carcinoma of the head and neck: a phase I/II study. Lancet Oncol 10: 247-257.

Cohen, M.H., Y.L. Shen, P. Keegan, and R. Pazdur. 2009b. FDA drug approval summary: bevacizumab (Avastin) as treatment of recurrent glioblastoma multiforme. Oncologist 14: 1131-1138.

Cohen, M.H., G.A. Williams, R. Sridhara, G. Chen, and R. Pazdur. 2003. FDA drug approval summary: gefitinib (ZD1839) (Iressa) tablets. Oncologist 8: 303-306.

Dickler, M.N., H.S. Rugo, C.A. Eberle, E. Brogi, J.F. Caravelli, K.S. Panageas, J. Boyd, B. Yeh, D.E. Lake, C.T. Dang, T.A. Gilewski, J.F. Bromberg, A.D. Seidman, G.M. D'Andrea, M.M. Moasser, M. Melisko, J.W. Park, J. Dancey, L. Norton, and C.A. Hudis. 2008. A phase II trial of erlotinib in combination with bevacizumab in patients with metastatic breast cancer. Clin Cancer Res 14: 7878-7883.

Dinnes J, Cave C, Huang S et al. 2000. The effectiveness and cost-effectiveness of temozolomide for the treatment of recurrent malignant glioma. Draft report to NICE, Wessex Institute for Health Research and Development.

Ekstrand, A.J., C.D. James, W.K. Cavenee, B. Seliger, R.F. Pettersson, and V.P. Collins. 1991. Genes for epidermal growth factor receptor, transforming growth factor alpha, and epidermal growth factor and their expression in human gliomas in vivo. Cancer Res 51: 2164-2172.

Ellis, L.M. 2004. Epidermal growth factor receptor in tumor angiogenesis. Hematol Oncol Clin North Am 18: 1007-1021, viii.

Esteva, F.J. 2004. Monoclonal antibodies, small molecules, and vaccines in the treatment of breast cancer. Oncologist 9 Suppl 3: 4-9.

Flanigan, J, H. Deshpande, S. Gettinger. 2010. Current status of vandetanib (ZD6474) in the treatment of non-small cell lung cancer. Biologics 4. 237-43.

Franceschi, E., G. Cavallo, S. Lonardi, E. Magrini, A. Tosoni, D. Grosso, L. Scopece, V. Blatt, B. Urbini, A. Pession, G. Tallini, L. Crino, and A.A. Brandes. 2007. Gefitinib in patients with progressive high-grade gliomas: a multicentre phase II study by Gruppo Italiano Cooperativo di Neuro-Oncologia (GICNO). Br J Cancer 96: 1047-1051.

Frederick, L., X.Y. Wang, G. Eley, and C.D. James. 2000. Diversity and frequency of epidermal growth factor receptor mutations in human glioblastomas. Cancer Res 60: 1383-1387.

Friedman, H.S., M.D. Prados, P.Y. Wen, T. Mikkelsen, D. Schiff, L.E. Abrey, W.K. Yung, N. Paleologos, M.K. Nicholas, R. Jensen, J. Vredenburgh, J. Huang, M. Zheng, and T. Cloughesy. 2009. Bevacizumab alone and in combination with irinotecan in recurrent glioblastoma. J Clin Oncol 27: 4733-4740.

Grossman, S.A., X. Ye, S. Piantadosi, S. Desideri, L.B. Nabors, M. Rosenfeld, and J. Fisher. 2010. Survival of patients with newly diagnosed glioblastoma treated with radiation and temozolomide in research studies in the United States. Clin Cancer Res 16: 2443-2449.

Hainsworth, J.D., J.A. Sosman, D.R. Spigel, D.L. Edwards, C. Baughman, and A. Greco. 2005. Treatment of metastatic renal cell carcinoma with a combination of bevacizumab and erlotinib. J Clin Oncol 23: 7889-7896.

Hainsworth, J.D., D.R. Spigel, C. Farley, D.S. Thompson, D.L. Shipley, and F.A. Greco. 2007. Phase II trial of bevacizumab and erlotinib in carcinomas of unknown primary site: the Minnie Pearl Cancer Research Network. J Clin Oncol 25: 1747-1752.

Hasselbalch, B., U. Lassen, S. Hansen, M. Holmberg, M. Sorensen, M. Kosteljanetz, H. Broholm, M.T. Stockhausen, and H.S. Poulsen. 2010. Cetuximab, bevacizumab, and irinotecan for patients with primary glioblastoma and progression after radiation therapy and temozolomide: a phase II trial. Neuro Oncol 12: 508-516.

Hegi, M.E., A.C. Diserens, T. Gorlia, M.F. Hamou, N. de Tribolet, M. Weller, J.M. Kros, J.A. Hainfellner, W. Mason, L. Mariani, J.E. Bromberg, P. Hau, R.O. Mirimanoff, J.G. Cairncross, R.C. Janzer, and R. Stupp. 2005. MGMT gene silencing and benefit from temozolomide in glioblastoma. N Engl J Med 352: 997-1003.

Herbst, R.S., D.H. Johnson, E. Mininberg, D.P. Carbone, T. Henderson, E.S. Kim, G. Blumenschein, Jr., J.J. Lee, D.D. Liu, M.T. Truong, W.K. Hong, H. Tran, A. Tsao, D. Xie, D.A. Ramies, R. Mass, S. Seshagiri, D.A. Eberhard, S.K. Kelley, and A. Sandler. 2005. Phase I/II trial evaluating the anti-vascular endothelial growth factor monoclonal antibody bevacizumab in combination with the HER-1/epidermal growth factor receptor tyrosine kinase inhibitor erlotinib for patients with recurrent non-small-cell lung cancer. J Clin Oncol 23: 2544-2555.

Herbst, R.S., P.M. LoRusso, M. Purdom, and D. Ward. 2003. Dermatologic side effects associated with gefitinib therapy: clinical experience and management. Clin Lung Cancer 4: 366-369.

Herbst R.S., V.J. O'Neill, L. Fehrenbacher, C.P. Belani, P.D.Bonomi., L. Hart., O. Melnyk, D.Ramies, M.Lin, and A.Sandler. 2007. Phase II study of efficacy and safety of bevacizumab in combination with chemotherapy or erlotinib compared with chemotherapy alone for treatment of recurrent or refractory non small-cell lung cancer. J Clin Oncol 25:4743–50.

Herndon J, Vredenburgh J, Reardon D, Desjardins A, Peters K, Gururangan S, Norfleet J, Friedman A, Bigner D, Friedman HS. 2009. Phase I trial of vendetanib and oral etoposide for recurrent malignant gliomas. J Clin Oncol 27 (suppl; abstr e13016)

Hinchey, J., C. Chaves, B. Appignani, J. Breen, L. Pao, A. Wang, M.S. Pessin, C. Lamy, J.L. Mas, and L.R. Caplan. 1996. A reversible posterior leukoencephalopathy syndrome. N Engl J Med 334: 494-500.

Holtkamp, N., N. Ziegenhagen, E. Malzer, C. Hartmann, A. Giese, and A. von Deimling. 2007. Characterization of the amplicon on chromosomal segment 4q12 in glioblastoma multiforme. Neuro Oncol 9: 291-297.

Jain, R.K., E. di Tomaso, D.G. Duda, J.S. Loeffler, A.G. Sorensen, and T.T. Batchelor. 2007. Angiogenesis in brain tumours. Nat Rev Neurosci 8: 610-622.

Kreisl, T.N., L. Kim, K. Moore, P.Duic, C. Royce, I. Stroud, N. Garren, M. Mackey, J.A. Butman, K. Camphausen, J. Park, P.S. Albert, H.A. Fine. 2009. Phase II trial of single-agent bevacizumab followed by bevacizumab plus irinotecan at tumor progression in recurrent glioblastoma. J Clin Oncol. 27(5). 740-5.

Libermann, T.A., H.R. Nusbaum, N. Razon, R. Kris, I. Lax, H. Soreq, N. Whittle, M.D. Waterfield, A. Ullrich, and J. Schlessinger. 1985. Amplification, enhanced expression and possible rearrangement of EGF receptor gene in primary human brain tumours of glial origin. Nature 313: 144-147.

Maity, A., N. Pore, J. Lee, D. Solomon, and D.M. O'Rourke. 2000. Epidermal growth factor receptor transcriptionally up-regulates vascular endothelial growth factor expression in human glioblastoma cells via a pathway involving phosphatidylinositol 3'-kinase and distinct from that induced by hypoxia. Cancer Res 60: 5879-5886.

Mellinghoff, I.K., M.Y. Wang, I. Vivanco, D.A. Haas-Kogan, S. Zhu, E.Q. Dia, K.V. Lu, K. Yoshimoto, J.H. Huang, D.J. Chute, B.L. Riggs, S. Horvath, L.M. Liau, W.K. Cavenee, P.N. Rao, R. Beroukhim, T.C. Peck, J.C. Lee, W.R. Sellers, D. Stokoe, M. Prados, T.F. Cloughesy, C.L. Sawyers, and P.S. Mischel. 2005. Molecular determinants of the response of glioblastomas to EGFR kinase inhibitors. N Engl J Med 353: 2012-2024.

Naito, Y., S. Tsuchiya, S. Ishihara, K. Minato, Y. Shitara, A. Takise, T. Suga, A. Mogi, K. Yamabe, and R. Saito. 2008. Impact of preexisting pulmonary fibrosis detected on chest radiograph and CT on the development of gefitinib-related interstitial lung disease. Am J Clin Oncol 31: 340-344.

Naumov, G.N., M.B. Nilsson, T. Cascone, A. Briggs, O. Straume, L.A. Akslen, E. Lifshits, L.A. Byers, L. Xu, H.K. Wu, P. Janne, S. Kobayashi, B. Halmos, D. Tenen, X.M. Tang, J. Engelman, B. Yeap, J. Folkman, B.E. Johnson, and J.V. Heymach. 2009. Combined vascular endothelial growth factor receptor and epidermal growth factor receptor (EGFR) blockade inhibits tumor growth in xenograft models of EGFR inhibitor resistance. Clin Cancer Res 15: 3484-3494.

Nicholson, R.I., J.M. Gee, and M.E. Harper. 2001. EGFR and cancer prognosis. Eur J Cancer 37 Suppl 4: S9-15.

Ohgaki, H. and P. Kleihues. 2007. Genetic pathways to primary and secondary glioblastoma. Am J Pathol 170: 1445-1453.

Parikh, A.A., W.B. Liu, F. Fan, O. Stoeltzing, N. Reinmuth, C.J. Bruns, C.D. Bucana, D.B. Evans, and L.M. Ellis. 2003. Expression and regulation of the novel vascular endothelial growth factor receptor neuropilin-1 by epidermal growth factor in human pancreatic carcinoma. Cancer 98: 720-729.

Pelloski, C.E., K.V. Ballman, A.F. Furth, L. Zhang, E. Lin, E.P. Sulman, K. Bhat, J.M. McDonald, W.K. Yung, H. Colman, S.Y. Woo, A.B. Heimberger, D. Suki, M.D. Prados, S.M. Chang, F.G. Barker, 2nd, J.C. Buckner, C.D. James, and K. Aldape. 2007. Epidermal growth factor receptor variant III status defines clinically distinct subtypes of glioblastoma. J Clin Oncol 25: 2288-2294.

Perrotte, P., T. Matsumoto, K. Inoue, H. Kuniyasu, B.Y. Eve, D.J. Hicklin, R. Radinsky, and C.P. Dinney. 1999. Anti-epidermal growth factor receptor antibody C225 inhibits angiogenesis in human transitional cell carcinoma growing orthotopically in nude mice. Clin Cancer Res 5: 257-265.

Pillay, V., L. Allaf, A.L. Wilding, J.F. Donoghue, N.W. Court, S.A. Greenall, A.M. Scott, and T.G. Johns. 2009. The plasticity of oncogene addiction: implications for targeted therapies directed to receptor tyrosine kinases. Neoplasia 11: 448-458, 442 p following 458.

Pore, N., S. Liu, D.A. Haas-Kogan, D.M. O'Rourke, and A. Maity. 2003. PTEN mutation and epidermal growth factor receptor activation regulate vascular endothelial growth factor (VEGF) mRNA expression in human glioblastoma cells by transactivating the proximal VEGF promoter. Cancer Res 63: 236-241.

Porta, C., C. Paglino, I. Imarisio, and L. Bonomi. 2007. Uncovering Pandora's vase: the growing problem of new toxicities from novel anticancer agents. The case of sorafenib and sunitinib. Clin Exp Med 7: 127-134.

Reardon D, Cloughesy T, Conrad C, Prados M, Xia J, Mietlowski W, Dugan M, Mischel P, Friedman H, Yung A. 2005 A phase I study of AEE788, a novel multitargeted inhibitor of ErbB and VEGF receptor family tyrosine kinases, in recurrent GBM patients. Journal of Clinical Oncology, 2005 ASCO Annual Meeting Proceedings. Vol 23, No. 16S, Part I of II (June 1 Supplement): 3063

Rich, J.N., D.A. Reardon, T. Peery, J.M. Dowell, J.A. Quinn, K.L. Penne, C.J. Wikstrand, L.B. Van Duyn, J.E. Dancey, R.E. McLendon, J.C. Kao, T.T. Stenzel, B.K. Ahmed Rasheed, S.E. Tourt-Uhlig, J.E. Herndon, 2nd, J.J. Vredenburgh, J.H. Sampson, A.H. Friedman, D.D. Bigner, and H.S. Friedman. 2004. Phase II trial of gefitinib in recurrent glioblastoma. J Clin Oncol 22: 133-142.

Rini, B.I., I. Tamaskar, P. Shaheen, R. Salas, J. Garcia, L. Wood, S. Reddy, R. Dreicer, and R.M. Bukowski. 2007. Hypothyroidism in patients with metastatic renal cell carcinoma treated with sunitinib. J Natl Cancer Inst 99: 81-83.

Rong, Y., D.L. Durden, E.G. Van Meir, and D.J. Brat. 2006a. 'Pseudopalisading' necrosis in glioblastoma: a familiar morphologic feature that links vascular pathology, hypoxia, and angiogenesis. J Neuropathol Exp Neurol 65: 529-539.

Rong, Y., F. Hu, R. Huang, N. Mackman, J.M. Horowitz, R.L. Jensen, D.L. Durden, E.G. Van Meir, and D.J. Brat. 2006b. Early growth response gene-1 regulates hypoxia-induced expression of tissue factor in glioblastoma multiforme through hypoxia-inducible factor-1-independent mechanisms. Cancer Res 66: 7067-7074.

Rosenbaum, S.E., S. Wu, M.A. Newman, D.P. West, T. Kuzel, and M.E. Lacouture. 2008. Dermatological reactions to the multitargeted tyrosine kinase inhibitor sunitinib. Support Care Cancer 16: 557-566.

Rubin, B.P. and A. Duensing. 2006. Mechanisms of resistance to small molecule kinase inhibition in the treatment of solid tumors. Lab Invest 86: 981-986.

Salmaggi, A., M. Eoli, S. Frigerio, A. Silvani, M. Gelati, E. Corsini, G. Broggi, and A. Boiardi. 2003. Intracavitary VEGF, bFGF, IL-8, IL-12 levels in primary and recurrent malignant glioma. J Neurooncol 62: 297-303.

Saltz, L.B., H.J. Lenz, H.L. Kindler, H.S. Hochster, S. Wadler, P.M. Hoff, N.E. Kemeny, E.M. Hollywood, M. Gonen, M. Quinones, M. Morse, and H.X. Chen. 2007. Randomized phase II trial of cetuximab, bevacizumab, and irinotecan compared with cetuximab and bevacizumab alone in irinotecan-refractory colorectal cancer: the BOND-2 study. J Clin Oncol 25: 4557-4561.

Sathornsumetee, S., J. J. Vredenburgh, J. N. Rich, A. Desjardins, J. A. Quinn, A. E. Mathe, S. Gururangan, A. H. Friedman, H. S. Friedman, D. A. Reardon Phase II study of bevacizumab and erlotinib in patients with recurrent glioblastoma multiforme. J Clin Oncol 26: 2008 (May 20 suppl; abstr 13008).

Scagliotti, G.V. 2007. Potential role of multi-targeted tyrosine kinase inhibitors in non-small-cell lung cancer. Ann Oncol 18 Suppl 10: x32-41.

Schwandt A, Wood LS, Rini B, and D. R. 2009. Management of side effects associated with sunitinib therapy for patients with renal cell carcinoma OncoTargets and Therapy 2: 51-61.

Seto, T., N. Seki, K. Uematsu, T. Tanigaki, S. Shioya, T. Koboyashi, S. Umemura, and K. Eguchi. 2006. Gefitinib-induced lung injury successfully treated with high-dose corticosteroids. Respirology 11: 113-116.

Stupp, R., M.E. Hegi, W.P. Mason, M.J. van den Bent, M.J. Taphoorn, R.C. Janzer, S.K. Ludwin, A. Allgeier, B. Fisher, K. Belanger, P. Hau, A.A. Brandes, J. Gijtenbeek, C. Marosi, C.J. Vecht, K. Mokhtari, P. Wesseling, S. Villa, E. Eisenhauer, T. Gorlia, M. Weller, D. Lacombe, J.G. Cairncross, and R.O. Mirimanoff. 2009. Effects of radiotherapy with concomitant and adjuvant temozolomide versus radiotherapy alone on survival in glioblastoma in a randomised phase III study: 5-year analysis of the EORTC-NCIC trial. Lancet Oncol 10: 459-466.

Stupp, R., W.P. Mason, M.J. van den Bent, M. Weller, B. Fisher, M.J. Taphoorn, K. Belanger, A.A. Brandes, C. Marosi, U. Bogdahn, J. Curschmann, R.C. Janzer, S.K. Ludwin, T. Gorlia, A. Allgeier, D. Lacombe, J.G. Cairncross, E. Eisenhauer, and R.O. Mirimanoff. 2005. Radiotherapy plus concomitant and adjuvant temozolomide for glioblastoma. N Engl J Med 352: 987-996.

Tabernero, J. 2007. The role of VEGF and EGFR inhibition: implications for combining anti-VEGF and anti-EGFR agents. Mol Cancer Res 5: 203-220.

Tamura, T., H. Minami, Y. Yamada, N. Yamamoto, T. Shimoyama, H. Murakami, A. Horiike, Y. Fujisaka, T. Shinkai, M. Tahara, K. Kawada, H. Ebi, Y. Sasaki, H. Jiang, and N. Saijo. 2006. A phase I dose-escalation study of ZD6474 in Japanese patients with solid, malignant tumors. J Thorac Oncol 1: 1002-1009.

van Cruijsen, H., G. Giaccone, and K. Hoekman. 2005. Epidermal growth factor receptor and angiogenesis: Opportunities for combined anticancer strategies. Int J Cancer 117: 883-888.

van Cruijsen, H., E.E. Voest, C.J. Punt, K. Hoekman, P.O. Witteveen, M.R. Meijerink, T.A. Puchalski, J. Robertson, O. Saunders, J.M. Jurgensmeier, C.M. van Herpen, and G. Giaccone. 2010. Phase I evaluation of cediranib, a selective VEGFR signalling inhibitor, in combination with gefitinib in patients with advanced tumours. Eur J Cancer 46: 901-911.

Verhaak, R.G., K.A. Hoadley, E. Purdom, V. Wang, Y. Qi, M.D. Wilkerson, C.R. Miller, L. Ding, T. Golub, J.P. Mesirov, G. Alexe, M. Lawrence, M. O'Kelly, P. Tamayo, B.A. Weir, S. Gabriel, W. Winckler, S. Gupta, L. Jakkula, H.S. Feiler, J.G. Hodgson, C.D. James, J.N. Sarkaria, C. Brennan, A. Kahn, P.T. Spellman, R.K. Wilson, T.P. Speed, J.W. Gray, M. Meyerson, G. Getz, C.M. Perou, and D.N. Hayes. 2010. Integrated

genomic analysis identifies clinically relevant subtypes of glioblastoma characterized by abnormalities in PDGFRA, IDH1, EGFR, and NF1. Cancer Cell 17: 98-110.

Viloria-Petit, A., T. Crombet, S. Jothy, D. Hicklin, P. Bohlen, J.M. Schlaeppi, J. Rak, and R.S. Kerbel. 2001. Acquired resistance to the antitumor effect of epidermal growth factor receptor-blocking antibodies in vivo: a role for altered tumor angiogenesis. Cancer Res 61: 5090-5101.

Viloria-Petit, A.M. and R.S. Kerbel. 2004. Acquired resistance to EGFR inhibitors: mechanisms and prevention strategies. Int J Radiat Oncol Biol Phys 58: 914-926.

Vredenburgh, J.J., A. Desjardins, J.E. Herndon, 2nd, J. Marcello, D.A. Reardon, J.A. Quinn, J.N. Rich, S. Sathornsumetee, S. Gururangan, J. Sampson, M. Wagner, L. Bailey, D.D. Bigner, A.H. Friedman, and H.S. Friedman. 2007. Bevacizumab plus irinotecan in recurrent glioblastoma multiforme. J Clin Oncol 25: 4722-4729.

Wacker, B., T. Nagrani, J. Weinberg, K. Witt, G. Clark, and P.J. Cagnoni. 2007. Correlation between development of rash and efficacy in patients treated with the epidermal growth factor receptor tyrosine kinase inhibitor erlotinib in two large phase III studies. Clin Cancer Res 13: 3913-3921.

Wedge, S.R., J. Kendrew, L.F. Hennequin, P.J. Valentine, S.T. Barry, S.R. Brave, N.R. Smith, N.H. James, M. Dukes, J.O. Curwen, R. Chester, J.A. Jackson, S.J. Boffey, L.L. Kilburn, S. Barnett, G.H. Richmond, P.F. Wadsworth, M. Walker, A.L. Bigley, S.T. Taylor, L. Cooper, S. Beck, J.M. Jurgensmeier, and D.J. Ogilvie. 2005. AZD2171: a highly potent, orally bioavailable, vascular endothelial growth factor receptor-2 tyrosine kinase inhibitor for the treatment of cancer. Cancer Res 65: 4389-4400.

Wen, P.Y., D.R. Macdonald, D.A. Reardon, T.F. Cloughesy, A.g. Sorensen, E. Galanis, J. DeGroot, W. Wick, M.R. Gilbert, A.B. Lassman, C. Tsien, T. Mikkelsen, E.T. Wong, M.C. Chamberlain, R. Stupp, K.R. Lamborn, M.A. Vogelbaum, M.J. van den Bent, S.M. Chang. 2010. Updated response criteria for high-grade gliomas: response assessment in neuro-oncology working group. J Clinical Oncology 28: 1963-1972

Wick, W., V.K. Puduvalli, M.C. Chamberlain, M.J. van den Bent, A.F. Carpentier, L.M. Cher, W. Mason, M. Weller, S. Hong, L. Musib, A.M. Liepa, D.E. Thornton, and H.A. Fine. 2010a. Phase III study of enzastaurin compared with lomustine in the treatment of recurrent intracranial glioblastoma. J Clin Oncol 28: 1168-1174.

Wick, W., M. Weller, M. van den Bent, and R. Stupp. 2010b. Bevacizumab and recurrent malignant gliomas: a European perspective. J Clin Oncol 28: e188-189; author reply e190-182.

Wong, A.J., S.H. Bigner, D.D. Bigner, K.W. Kinzler, S.R. Hamilton, and B. Vogelstein. 1987. Increased expression of the epidermal growth factor receptor gene in malignant gliomas is invariably associated with gene amplification. Proc Natl Acad Sci U S A 84: 6899-6903.

Wong, E.T., K.R. Hess, M.J. Gleason, K.A. Jaeckle, A.P. Kyritsis, M.D. Prados, V.A. Levin, and W.K. Yung. 1999. Outcomes and prognostic factors in recurrent glioma patients enrolled onto phase II clinical trials. J Clin Oncol 17: 2572-2578.

Yan, H., D.W. Parsons, G. Jin, R. McLendon, B.A. Rasheed, W. Yuan, I. Kos, I. Batinic-Haberle, S. Jones, G.J. Riggins, H. Friedman, A. Friedman, D. Reardon, J. Herndon, K.W. Kinzler, V.E. Velculescu, B. Vogelstein, and D.D. Bigner. 2009. IDH1 and IDH2 mutations in gliomas. N Engl J Med 360: 765-773.

Yung, W.K., R.E. Albright, J. Olson, R. Fredericks, K. Fink, M.D. Prados, M. Brada, A. Spence, R.J. Hohl, W. Shapiro, M. Glantz, H. Greenberg, R.G. Selker, N.A. Vick, R. Rampling, H. Friedman, P. Phillips, J. Bruner, N. Yue, D. Osoba, S. Zaknoen, and V.A. Levin. 2000. A phase II study of temozolomide vs. procarbazine in patients with glioblastoma multiforme at first relapse. Br J Cancer 83: 588-593.

Appendix 1: Abbreviations

**AE**	Adverse Event
**ALP**	Alkaline phosphatase
**ALT**	Alanine transaminase
**ANC**	Absolute Neutrophil Count
**AR**	Adverse Reaction
**AST**	Aspartate aminotransferase
**AUC**	Area Under the Curve
**CI**	Chief Investigator
**CLS**	Country Lead Site
**CR**	Complete response
**CRF**	Case Report Form
**CTA**	Clinical Trial Authorisation
**CTAAC**	Clinical Trials Advisory & Awards Committee
**CTCAE**	National Cancer Institute Common Terminology Criteria for Adverse Events
**CTSA**	Clinical Trial Site Agreement
**DPA**	Data Protection Act
**ECG**	Electrocardiogram
**ECOG**	Eastern Cooperative Oncology Group
**EGF**	Epidermal growth factor
**EGFR**	Epidermal growth factor receptor (ErbB1)
**EGFR vIII**	Epidermal growth factor receptor variant III
**EMEA**	European Medicines Agency
**EudraCT**	European Clinical Trials Database
**FBC**	Full Blood Count
**FDA**	US Food and Drug Administration
**GBM**	Glioblastoma
**GFR**	Glomerular Filtration Rate
**Hb**	Haemoglobin
**IB**	Investigators Brochure
**ICH GCP**	International Conference of Harmonisation-Good Clinical Practice
**IDH**	Isocitrate dehydrogenase
**IDMC**	Independent Data Monitoring Committee
**ILD**	Interstitial lung disease
**IMP**	Investigational Medicinal Product
**INR**	International Normalised Ratio
**ISRCTN**	International Standard Randomised Controlled Trial Number
**ITSC**	Independent Trial Steering Committee
**IV**	Intravenous
**IWRS**	Interactive web-based recognition system
**LDH**	Lactate Dehydrogenase
**LFT**	Liver Function Tests
**LLN**	Lower Limit of Normal
**MGMT**	O^6-methylguanine-DNA methyltransferase
**MTOR**	Mammalian target of rapamycin
**MRC**	Medical Research Council
**MRI**	Magnetic Resonance Image
**MHRA**	Medicines and Healthcare products Regulatory Agency
**NCRI**	National Cancer Research Institute
**NCRN**	National Cancer Research Network
**NRES**	National Research Ethics Service

**NSCLC**	Non small cell lung cancer
**OS**	Overall Survival
**PD**	Progressive Disease
**PDGFR**	Platelet derived growth factor receptor
**PFS**	Progression Free Survival
**PI**	Principal Investigator
**PO**	By mouth
**PPE**	Palmar plantar erythrodysesthesia
**PR**	Partial Response
**PTEN**	Phosphatase and tensin homolog
**RANO**	Response Assessment for Neuro-Oncology
**REC**	Research Ethics Committee
**RPLS**	Reversible posterior leucoencephalopathy syndrome
**SAE**	Serious Adverse Event
**SD**	Stable Disease
**SmPC**	Summary of Product Characteristics
**SSA**	Site Specific Assessment
**SUSAR**	Suspected Unexpected Serious Adverse Reaction
**TKI**	Tyrosine kinase inhibitor
**TMF**	Trial Master File
**TMG**	Trial Management Group
**UCL CTC**	CR UK and UCL Cancer Trials Centre
**ULN**	Upper Limit of Normal
**ULRR**	Upper limit of reference range
**UPC**	Urine protein creatinine
**VEGF**	Vascular endothelial growth factor
**VEGFR**	Vascular endothelial growth factor receptor
**WBC**	White Blood Cells

Appendix 2: Recommended Skin Care products

Skin care products Product information (Schwandt A et al. 2009)

Skin care products	Product information
Cetaphil skin cleanser, Aveeno® shower gel	Non-deodorant, non-fragrant body washes
Udderly Smooth® udder cream	Allantoin, dimethicone
Udderly Smooth® extra care cream	Allantoin, dimethicone, 10% urea
Aveeno® skin relief moisturizing cream	Natural colloidal oatmeal; dimethicone
Norwegian Formula: soothing relief anti-itch moisturizer by Neutrogena	Dimethicone 1%, camphor 0.1%, and lidocaine
Norwegian Formula: foot cream by Neutrogena	Ceterayl alcohol, dimethicone, menthol and urea
Bag Balm®	8-hydroxyquinoline sulfate 0.3% in a petrolatum lanolin base; eucalyptus
Eucerin® cream	Mineral oil, lanolin
Eucerin® dry skin therapy	Urea and alpha hydroxy acid
Aquaphor® healing ointment	41% petrolatum
Xenaderm® ointment	Balsam peru, castor oil, trypsin
Corn Huskers Lotion®	Calcium alginate; non-oily moisturizer
Biafine® cream	Water-based emulsion for radiation dermatitis or abrasions
Gold Bond® triple action relief cream	5% dimethicone, 0.15% menthol, aloe, vitamin E
Gold Bond® ultimate healing lotion	5% dimethicone, jojoba esters, aloe
Gold Bond® anti-itch cream	1% pramoxine HCL, 1% menthol
Kerasal™	5% salicylic acid: softens skin 10% urea: exfoliates and moisturizes
Keralac™ cream	50% urea: exfoliates and moisturizes vitamin E, lactic acid, zinc
Keralac™ lotion	35% urea: exfoliates and moisturizes vitamin E, lactic acid, zinc
Carmol® 40	40% urea
Miracle Foot Repair® cream	60% aloe, 0.1% menthol
Regenecare® HA	2% lidocaine, humectanct (moisturizer), aloe vera extract, hyaluronic acid (anti-inflammatory agent)
Lidocaine topical	2% lidocaine
Lidamantle®	3% lidocaine HCL
Lidamantle HC®	3% lidocaine HCL, 0.5% hydrocortisone acetate
Pramocaine HCL	1% pramoxine hydrochloride
Tetracaine jelly	2% lidocaine

Appendix 3: Recommended Oral Care products

Oral care products

Cleansers and rinses	Product information
Salt water rinse	
Salt and baking soda rinse	
Biotine® rinse	Bioactive enzymes
Chlorhexidine gluconate 0.12%	Antimicrobial mouth rinse
**Toothpastes**	
Sensodyne® toothpaste	Potassium nitrate to reduce painful sensitivity
Children's toothpaste	Flavoring is milder and burns less
**Rinses for comfort and mucosal protection**	
Rincinol™ PRN	Aloe vera rinse
Gelclair® bioadherent oral gel	Hydrates, soothes, and protects oral mucosa
Lidocaine Viscous	2% lidocaine hydrocholoride solution
**Lip protectants**	
Blistex®	Dimethicone
Burt's Bees®	Beeswax, oils, lanolin
Carmex®	Menthol, camphor, phenol
Zim's Crack Creme®	Arnica

Appendix 4: Drugs thought to significantly interact with CYP450 enzymes

These drugs are recommended to be avoided in combination with cediranib and/or gefitinib, and for 2 weeks prior to starting trial treatment.

Enzyme	Potent Inhibitors*	Potent Inducers**	Substrates†
CYP1A2	Amiodarone,<br>cimetidine,<br>ciprofloxacin,<br>fluvoxamine	Carbamazepine ,<br>phenobarbital,<br>rifampin,<br>tobacco	Caffeine,<br>clozapine,<br>theophylline
CYP2C9	Amiodarone,<br>fluconazole,<br>fluoxetine,<br>metronidazole,<br>ritonavir,<br>trimethoprim /sulfamethoxazole	Carbamazepine,<br>phenobarbital,<br>phenytoin,<br>rifampin	Carvedilol,<br>celecoxib,<br>glipizide,<br>ibuprofen,<br>irbesartan,<br>losartan
CYP2C19	Fluvoxamine,<br>isoniazid,<br>ritonavir	Carbamazepine,<br>phenytoin,<br>rifampin	Omeprazole,<br>phenobarbital,<br>phenytoin
CYP2D6	Amiodarone,<br>cimetidine,<br>diphenhydramine,<br>fluoxetine,<br>paroxetine,<br>quinidine,<br>ritonavir,<br>terbinafine	No significant inducers	Amitriptyline,<br>carvedilol,<br>codeine,<br>donepezil,<br>haloperidol,<br>metoprolol,<br>paroxetine,<br>risperidone,<br>tramadol
CYP3A4<br>and<br>CYP3A5	Clarithromycin,<br>diltiazem,<br>erythromycin,<br>**grapefruit juice,**<br>itraconazole,<br>ketoconazole,<br>nefazodone,<br>ritonavir,<br>telithromycin,<br>verapamil	Carbamazepine,<br>Hypericum perforatum<br>(St. John's wort),<br>phenobarbital,<br>phenytoin,<br>rifampin	Alprazolam,<br>amlodipine,<br>atorvastatin,<br>cyclosporine,<br>diazepam,<br>estradiol,<br>simvastatin,<br>sildenafil,<br>verapamil,<br>zolpidem

* These will slow down substrate drug metabolism and increase drug effect.
** These will speed up substrate drug metabolism and decrease drug effect
† Exposure, pharmacological action and toxicity may be increased in combination with cediranib and/or gefitinib

This list is not exhaustive and a similar restriction will apply to other agents that are known to strongly induce, inhibit CYP450 enzyme activity or require CYP450 for metabolism. For an extensive list including less significant interactions see http://medicine.iupui.edu/clinpharm/ddis/table.asp.

Table compiled from: *Lynch T, Price A. (2007) The effect of cytochrome P450 metabolism on drug response, interactions, and adverse effects. Am Fam Physician. Aug 1;76(3):391-6.*

Appendix 5: Performance Status Evaluation

ECOG PERFORMANCE STATUS

Grade	ECOG
0	Fully active, able to carry on all pre-disease performance without restriction
1	Restricted in physically strenuous activity but ambulatory and able to carry out work of a light or sedentary nature, e.g., light house work, office work
2	Ambulatory and capable of all selfcare but unable to carry out any work activities. Up and about more than 50% of waking hours
3	Capable of only limited selfcare, confined to bed or chair more than 50% of waking hours
4	Completely disabled. Cannot carry on any selfcare. Totally confined to bed or chair
5	Dead

Oken, M.M., Creech, R.H., Tormey, D.C., Horton, J., Davis, T.E., McFadden, E.T., Carbone, P.P.: Toxicity And Response Criteria Of The Eastern Cooperative Oncology Group. Am J Clin Oncol 5:649-655, 1982.

KARNOFSKY PERFORMANCE STATUS SCALE DEFINITIONS RATING (%) CRITERIA

Able to carry on normal activity and to work; no special care needed.	100	Normal no complaints; no evidence of disease.
	90	Able to carry on normal activity; minor signs or symptoms of disease.
	80	Normal activity with effort; some signs or symptoms of disease.
Unable to work; able to live at home and care for most personal needs; varying amount of assistance needed.	70	Cares for self; unable to carry on normal activity or to do active work.
	60	Requires occasional assistance, but is able to care for most of his personal needs.
	50	Requires considerable assistance and frequent medical care.
Unable to care for self; requires equivalent of institutional or hospital care; disease may be progressing rapidly.	40	Disabled; requires special care and assistance.
	30	Severely disabled; hospital admission is indicated although death not imminent.
	20	Very sick; hospital admission necessary; active supportive treatment necessary.
	10	Moribund; fatal processes progressing rapidly.
	0	Dead

Schag CC, Heinrich RL, Ganz PA. Karnofsky performance status revisited: Reliability, validity, and guidelines. J Clin Oncology. 1984; 2:187-193.

Appendix 6: Quality of Life Assessments

EORTC QLQ-C30 (version 3)

We are interested in some things about you and your health. Please answer all of the questions yourself by circling the number that best applies to you. There are no "right" or "wrong" answers. The information that you provide will remain strictly confidential.

Please fill in your initials: bbbb Your birthdate (Day, Month, Year): ddmmyyyy
Today's date (Day, Month, Year): ddmmyyyy

Not at All (1), A little (2) Quite a Bit (3) Very Much (4)

1 Do you have any trouble doing strenuous activities, like carrying a heavy shopping bag or a suitcase? 1 2 3 4

2 Do you have any trouble taking a <u>long</u> walk? 1 2 3 4

3 Do you have any trouble taking a <u>short</u> walk outside of the house? 1 2 3 4

4 Do you need to stay in bed or a chair during the day? 1 2 3 4

5 Do you need help with eating, dressing, washing yourself or using the toilet? 1 2 3 4

During the past week: Not at All (1), A little (2) Quite a Bit (3) Very Much (4)

6. Were you limited in doing either your work or other daily activities? 1 2 3 4

7. Were you limited in pursuing your hobbies or other leisure time activities? 1 2 3 4

8. Were you short of breath? 1 2 3 4

9. Have you had pain? 1 2 3 4

10. Did you need to rest? 1 2 3 4

11. Have you had trouble sleeping? 1 2 3 4

12. Have you felt weak? 1 2 3 4

13. Have you lacked appetite? 1 2 3 4

14. Have you felt nauseated? 1 2 3 4

15. Have you vomited? 1 2 3 4

16. Have you been constipated? 1 2 3 4

Please go on to the next page

During the past week: **Not at All (1), A little (2) Quite a Bit (3) Very Much (4)**

17. Have you had diarrhoea? 1 2 3 4

18. Were you tired? 1 2 3 4

19. Did pain interfere with your daily activities? 1 2 3 4

20. Have you had difficulty in concentrating on things, like reading a newspaper or watching television? 1 2 3 4

21. Did you feel tense? 1 2 3 4

22. Did you worry? 1 2 3 4

23. Did you feel irritable? 1 2 3 4

24. Did you feel depressed? 1 2 3 4

25. Have you had difficulty remembering things? 1 2 3 4

26. Has your physical condition or medical treatment interfered with your family life? 1 2 3 4

27. Has your physical condition or medical treatment interfered with your social activities? 1 2 3 4

28. Has your physical condition or medical treatment caused you financial difficulties? 1 2 3 4

For the following questions please circle the number between 1 and 7 that best applies to you

29. How would you rate your overall health during the past week? 1 2 3 4 5 6 7 (Very poor-Excellent)

30. How would you rate your overall quality of life during the past week? 1 2 3 4 5 6 (Very poor-Excellent)

© Copyright 1995 EORTC Quality of Life Group. All rights reserved. Version 3.0

EORTC QLQ -BN20

Patients sometimes report that they have the following symptoms. Please indicate the extent to which you have experienced these symptoms or problems during the past week.

During the past week: Not at All (1), A little (2) Quite a Bit (3) Very Much (4)

31. Did you feel uncertain about the future? 1 2 3 4
32. Did you feel you had setbacks in your condition? 1 2 3 4
33. Were you concerned about disruption of family life? 1 2 3 4
34. Did you have headaches? 1 2 3 4
35. Did your outlook on the future worsen? 1 2 3 4
36. Did you have double vision? 1 2 3 4
37. Was your vision blurred? 1 2 3 4
38. Did you have difficulty reading because of your vision? 1 2 3 4
39. Did you have seizures? 1 2 3 4
40. Did you have weakness on one side of your body? 1 2 3 4
41. Did you have trouble finding the right words to express yourself? 1 2 3 4
42. Did you have difficulty speaking? 1 2 3 4
43. Did you have trouble communicating your thoughts? 1 2 3 4
44. Did you feel drowsy during the daytime? 1 2 3 4
45. Did you have trouble with your coordination? 1 2 3 4
46. Did hair loss bother you? 1 2 3 4
47. Did itching of your skin bother you? 1 2 3 4
48. Did you have weakness of both legs? 1 2 3 4
49. Did you feel unsteady on your feet? 1 2 3 4
50. Did you have trouble controlling your bladder? 1 2 3 4

© Copyright 1994 EORTC Study Group on Quality of Life. (phase III module)

Appendix 7: QTc with Bazetts Correction

$$QTcB\ (mSec) = \frac{QT(mSec) \times 0.04}{\sqrt{(RR\ (mSec) \times 0.04)}}$$

QTc: QT Interval (time between the start of the Q wave and the end of the T wave on the ECG trace)

RR: interval from the onset of one QRS complex to the onset of the next QRS complex, often derived from the heart rate (HR) as 60/HR

Mean QTcB to be calculated, e.g from a prolonged rhythm strip.

Appendix 8: MRI Acquisition form

Circle Visit:

Baseline	Week 6	Week 12	Week 18	Week 24	Week 30	Week 36	Week 42
Week 48	Week 54	Week 60	Week 66	Week 72	Week 78	Week 84	Week 90
Week 96	Week 102	Week 108	Week 114	Week 120	Other:		Discontinuation

Patient Trial Number: DRC................
Patient Initials:................................
Date of Scan: ....
Hospital: ....

IMAGE		Orientation	Pulse parameters			Flip Angle	Matrix	FOV	Slice gap (mm)	Slice thickness (mm)	No of acquisitions
			TR (Repetition Time) /msec	TE (Echo time) /msec	TI (Inversion time) /msec						
FLAIR (Fluid-attenuated inversion-recovery image)	Required Parameters	Axial							0mm	3	
	Actual parameters										
DWI (diffusion weighted image) Spin echo –echo planar	Required Parameters	Axial							0mm	3	
	Actual parameters										
T2 (T2-weighted fast spin echo images)	Required Parameters	Axial							0mm	3	
	Actual parameters										
T1 volumetric (three-dimensional gradient echo images)	Required Parameters	Coronal							0mm	1	
	Actual parameters										
**Post-contrast** T1 volumetric (three-dimensional gradient echo images)	Required Parameters	Coronal							0mm	1	
	Actual parameters										
**Post-contrast** FLAIR (Fluid-attenuated inversion-recovery image)	Required Parameters	Axial							0mm	3	
	Actual parameters										

Staff member completing form:.......................................

Please return this form with a copy of the scans on CD in DICOM format to the DORIC Trial Coordinator, CR-UK & UCL Cancer Trials Centre, 90 Tottenham Court Road, London W1T 4TJ.

Appendix 9: Protocol Version History

Protocol:		Amendments:		
**Version no.**	**Date**	**Amendment no.**	**Protocol Section (no./title)**	**Summary of main changes from previous version.**
1.0	30.11.10	N/A	N/A	N/A
